# Colonoscopy in poorly prepped colons: a cost effectiveness analysis comparing standard of care to a new cleansing technology

**DOI:** 10.1186/s12962-021-00277-5

**Published:** 2021-04-29

**Authors:** Jeffrey Voigt, Michael Mosier, Ian M. Gralnek

**Affiliations:** 1 Medical Device Consultants of Ridgewood, Ridgewood, NJ USA; 2 Department of Mathematics and Statistics, Washburn University, Topeka, KS USA; 3 Rappaport Faculty of Medicine, Technicon Israel Institute of Technology, Haifa, Israel; 4 Institute of Gastroenterology and Hepatology, Emek Medical Center, Afula, Israel

**Keywords:** Colonoscopy, Markov model, Cost effectiveness

## Abstract

**Background:**

The objective of this Markov model lifetime cost-effectiveness analysis was to evaluate a new medical device technology which minimizes redo colonoscopies on the outcomes of cost, quality of life, and aversion of colorectal cancers (CRC).

**Methods:**

A new technology (PureVu® System) which cleans inadequately prepped colons was evaluated using TreeAge 2019 software in patients who presented with inadequate prep in outpatient settings in the US. PureVu was compared to the standard of care (SOC). Peer reviewed literature was used to identify the CRC incidence cancers based on missing polyps. Costs for procedures were derived from 2019 Medicare and from estimated private payer reimbursements. Base case costs, sensitivity analysis and incremental cost effectiveness (ICE) were evaluated. The cost of PureVu was $750.

**Results:**

Assuming a national average compliance rate of 60% for colonoscopy, the use of PureVu saved the healthcare system $833–$992/patient depending upon the insurer when compared to SOC. QALYs were also improved with PureVu mainly due to a lower incidence of CRCs. In sensitivity analysis, SOC becomes less expensive than PureVu when compliance to screening for CRC using colonoscopy is ≤ 28%. Also, in order for SOC to be less expensive than PureVu, the list price of PureVu would need to exceed $1753. In incremental cost effectiveness analysis, PureVu dominated SOC.

**Conclusion:**

Using the PureVu System to improve bowel prep can save the healthcare system $3.1–$3.7 billion per year, while ensuring a similar quality of life and reducing the incidence of CRCs.

**Supplementary Information:**

The online version contains supplementary material available at 10.1186/s12962-021-00277-5.

## Introduction

Based on the American Cancer Society facts and figures, greater than 135,000 newly diagnosed cases of colorectal cancer (CRC) occurred last year [1]. Early detection methods, such as colonoscopy (defined as the gold standard for detection/diagnosis) have helped to significantly reduce the incidence of CRC [2, 3]. It is estimated that upwards of 15 million undergo screening, surveillance, or diagnostic colonoscopy annually in the United States (US) [4].

One of the main issues facing gastroenterologists is the adequacy of bowel preparation. Proper bowel preparation can decrease the risk of colon cancer by 76–90% [5, 6]. Bowel preparation via colonic cleansing agents can be problematic due to: poor adherence to instructions, improper timing of bowel purgative administration, compromised motility and long wait times for colonoscopy [7]. This results in a ~ 25% failure rate to complete a high quality colonoscopy [8]. Miss rates of polyp detection have been found to be as high as 42–48% in poorly prepped colons [9–11]. Inadequate bowel preparation may also result in procedure rescheduling and increased costs [12] and; an increased likelihood of complications. Inadequate bowel preparation also may result in a lower repeat rate of colonoscopy [13]. The rate of detection of polyps is significantly associated with the risk of CRC [14]. The colonoscopy procedure can also affect the quality of life over the life of the patient (as measured via quality adjusted life year or QALYs [15, 16]).

The PureVu System is a 510(k) US Food and Drug Administration cleared medical device indicated for cleaning a poorly prepped colon during the procedure [17]. PureVu is an add-on “oversleeve” that fits standard colon endoscopes. It delivers a pulsed irrigation of water and air that breaks up fecal matter and evacuates the content in inadequately prepped colons. Results from peer reviewed articles have demonstrated in inadequately prepped colons that PureVu was able to properly clean 95% of colons allowing for an adequate colonoscopy to be performed [18, 19]. The purpose of this study is to examine PureVu examine in a Markov lifetime cost-effectiveness analysis comparing the use of PureVu to standard of care (SOC) for patients undergoing an outpatient colonoscopy in the United States.

## Methods

The Consolidated Health Economic Evaluation Reporting Standards (CHEERS) checklist was used (Additional file 1: Appendix S1). A lifetime Markov model was developed using cost-effectiveness analysis software (TreeAge Pro 2019; Williamstown, MA, USA). The Markov model examined those patients at average risk for CRC. The model followed these patients over their expected life for the care associated with diagnostic colonoscopy ± CRC. Four (4) different models were developed and analyzed: average risk Medicare with and without PureVu and; average risk private pay with and without PureVu. In average risk patients, it was assumed that a colonoscopy was performed every 10 years [3]. A typical 60 year old person was chosen based on logistic regression and propensity matching as described below. The life expectancy for an average risk 60 year old was 24 years [20, 21]. The US “average” 60 year old had the following baseline characteristics which determined their life span for use in the model: for male: 196 lbs; 5′9″ tall and with hypertension and high cholesterol; for female: 163 lbs; 5′3″ tall and with the same comorbidities [20, 22, 23]. For patients with early stage CRC, 23 years was the average life expectancy [20] and; for patients with late/advanced stage CRC, life expectancy was ~ 4.8 years [21] (see Additional file 2: Appendix S2). Thus the model was run per the probability of being in various conditions/states [e.g. no cancer (screening/surveillance), early stage cancer] over their remaining lives post colonoscopy.

It was further assumed that PureVu was used only in the 25% of patients who presented with inadequately prepped colons [24] in each of the above groups at an additional cost to the procedure in using PureVu of $750 [25]. Based on a systematic review of the literature which demonstrated a 2–2.1% inadequate bowel prep with PureVu [18, 19] and; to be conservative, the assumption was made in the Markov model that in 5% of all cases in which PureVu was used, inadequate bowel prep occurred.

In order to ensure matching of patients, propensity scores were estimated from the study data included in the Markov model; and from which patients were introduced into the treatment arms of the model (PureVu or SOC). The propensity score generated was the probability that a patient would have been assigned to the PureVu vs. SOC arm given a set of independent covariates. By matching patients with similar propensity scores, approximate balance could be achieved on the independent covariates, thus minimizing confounding (and a spurious or biased finding of the Markov model). A logistic regression model was developed in which the dependent variable was the outcome was a successfully completed colonoscopy (1 = yes or 100% completed; 0 = no) and the independent variables which affected a successful colonoscopy [age, sex, PureVu (yes or no) and whether the cecum was reached (0–1; with 1 being yes and 0 being no on a continuous scale)] were included. These independent variables were derived from the literature in determining a successfully completed colonoscopy [26, 27]. It was found in the logistic regression analysis that age and sex were significant predictors of the outcome (successful colonoscopy). These variables (sex and age) were controlled for via matching propensity scores of the studies used and; whose variables were included in the Markov model (Additional file 3: Appendix S3 for propensity scores).

The probabilities of compliance to screening (initial and repeat) [1], surveillance [28], repeat colonoscopy (after inadequate bowel prep) [29], adequate bowel prep resulting in a true positive or true negative [30], complication rates [31], probability of death from early and late stage CRC in an average 60 year old were derived from the literature. As mentioned above, an “average” 60 year old (male and female with a distribution of 54% male/46% female) were chosen based on the availability of data that could be matched in propensity scoring and; then used in the model. This breakout by age and sex is consistent with other meta-analyses and large published registries [11, 32]. The published papers utilized in the model were derived from PubMed searches as found in the Additional file 4: Appendix S4 and; variables identified/used in the model were from large populations of patients and represented consistent baseline characteristics with those patients entered into the model. The variables and distributions used in each of the Markov models can be found in Additional file 5: Appendix S5 and Additional file 6: Appendix S6. The Additional file 7: Appendix S7 identifies the cost equations used in the Markov Model for SOC.

**Fig. 1:**
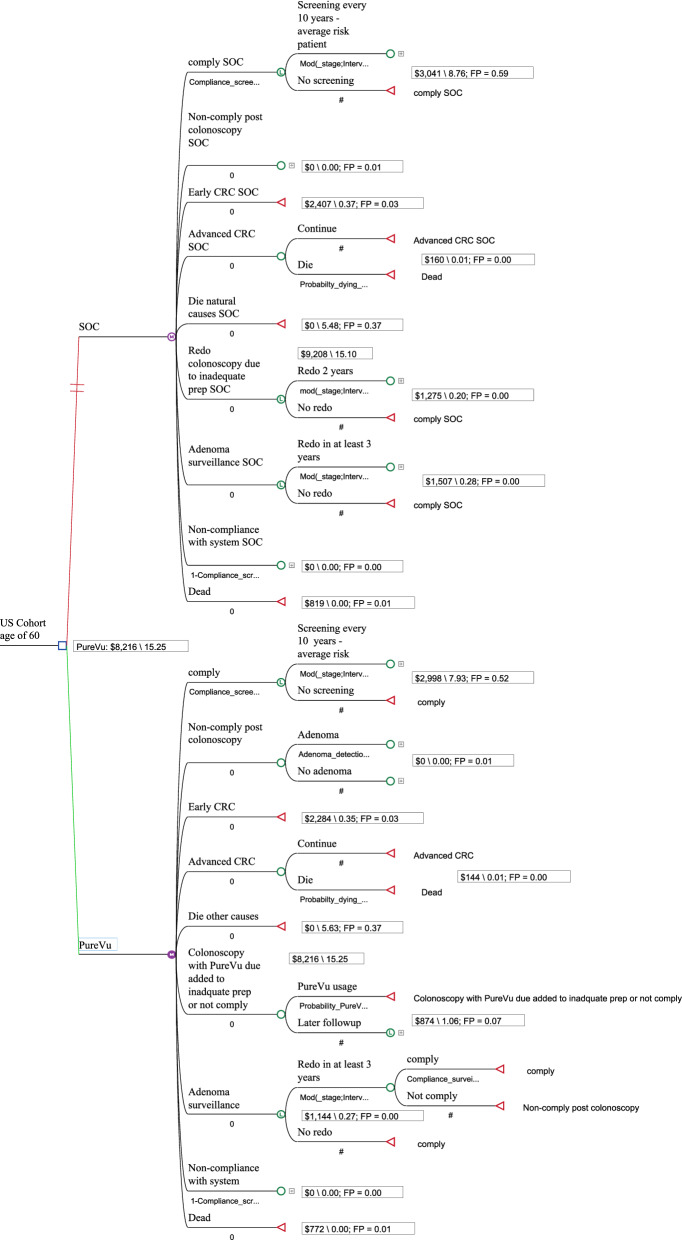
Markov Model Structure

Direct costs for care included: diagnostic colonoscopies, colonoscopies that removed polyps/adenomas, complications resulting from colonoscopy procedures, physician related costs, and the treatment costs of cancer (at various stages). Costs for Medicare and for private pay were derived from Medicare fee schedules or from the literature and if needed; inflated to the year 2019 [33, 34]. Costs for private payer colonoscopies were derived from several sources and included a range of private payer reimbursement rates relative to Medicare of 163–248% [35–38]. Future costs were discounted back to the present at a rate of 3% [39]. The model can be seen in Fig. 1 (Markov Model Structure).

As it relates to quality of life (QoL), the state or condition the patient was in—i.e. no CRC, early stage CRC, metastatic CRC, remission, and the length of time a patient was in that state were derived from the literature [15, 16, 40, 41]. These QoL measurements were then summed as QALYs and discounted at 3% [39]. Lastly sensitivity analyses were performed on inadequate preparation rates and compliance rates to colonoscopy.

In order to evaluate the robustness of the Markov model, a probabilistic sensitivity analysis was run 1728 times using Monte Carlo simulation where the variables and distributions and their corresponding uncertainty (variability) identified in Additional file 4: Appendix S4 and Additional file 5: Appendix S5 were used. This number of iterations were performed in order to ensure that primary output of the model (i.e. mean ICER) did not change by more than 0.1% thereafter. An incremental cost effectiveness graph comparing PureVu to SOC was also evaluated.

## Results

The following lifetime baseline costs and QALYs were calculated via the Markov Model based on the status of the patient and payer and; a price for PureVu of $750 (Table 1). The Markov model states, costs, and outcomes for each state the patient is in (i.e. comply with guidelines for colonoscopy, non-comply post colonoscopy, early colorectal cancer, advanced colorectal cancer, die other cause, adenoma surveillance, non-compliance with system) over time (stages or expected life of patient) are identified in the Additional file 8: Appendix S8 for PureVu use. As can be seen in the Additional file 8: Appendix S8, each state has been assigned a cost and QALY over the corresponding stages. The costs and QALYs assumed an inadequate prep rate of 25% for SOC and 5% for PureVu. The base model also assumed a compliance rate to colonoscopy of 60% [1].

**Table 1:** Lifetime costs and QALYs base case SOC scenario, inadequate prep rate of 25% and 60% compliance to colonoscopy

Patient profile	PureVu costs/QALYs	SOC costs/QALYs	Payer
60 year old average risk	$6070/15.25 ($398)	$6903/15.10 ($457)	Medicare
60 year old average risk	$8216/15.25 ($539)	$9208/15.10 ($610)	Private Pay

Number in parentheses, identifies the cost/QALY

*QALY* quality adjusted life year, *SOC* standard of care

**Fig. 2:**
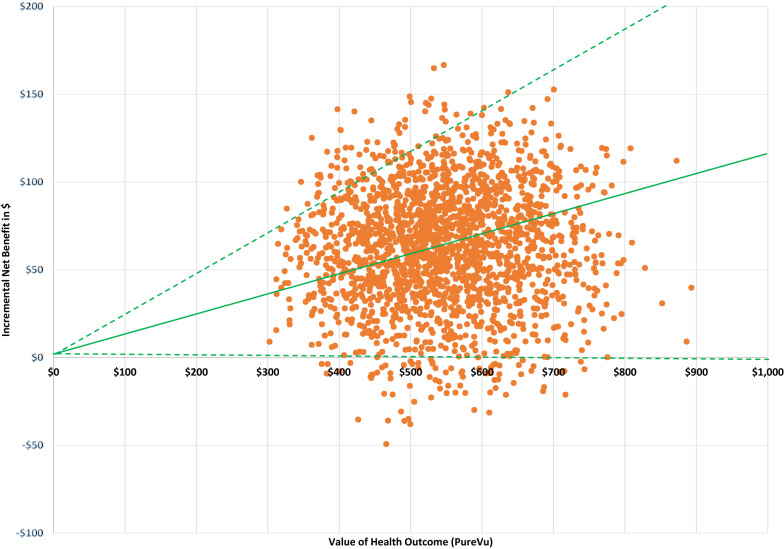
Incremental net benefit in $ of PureVu

The results show that PureVu “dominates” SOC in being less costly and providing a higher quality of life (as measured in quality adjusted life years or QALYs) (Fig. 2 Incremental cost effectiveness scatterplot). The scatterplot in Fig. 2 when running a Monte Carlo simulation 1728 times, shows the relationship between the monetary valuation of the PureVu health outcome and the monetary incremental net benefit (PureVu cost/QALY less SOC cost/QALY) with a 95% confidence limit. The vast majority of the time the incremental net benefit in monetary terms is > $0.

**Fig. 3:**
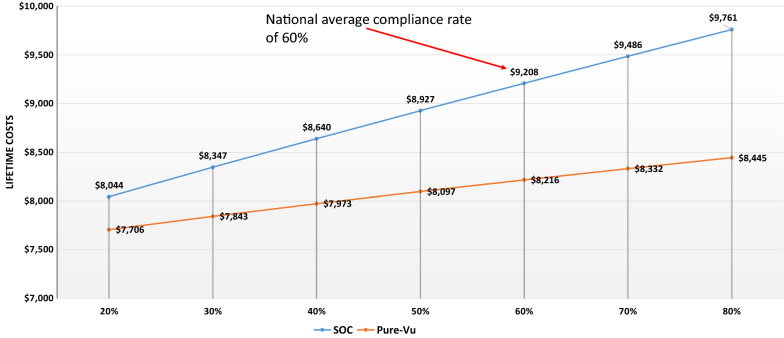
Lifetime costs for patients at average risk for CRC: standard of care (SOC) vs. Pure-Vu: assumes an inadequate prep rate of 25% for SOC and 5% for Pure-Vu with varying compliance rates; Pure-Vu @ $750; private pay

At the baseline assumed rates of inadequate prep of 5% for PureVu and 25% for SOC, PureVu had 1.1% and 0.2% lower absolute incidence rates vs. SOC for early and advanced CRC in the patients studied respectively. Thus, as compliance rates to colonoscopy varied, so did overall lifetime costs of care favoring PureVu (Fig. 3 Lifetime costs average risk CRC SOC vs. PureVu). This was mainly due to the increased cost of colonoscopy as compliance increased and in increased costs for treating missed CRCs with SOC vs. PureVu.

**Fig. 4:**
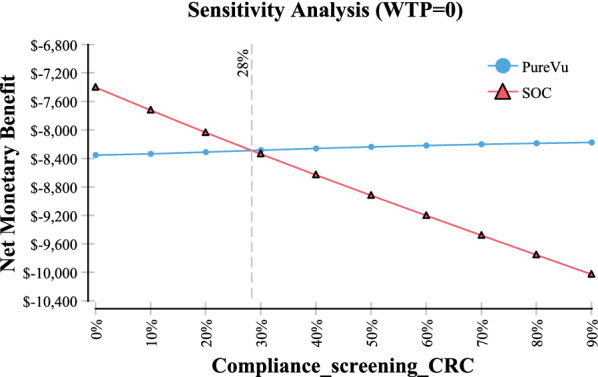
Sensitivity Analysis–Compliance to Screening Colonoscopy

**Fig. 5:**
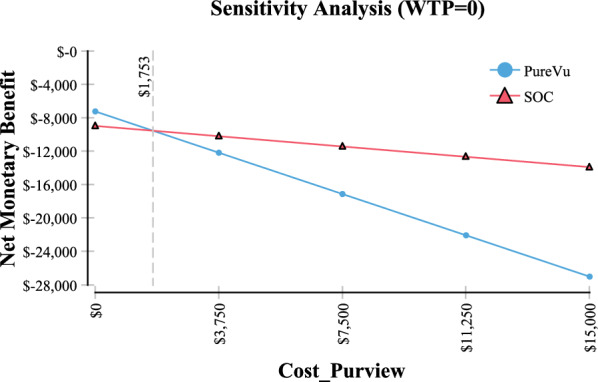
Sensitivity Analysis–Cost PureVu

In sensitivity analysis it was found that the threshold value of when SOC becomes less expensive than PureVu is when compliance is ≤ 28% (Fig. 4 Compliance screening CRC). Further, in sensitivity analysis, SOC became the less expensive option when the cost of PureVu exceeded $1753 (Fig. 5 Sensitivity analysis cost of PureVu). The base price of PureVu used in the model was assumed to be $750.

Costs/QALY based on compliance rates can be found in Table 2.

**Table 2:** Costs per QALY, Private pay payment rates

Compliance rates to colonoscopy (%)	SOC Costs/QALYs	PureVu Costs/QALYs	Cost/QALY savings using PureVu
20	$8044/14.53 ($554)	$7706/14.84 ($519)	$35
30	$8347/14.60 ($572)	$7843/14.87 ($527)	$45
40	$8640/14.73 ($587)	$7973/14.96 ($533)	$54
50	$8927/14.90 ($599)	$8097/15.09 ($536)	$63
60	$9208/15.10 ($610)	$8216/15.25 ($539)	$71
70	$9486/15.33 ($619)	$8332/15.43 ($540)	$79
80	$9761/15.58 ($627)	$8445/15.63 ($540)	$87

Number in parentheses identifies cost per QALY

## Discussion

By improving upon the inadequate bowel prep rate with PureVu, associated lifetime costs and projected incidences of early and late stage CRC were lower when compared to SOC. QALYs were maintained/improved—even at an additional cost of $750 for the PureVu device. This was due mainly to an increased identification rate of polyps/adenomas. It has been found in prior studies that the miss rate of polyps/adenomas (and advanced adenomas) increased significantly as the bowel prep rate declined from excellent to poor/inadequate [11, 42–44].

Approximately 25% of 15 million colonoscopies performed annually, or 3.75 million are inadequately prepped [4]. A repeat colonoscopy occurs on average within 4 years for 12.6% of commercial and 19.8% for Medicare patients [45]. Therefore, in the US, approximately 400,000 patients in the Medicare population (2 million colonoscopies X 25% inadequate bowel prep X 80% [100% less 19.8% non-repeat colonoscopies within 4 years] = 400,000) fail the guidelines. For commercial patients 2.83 million patients fail the guidelines (13 million colonoscopies X 25% inadequate bowel prep X 87% [100% less 12.6%]; with a non-repeat colonoscopy within 4 years). Further, if one assumes an adenoma detection rate of 20% in patients undergoing colonoscopy (which is the proposed threshold value for adenoma detection via colonoscopy [46–48]), then approximately 3.23 million (400,000 Medicare plus 2.83 million commercial) X 20% = 650,000 patients have an adenoma that has not been detected or removed for 4 years. Results from prior trials suggest that colorectal cancer can be prevented by colonoscopic removal of identified adenomas, a finding that supports adenomas progressing to adenocarcinomas [49]. While some of these 650,000 patients may go on to a colonoscopy in the future (e.g. via surveillance, estimated @ 50% after 3 years [29]), an estimated 325,000 (650,000 X 0.50) patients with an adenoma and no follow-up still represents a large number at increased risk for CRC.

Assuming 3.75 million colonoscopies are suboptimal (25% of 15 million), and PureVu on average saves $833–$992/patient (Medicare/private pay), this results in $3.1–$3.7 billion in savings to the healthcare system (3.75 million patients X $833 to $992) over the life of these patients. This is also assuming the same number of colonoscopies are performed every year into the foreseeable future.

As it relates to cost/QALY, in all cases (Medicare and private pay), PureVu resulted in an improved cost/QALY with a similar or higher QALY than SOC. In the above scenarios, repeat colonoscopies for an inadequately prepped colon occurred approximately 55–60% of the time within 3 years [29]. Considering > 40–45% of inadequately prepped colons are not repeated within this time frame [29] and that approximately 20–30% of patients have polyps/adenomas[50], 8–13.5% of patients (40% X 20%; 45% X 30%) are at risk for the adenoma developing into CRC without adequate follow-up. This issue is reflected in a recently published large (> 250,000) patient registry over a median 7.9 years, where there was a doubling of the cancer incidence vs. adequately prepped colons, irrespective of polyp characteristics [51].

While indirect costs were not examined, a recent study examining this issue reported that patients spent on average 29 h preparing for, traveling, having the colonoscopy and recovering from the colonoscopy, equating to $353 in lost time and travel costs [52]. Thus having to undergo a repeat colonoscopy due to an inadequate bowel prep results in over $700 ($353 + $353) in additional indirect patients costs.

In Monte Carlo simulation, the uncertainties in all values were considered simultaneously, and were assumed to possess the probability distributions as identified in the Additional file 3: Appendix S3 and Additional file 4: Appendix S4. This furnishes decision makers with a range of possible outcomes (costs and QALYs) and with the probabilities that will occur for a choice in action. In this Markov model, it was found that outcomes of costs and QALYs centered around a consistent and rather narrow range of values as identified in “Results” section—identifying the use of PureVu as the less costly and similar to/improved QALYs versus SOC.

In sensitivity analysis it was identified in Fig. 4 that in order for SOC to be the less expensive option, compliance would need to be ≤ 28%. In practice the compliance rate has been estimated to be 60% [1]. Therefore compliance lower < 60% is unlikely to occur in every day practice.

Lastly, a similar analysis to this was reported on recently using high volume colonic water irrigation [53]. This analysis identified similar findings to the ones contained herein, namely lower costs and higher QALYs during periprocedural high volume irrigation. The differences in the analyses centered around sensitivity analysis of patient compliance to clinical guidelines (compliance) and associated costs.

Strengths of this analysis are that it takes into account all direct costs including repeat colonoscopies due to inadequate prep. As well, the analysis examines both Medicare and private payer reimbursement rates which were used as proxy for costs. The analysis mirrors current clinical practice related to colonoscopy in the US; including the fact that some patients do not adhere to bowel prep and recommendations for repeat colonoscopies. Additionally, the analysis only focuses on patients where PureVu would benefit most, those ~ 25% of patients with inadequately prepped colons. Limitations of this analysis include the fact that the data on costs and QoL are derived from different sources, which may introduce the potential for confounding. However, it should be noted that the QoL estimates came from those in the age ranges of 60–75 years, male, married and retired [41, 54, 55]. These baseline characteristics are consistent with the types of patients who are diagnosed with colorectal cancer (and from which the reimbursements/costs were derived) [56]. Estimates of facility and professional claims for commercial payment were made using a range of a 1.63–2.48 multiplier of the national average Medicare amount used in the models [35–37]. While an attempt was made to fairly reflect private payer reimbursement amounts by examining various sources, there may have been biases in the data identified based on how the data was sampled and reported on. Thus the findings for private payer may not be representative of the prices paid by the broader privately insured population. Further retrospective data was used in the Markov model. The analysis also did not account for indirect costs—estimated at $350 per colonoscopy. Since PureVu is early in its market introduction and is focused on inpatients only, the analysis is a forward looking analysis on outpatient care. While the assumptions in the model are based on the existing literature, the outcomes (for costs and QALYs) need to be borne out in a prospective clinical evaluation of PureVu. The intention is to undertake prospective studies to evaluate these outcomes.

## Supplementary Information

**Additional file 1: Appendix S1.** CHEERS checklist.

**Additional file 2: Appendix S2.** Life expectancy advanced CRC screen shot.

**Additional file 3: Appendix S3.** Propensity Scores.

**Additional file 4: Appendix S4.** PubMed searches.

**Additional file 5: Appendix S5.** Variables used in model.

**Additional file 6: Appendix S6.** Distributions used in model.

**Additional file 7: Appendix S7.** Equations used in Markov model of standard of care (SOC).

**Additional file 8: Appendix S8.** State and Stage transitions—PureVu.

## Data Availability

For information not contained in the manuscript and appendices, it is available upon request including the TreeAge Pro model.

## Abbreviations

CHEERS: Consolidated Health Economic Evaluation Reporting Standards
CRC: Colorectal cancer
QALY: Quality adjusted life year
QOL: Quality of life
SOC: Standard of care
