# Supplementary material for: Colonoscopy in poorly prepped colons: a cost effectiveness analysis comparing standard of care to a new cleansing technology
Source: Cost Eff Resour Alloc. 2021 Apr 29;19:25. doi: 10.1186/s12962-021-00277-5 (PMC8082895; doi:10.1186/s12962-021-00277-5)

Appendix S2: Using the life expectancy calculator for colorectal cancer as found at: <http://www.lifemath.net/cancer/coloncancer/outcome/index.php>


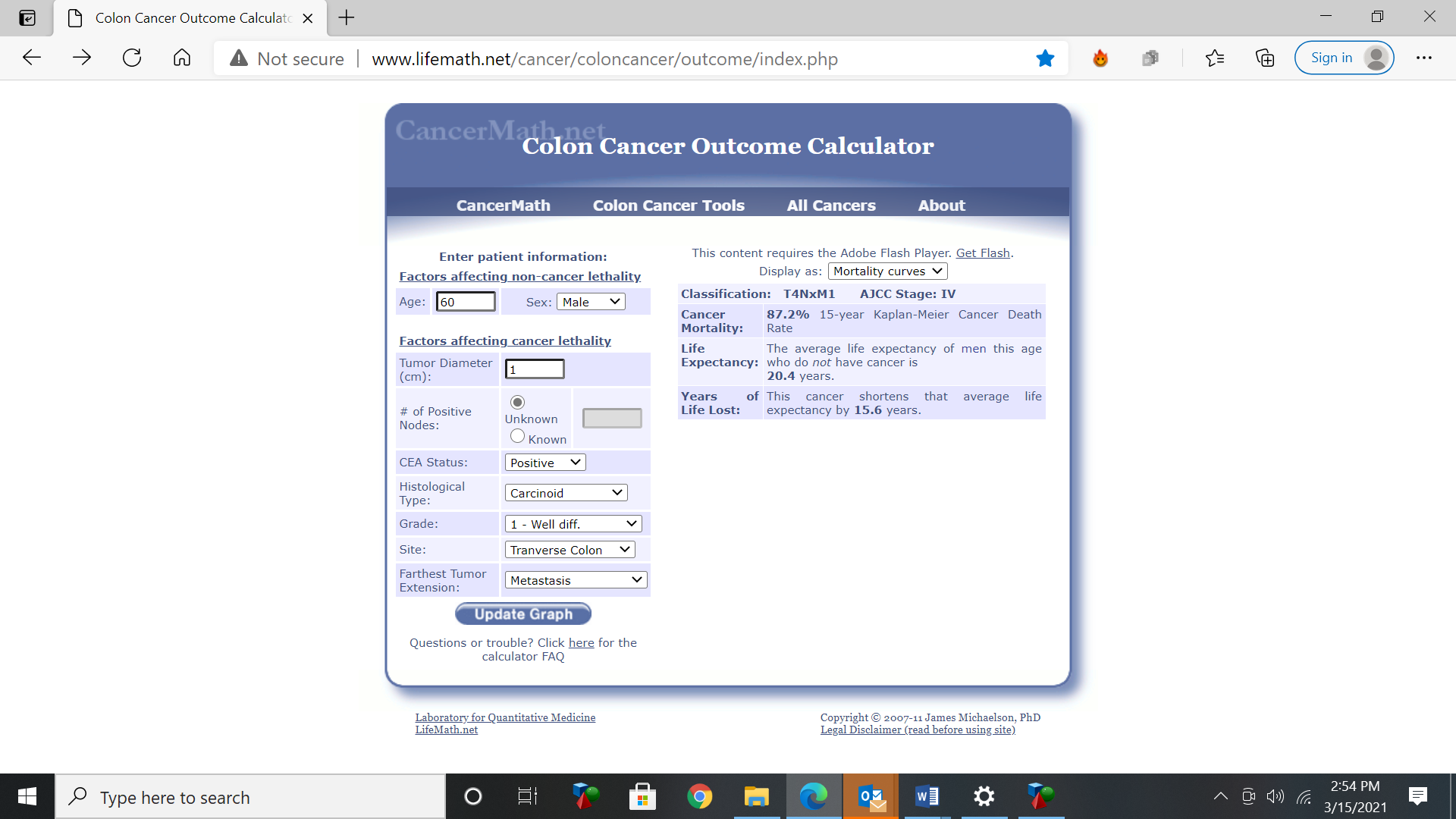

Supplement: Supplementary file 2 — Additional file 2: Appendix S2. Life expectancy advanced CRC screen shot. [file 12962_2021_277_MOESM2_ESM.docx]
