# Supplementary material for: Colonoscopy in poorly prepped colons: a cost effectiveness analysis comparing standard of care to a new cleansing technology
Source: Cost Eff Resour Alloc. 2021 Apr 29;19:25. doi: 10.1186/s12962-021-00277-5 (PMC8082895; doi:10.1186/s12962-021-00277-5)
Supplement: Supplementary file 3 — Additional file 3: Appendix S3. Propensity Scores. [file 12962_2021_277_MOESM3_ESM.docx]

**Appendix S3 propensity score analysis for studies included related to use of PureVu or not in Markov Model**

| **PureVu** | **Bowel prep** | **Age** | **Sex_Male** | **Cecum** | **Samples size** | **Article** | **Propensity score** | **Note** |
| --- | --- | --- | --- | --- | --- | --- | --- | --- |
| 0 | 0.76 | 63.2 | 0.49 | 0.92 | 3,047 | Lebwohl B. The impact of suboptimal preparation on adenoma miss rates and the factors associated with early repeat colonoscopy. Gastrointest Endosc. 2011 | 0.027 | Included |
| 0 | 0.94 | 63.4 | 0.6 | 0.967 | 3,627 | Schoen RE, et al. Utilization of surveillance colonoscopy in community practice. Gastroenterol 2010 | 0.030 | Included |
| 0 | 0.75 | 63.2 | 0.56 | 0.802 | 6,925 | Atkin W, et al. Adeoma surveillance and colorectal cancer incidence: a retrospective, multicentre, cohort study. Lancet Oncol 2017 | 0.030 | Included |
| 0 | 0.98 | 61.7 | 0.44 | 0.98 | 57,588 | Meester RGS et al. Variation in adenoma detection rate and the lifetime benefits and cost of colorectal cancer screening. A microsimulation model. JAMA. 2015 | 0.042 | Included |
| 0 | 0.945 | 61.3 | 0.42 | 0.945 | 20,792 | Corley DA, et al. Variation of adenoma prevalence by age, sex, race, and colon location in a large population: Implications for screening and quality programs. Clin Gastroenterol Hepatol 2013. | 0.047 | Included |
| 1 | 0.191 | 61 | 0.43 | 0.98 | 47 | van Keulen KE, et al. A novel device for intracoronary cleansing of inadequately prepared colonoscopy patients: a feasibility study. Endoscopy 2018 | 0.053 | Included |
| 0 | 0.915 | 62 | 0.988 | 1 | 399 | Miller HL et al. Colonoscopy surveillance after polypectomy may be extended beyond five years. Jrl. Clin Gastroenterol. 2010. | 0.083 | Included |
| 1 | 0.31 | 51.9 | 0.64 | 0.98 | 50 | Jiminez JP, et al. An intraprocedural endoscopic cleasing device for achieving adequate colon preparation in poorly prepped patients. Jrl Clin Gastro. 2019 | 0.664 | Not included in Markov Model |
|  |  |  |  |  |  |  |  |  |
|  | Average | 60.96 | 0.54 |  |  |  |  |  |
|  |  |  |  |  |  |  |  |  |
| **Definitions:** | |  |  |  |  |  |  |  |
| PureVu: 0 no use of PureVu; 1 used PureVu | | | |  |  |  |  |  |
| Bowel prep: 0-1; 0 poor bowel prep; 1 being adequate bowel prep (continuous variable ) | | | | | | |  |  |
| Age: continuous variable | |  |  |  |  |  |  |  |
| Sex: percent of study group being male | | | |  |  |  |  |  |
| Cecum: 0-1; 0 did not reach cecum; 1 successful access to cecum  MedCalc software Ldt. (Version 19.4.0) use of logistic regression with use of logistic regression (LOGREGR_Pred1) predicted values for propensity scores. | | | | | |  |  |  |
