# Supplementary material for: Colonoscopy in poorly prepped colons: a cost effectiveness analysis comparing standard of care to a new cleansing technology
Source: Cost Eff Resour Alloc. 2021 Apr 29;19:25. doi: 10.1186/s12962-021-00277-5 (PMC8082895; doi:10.1186/s12962-021-00277-5)
Supplement: Supplementary file 4 — Additional file 4: Appendix S4. PubMed searches. [file 12962_2021_277_MOESM4_ESM.docx]

Appendix S4: Search terms used PubMed searchers performed on May 10, 2020

Search #1: ((((inadequate[All Fields] AND ("intestines"[MeSH Terms] OR "intestines"[All Fields] OR "bowel"[All Fields])) AND preparation[All Fields]) AND ("colonoscopy"[MeSH Terms] OR "colonoscopy"[All Fields])) AND ("neoplasms"[MeSH Terms] OR "neoplasms"[All Fields] OR "cancer"[All Fields])) AND ("epidemiology"[Subheading] OR "epidemiology"[All Fields] OR "incidence"[All Fields] OR "incidence"[MeSH Terms]) – number of hits 19.

Search #2: ((("colonoscopy"[MeSH Terms] OR "colonoscopy"[All Fields]) AND ("intestines"[MeSH Terms] OR "intestines"[All Fields] OR "bowel"[All Fields])) AND preparation[All Fields]) AND ("costs and cost analysis"[MeSH Terms] OR ("costs"[All Fields] AND "cost"[All Fields] AND "analysis"[All Fields]) OR "costs and cost analysis"[All Fields] OR "costs"[All Fields]) – number of hits 62.
