# Supplementary material for: Colonoscopy in poorly prepped colons: a cost effectiveness analysis comparing standard of care to a new cleansing technology
Source: Cost Eff Resour Alloc. 2021 Apr 29;19:25. doi: 10.1186/s12962-021-00277-5 (PMC8082895; doi:10.1186/s12962-021-00277-5)
Supplement: Supplementary file 5 — Additional file 5: Appendix S5. Variables used in model. [file 12962_2021_277_MOESM5_ESM.docx]

| **NAME** | **DESCRIPTION** | **FORMULA** | **VALUE** | **LOW** | **HIGH** | **COMMENT** |
| --- | --- | --- | --- | --- | --- | --- |
| Compliance_screening_CRC | Compliance with screening for colorectal cancer =>60 year old | 60.300% | 60.300% | 0.000% | 90.000% | Derived from: Colorectal Cancer, Facts & Figures 2017-2019. American Cancer Society |
| Compliance_screening_CRC_PureVu |  | 60.300% | 60.300% | 0.000% | 99.000% | Derived from: Colorectal Cancer, Facts & Figures 2017-2019. American Cancer Society |
| Compliance_surveillance_post_adenoma | Compliance with a surveillance colonoscopy post adenoma (<1cm) | 90.000% | 90.000% | 0.000% | 95.000% | Percent of practicing gastroenterologists who perceive a need for surveillance post non-advanced adenoma removal (<1cm). Schoen, et al. Utilization of surveillance colonoscopy in community practice. Gastroenterology 2010;138(1): 73. doi:10.1053/j.gastro.2009.09.062 Note: this is based on the 2012 AGA recommended guideline for colonoscopy surveillance after polypectomy (non-advanced adenoma) of 5 years. |
| Complication_rate_diagnostic_colonoscopy | Complication rate of diagnostic colonoscopy | Probability_complication_diag_colonoscopy_bleeding+Probability_complication_diag_colonoscopy_perforation | 0.423% | 0.000% | 1.000% |  |
| Complication_rate_polypectomy | Complication rate of polypectomy | Complication_polypectomy_cardiovascular (0.1% – 2.5%) +Complication_polypectomy_other_GI (0.2% - 2.6%) +Complication_polypectomy_serious_GI (0.2% - 2.9%) + Fatal (0.0033%) | 0.5033% | 0.000% | 8.000% | Source: Meester RGS et al. Variation in adenoma detection rate and the lifetime benefits and cost of colorectal cancer screening. A microsimulation model. JAMA 2015; 313(23):2349-2358. Use of Table 1: Key Modeling Assumptions; page 2351. Assumptions for complication rates |
| Comply_payoff |  | (Diagnostic_colonoscopy_CPT45378+Diagnostic_colonoscopy_APC5311+CPT_00810_anesthesia+Complication_rate_diagnostic_colonoscopy*Cost_complications_diag_colonoscopy)*Private_pay_reimbursement_premium | $2,202 | $0 | $2,202 | Medicare reimbursement. National average payment amounts. |
| Cost_complications_diag_colonoscopy | Cost of complications related to diagnostic colonoscopy | $10,425 | $10,425 | $0 | $20,000 | Source: Meester RGS et al. Variation in adenoma detection rate and the lifetime benefits and cost of colorectal cancer screening. A microsimulation model. JAMA 313(23):2349-2358. |
| Cost_complications_post_polypectomy | Cost complications associated with polypectomy | $10,425 | $10,425 | $0 | $20,000 | Source: Meester RGS et al. Variation in adenoma detection rate and the lifetime benefits and cost of colorectal cancer screening. A microsimulation model. JAMA 313(23):2349-2358. |
| Cost_PureVu | Cost PureVu colon cleansing system | $750 | $750 | $0 | $15,000 |  |
| Cost_treatment_last_year_life_colorectal_cancer | Costs treatment colorectal cancer last year of life | $166,670 | $166,670 | $0 | $200,000 | Source: Mariotto AB et al. Projections of the cost of cancer care in the US: 2010-2020. Jrl. Natl Cancer Instit. 2011;103:117-128. Last year costs were inflated to 2017 using the medical CPI for outpatient care. |
| Cost_treatment_ongoing_colorectal_cancer | Costs for ongoing treatment colorectal cancer | $5,960 | $5,960 | $0 | $10,000 | Source: Mariotto AB et al. Projections of the cost of cancer care in the US: 2010-2020. Jrl. Natl Cancer Instit. 2011;103:117-128. Ongoing costs were inflated to 2017 using the medical CPI for outpatient care. |
| Cost_treatment_year1_colorectal_cancer | Cost for treatment of colorectal cancer first year. | $80,640 | $80,640 | $0 | $80,640 | Source: Mariotto AB et al. Projections of the cost of cancer care in the US: 2010-2020. Jrl. Natl Cancer Instit. 2011;103:117-128. Initial costs were inflated to 2017 using the medical CPI for outpatient care. |
| Cost_treatment_year1_early_stage_CRC | Cost treating early stage CRC (stages 0-2) initial | $52,640 | $52,640 | $0 | $100,000 | Source: Lang K, et al. Lifetime and treatment-phase costs associated with colorectal cancer: Evidence from SEER-Medicare data. Clin Gastro Hepatol. 2009;7:198-204. Use of table 3 data. Costs from year 2006 inflated to 2017 using medical CPI for hospital outpatient. |
| Costs_treatment_ongoing_early_stage_CRC | Costs ongoing treatment early stage CRC | $4,425 | $4,425 | $0 | $8,000 | Source: Lang K, et al. Lifetime and treatment-phase costs associated with colorectal cancer: Evidence from SEER-Medicare data. Clin Gastro Hepatol. 2009;7:198-204. Use of table 3 data. Costs from year 2006 inflated to 2017 using medical CPI for hospital outpatient. |
| Costs_treatment_terminal_early_stage_CRC | Costs last year life early stage CRC | $16,840 | $16,840 | $0 | $32,000 | Source: Lang K, et al. Lifetime and treatment-phase costs associated with colorectal cancer: Evidence from SEER-Medicare data. Clin Gastro Hepatol. 2009;7:198-204. Use of table 3 data. Costs from year 2006 inflated to 2017 using medical CPI for hospital outpatient. |
| CPT_00810_anesthesia | Anesthesia for colonoscopy | $154 | $154 | $0 | $300 | Anesthesia calculation: (Base units + time (one unit = 15 minutes) X CF (22.0454). Assume patient out for 30 minutes = 2 units; CPT 00810 = 5 units; 7 units X 22.0454 = $154 |
| Diagnostic_colonoscopy_APC5311 | APC 5311 - facility payment diagnostic colonoscopy | $668 | $668 | $0 | $1,300 | Medicare facility payment for diagnostic colonoscopy - APC 5311 |
| Diagnostic_colonoscopy_APC5312_adenoma | APC 5312 - facility payment for diagnostic colonoscopy with removal adenomas | $875 | $875 | $0 | $1,650 | Medicare 2017 national average payment rate for facility costs |
| Diagnostic_colonoscopy_CPT45378 | CPT 45378 - diagnostic colonoscopy | $195 | $195 | $0 | $400 | 2017 National Average Medicare Payment for diagnostic colonoscopy. Physician payment associated with the facility setting |
| Diagnostic_colonoscopy_CPT45380_adenoma | CPT 45380 - diagnostic colonoscopy with removal of adenoma(s) | $212 | $212 | $0 | $400 | National average 2017 Medicare payment rate for removal of adenomas |
| Inad_bowel_prep_PureVu |  | Colonoscopy_inad_Prep_PureVu | 25.00% | 0.00% | 99.00% | Sidhu S, Geraghty J, Karpha I, et al. Outcomes following an initial unsuccessful colonoscopy: a 5-year complete audit of teaching hospital colonoscopy practice. Presented at 2011 British Society of Gastroenterology Annual General Meeting; March 14-17, 2011; Birmingham, UK |
| Inadequate_bowel_prep_colonoscopy | Reported rate of inadequate bowel prep | Colonoscopy_inadequate_prep | 28.67% | 0.00% | 90.00% | See distribution values: Range 5-60%; most common value of 25% |
| Interval1 | Screen every 10 years - average risk patient | 10 | 10 | 0 | 10 | Source: Lieberman, DA et al. Guidelines for colonoscopy surveillance after screening and polypectomy: A consensus update by the US multi-society task force on colorectal cancer. Gastroenterol. 2012;43:844-857. |
| Interval2 | Screen every 3 years due to advanced nature of disease | 3 | 3 | 0 | 3 | Source: Lieberman, DA et al. Guidelines for colonoscopy surveillance after screening and polypectomy: A consensus update by the US multi-society task force on colorectal cancer. Gastroenterol. 2012;43:844-857. |
| Interval3 | inadequate prep every 2 years | 2 | 2 | 0 | 2 | Source: Lieberman, DA et al. Guidelines for colonoscopy surveillance after screening and polypectomy: A consensus update by the US multi-society task force on colorectal cancer. Gastroenterol. 2012;43:844-857. Sessile serrated polyp <10mm |
| Life_expectancy_healthy_60yr_old |  | 24 | 24 | 0 | 24 | Source: https://www.johnhancockinsurance.com/life/life-expectancy-tool.aspx |
| Prevalence_adenomas_proximal_colon | Prevalence adenomas in proximal colon (ascending and transverse) | 0.486 | 0.486 | 0 | 0.9 | Source: Corley DA, et al. Variation of adenoma prevalence by age, sex, race, and colon location in a large population: Implications for screening and quality programs. Clin Gastroenterol Hepatol 2013;11(2):172-180 |
| Private_pay_rate |  | Private_pay_rate_multiplier | 2.075 | 0 | 2.075 | White C, Whaley C. Prices paid to hospitals by private health plans are high relative to Medicare and vary widely. RAND report 2019. Accessed on Jan 5, 2019 at: https://www.rand.org/pubs/research_reports/RR3033.html |
| Private_pay_reimbursement_premium | Reimbursement premium paid by private payers over Medicare | Private_pay_rate_multiplier | 2.075 | 0 | 2.075 | White C, Whaley C. Prices paid to hospitals by private health plans are high relative to Medicare and vary widely. RAND report 2019. Accessed on Jan 5, 2019 at: https://www.rand.org/pubs/research_reports/RR3033.html |
| Prob_adenoma_cancerous_inadequate_prep | Probability of adenoma being cancerous with inadequate prep | Probability_adenoma_cancerous_inadequate_prep | 2.63% | 0.00% | 5.00% | Atkin W et al. Lancet Oncol. 2017 |
| Probability_adenoma_is_cancerous_adequate_prep | Probability of adenoma being cancerous | 0.00425 | 0.43% | 0.00% | 1.00% | Probability adenoma is cancerous with adequate prep: 0.7*0.3*.0375 = 0.00425 |
| Probability_compliance_post_colonoscopy | Probability compliance post negative colonoscopy | Prob_compliance_post_neg_colonoscopy | 71.00% | 0.00% | 99.00% | Source: Schoen RE, et al. Utilization of surveillance colonoscopy in community practice. Gastroenterol. 2010;138(1) |
| Probability_complication_diag_colonoscopy_bleeding | Probability diagnostic colonoscopy bleeding complication | Complication_colonoscopy_bleeding | 0.31% | 0.00% | 1.00% | Source: Heitman SJ et al. Colorectal cancer screening for average-risk North Amer. PLoS One 2010;7(11):e10000370 |
| Probability_complication_diag_colonoscopy_perforation | Probability perforation complication diagnostic colonoscopy | Complication_colonoscopy_perforation | 0.11% | 0.00% | 1.00% | Source: Heitman SJ et al. Colorectal cancer screening for average-risk North Amer. PLoS One 2010;7(11):e10000370 |
| Probability_detecting_adenoma_screening | Probability of detecting an adenoma with colonoscopy | Probability_adenoma_detection | 30.00% | 0.00% | 1.00% | Kaminski MF, et al. Quality indicators for colonoscopy and the risk of interval cancer. NEJM. 2010;362:1795-1803. |
| Probability_early_vs_late_CRC | Probability of patient having early versus late CRC | 0.85 | 85.00% | 0.00% | 95.00% | Source: Heitman SJ, Clin Gastro Hepatol 2009 |
| Probability_PureVu_usage_inad_prep | Probability of usage of PureVu if poorly prepped colon | Probability_PureVu_usage | 78.00% | 0.00% | 99.00% | Assumption based on current usage trends of PureVu in inadequately prepped colons. |
| Probability_repeat_colonoscopy_after_inad_prep | Probability of a repeat colonoscopy within 3 years due to inadequate prep | 0.559 | 55.90% | 0.00% | 95.00% | Source: Menees SB, et al. The impact of fair colonoscopy preparation on colonoscopy use and adenoma miss rate in patients undergoing outpatient colonoscopy. Gastro Endos 2013;78(3) |
| Probability_TN_adequate_BP | Probability TN colonoscopy with adequate bowel prep | 0.931 | 93.10% | 0.00% | 99.00% | Source: AHRQ 2016; Screening for colorectal cancer: A systematic review for the US Preventive Services Task Force. Table 10; page 126; assumes high end of specificity values for a TN value for an adenoma =>6mm |
| Probability_TP_adequate_BP | Probability true positive finding with adequate bowel prep | 0.9 | 90.00% | 0.00% | 95.00% | Source: AHRQ 2016; Screening for colorectal cancer: A systematic review for the US Preventive Services Task Force. Table 10. page 126 of the report |
| Probabilty_dying_adv_CRC_per_year |  | 0.2 | 20.00% | 0.00% | 40.00% | Source: American Cancer society survival rates advanced CRC. https://www.cancer.org/cancer/colon-rectal-cancer/detection-diagnosis-staging/survival-rates.html |
| PureVu_prob | Use of PureVu | 0.99 | 99.00% | 0.00% | 1.00% |  |
| QoL_colorectal_advanced_cancer_baseline | QoL advanced colorectal cancer - baseline | QoL_baseline_advanced_CRC | 0.674 | 0 | 0.674 | Source: Augestad KM et al. Cost-effectiveness and quality of life in surgeon vs. general practitioner-organized colon cancer surveillance: a randomized controlled trial. BMJ Open. 3:e002391 |
| QoL_colorectal_advanced_cancer_ongoing | QoL advanced colorectal cancer - ongoing care | QoL_progressive_metastatic_CRC | 0.527333333 | 0 | 0.52733 | Source: Augestad KM et al. Cost-effectiveness and quality of life in surgeon vs. general practitioner-organized colon cancer surveillance: a randomized controlled trial. BMJ Open. 3:e002391 |
| QoL_colorectal_advanced_cancer_remission | QoL advanced colorectal cancer - remission | QoL_remission_advanced_CRC | 0.835 | 0 | 0.835 | Source: Augestad KM et al. Cost-effectiveness and quality of life in surgeon vs. general practitioner-organized colon cancer surveillance: a randomized controlled trial. BMJ Open. 3:e002391 |
| QoL_death | QoL associated with death | 0 | 0 | 0 | 0 |  |
| QoL_early_stage_CRC_baseline | QoL colorectal cancer early stage baseline | QoL_baseline_early_stage_CRC | 0.7028 | 0 | 0.7028 | Source: Foster C, et al. Pre-surgery depression and confidence to manage problems predict recovery trajectories of health and wellbeing in the first two years following colorectal cancer: Results from the CREW cohort study. PLoS One. doi:10.1371/journal. pone.0155434 |
| QoL_early_stage_CRC_ongoing | QoL colorectal cancer early stage ongoing | QoL_ongoing_early_stage_CRC | 0.7391 | 0 | 0.7391 | Source: Foster C, et al. Pre-surgery depression and confidence to manage problems predict recovery trajectories of health and wellbeing in the first two years following colorectal cancer: Results from the CREW cohort study. PLoS One. doi:10.1371/journal. pone.0155434 |
| QoL_early_stage_CRC_remission | QoL colorectal cancer early state remission | QoL_remission_early_stage_CRC | 0.7954 | 0 | 0.7954 | Source: Foster C, et al. Pre-surgery depression and confidence to manage problems predict recovery trajectories of health and wellbeing in the first two years following colorectal cancer: Results from the CREW cohort study. PLoS One. doi:10.1371/journal. pone.0155434 |
| QoL_no_CRC | Quality of life no colorectal cancer | 0.91 | 0.91 | 0 | 0.91 | Source: Heitman SJ et al. Colorectal cancer screening for average-risk North Amer. PLoS One 2010;7(11):e10000370 |
| QoL_positive_screening_colonoscopy | QoL with a positive screening colonoscopy | QoL_baseline_positive_screening_colonoscopy | 0.728 | 0 | 0.728 | Source: Kapidzic A, et al. Quality of life in participants of a CRC screening program. BJC 2012;107;1295-1301 |
| QoL_screening_colonoscopy | Screening QoL colonoscopy | 0.9 | 0.9 | 0 | 0.9 | Source: Swan JS, et al. Responsiveness of the testing morbidities index in colonoscopy. Value In Health. 2013;13:1046-1053. |
