# Supplementary material for: Colonoscopy in poorly prepped colons: a cost effectiveness analysis comparing standard of care to a new cleansing technology
Source: Cost Eff Resour Alloc. 2021 Apr 29;19:25. doi: 10.1186/s12962-021-00277-5 (PMC8082895; doi:10.1186/s12962-021-00277-5)
Supplement: Supplementary file 6 — Additional file 6: Appendix S6. Distributions used in model. [file 12962_2021_277_MOESM6_ESM.docx]

**Appendix S6 distributions**

| **NAME** | **DESCRIPTION** | **TYPE** | **PARAMETERS** | **EV** | **COMMENT** |
| --- | --- | --- | --- | --- | --- |
| QoL_progressive_metastatic_CRC | QoL treatment of metastatic CRC | Triangular | min: 0.432, likeliest: 0.527, max: 0.623 | 0.52733 | Best JH et al. Preference values associated with stage III colon cancer and adjuvant chemotherapy. Qual Life Res. 2010;19:391-400. Table 2; page 396 |
| QoL_remission_early_stage_CRC | QoL remission early stage CRC | Normal | mean: 0.7954, stddev: 0.1607 | 0.7954 | Source: Foster C, et al. Pre-surgery depression and confidence to manage problems predict recovery trajectories of health and wellbeing in the first two years following colorectal cancer: Results from the CREW cohort study. PLoS One. doi:10.1371/journal. pone.0155434 |
| Colonoscopy_inad_Prep_PureVu | Use of PureVu when there is an inadequate prep of the colon | Normal | mean: 0.25, stddev: 0.01, overridden Mean: 0.25 | 0.25 | 2012 findings of inadequate bowel prep |
| Complication_polypectomy_other_GI | Probability other GI event - polypectomy | Uniform | subtype: 2, low: 0.00002, high: 0.0026 | 0.00131 | Source: Meester RGS et al. Variation in adenoma detection rate and the lifetime benefits and cost of colorectal cancer screening. A microsimulation model. JAMA 313(23):2349-2358. |
| Probability_adenoma_detection | Probability of adenoma detection | Uniform | subtype: 2, low: 0.0, high: 0.6 | 0.3 | Kaminski MF, et al. Quality indicators for colonoscopy and the risk of interval cancer. NEJM. 2010;362:1795-1803. |
| Complication_polypectomy_cardiovascular | Probability cardiovascular event - polypectomy | Uniform | subtype: 2, low: 0.00001, high: 0.0025 | 0.001255 | Source: Meester RGS et al. Variation in adenoma detection rate and the lifetime benefits and cost of colorectal cancer screening. A microsimulation model. JAMA 313(23):2349-2358. |
| Complication_colonoscopy_perforation | Risk perforation complication resulting from diagnostic colonoscopy | Triangular | min: 0.0005, likeliest: 0.0009, max: 0.002 | 0.0011333 | Source: Heitman SJ et al. Colorectal cancer screening for average-risk North Amer. PLoS One 2010;7(11):e10000370 |
| Prob_compliance_post_neg_colonoscopy | Probability compliance with surveillance post negative colonoscopy | Triangular | min: 0.57, likeliest: 0.66, max: 0.9 | 0.71 | Source: Schoen RE et al. Utilization of surveillance colonoscopy in community practice Gastroenterol. 2010;138(1):73.. doi:10.1053/j.gastro.2009.09.062. |
| QoL_remission_advanced_CRC | QoL remission CRC | Normal | mean: 0.835, stddev: 0.148 | 0.835 | Source: Augestad KM et al. Cost-effectiveness and quality of life in surgeon vs. general practitioner-organized colon cancer surveillance: a randomized controlled trial. BMJ Open. 3:e002391 |
| QoL_baseline_early_stage_CRC | QoL baseline for early stage CRC | Normal | mean: 0.7028, stddev: 0.22 | 0.7028 | Source: Foster C, et al. Pre-surgery depression and confidence to manage problems predict recovery trajectories of health and wellbeing in the first two years following colorectal cancer: Results from the CREW cohort study. PLoS One. doi:10.1371/journal. pone.0155434 |
| QoL_baseline_positive_screening_colonoscopy | Positive test adenoma screening colonoscopy | Normal | mean: 0.728, stddev: 0.186 | 0.728 | Source: Kapidzic A, et al. Quality of life in participants of a CRC screening program. BJC 2012;107;1295-1301 |
| Probability_PureVu_usage | Probability PureVu usage with a poorly prepped colon | Uniform | subtype: 2, low: 0.56, high: 1. | 0.78 |  |
| QoL_baseline_advanced_CRC | Quality of life baseline - CRC | Normal | mean: 0.674, stddev: 0.174 | 0.674 | Source: Augestad KM et al. Cost-effectiveness and quality of life in surgeon vs. general practitioner-organized colon cancer surveillance: a randomized controlled trial. BMJ Open. 3:e002391 |
| Private_pay_rate_multiplier | Private pay rate multiplier vs. Medicare rate | Uniform | subtype: 2, low: 1.67, high: 2.48 | 2.075 |  |
| Probability_adenoma_cancerous_non_compliant_HR | Probability of adenoma being cancerous in a noncompliant patient | Triangular | min: 0.0013, likeliest: 0.208, max: 0.325 | 0.1781 | Heitman et al. Clin Gastro Hepatol 2009; \Cancer rate in an average risk 50 year old male is 5.9% over the life of the patient. Therefore 0.43*0.137 =0.059. However, in a high risk individual, the risk is 30% greater. Therefore, likeliest value of 0.16 for average risk is 1.3 times higher for high risk or: 0.16 X 1.3 = 0.208. Schoen RE, et al. Incidence and mortality of colorectal cancer in individuals with a family history of colorectal cancer. Gastroenterol 2015;149(6): 1438-1445. |
| Probability_adenoma_cancerous_inadequate_prep | Probability of adenoma being cancerous with inadequate prep | Triangular | min: 0.005, likeliest: 0.024, max: 0.05 | 0.026333 | Atkin W, et al. Adenoma surveillance and colorectal cancer incidence: a retrospective, multicentre, cohort study. Lancet Oncol. 2017 |
| Colonoscopy_inadequate_prep | Inadequate bowel preparation in colonoscopy | Triangular | min: 0.01, likeliest: 0.25, max: 0.6 | 0.28667 | Values for inadequate bowel prep derived from Kingsley et al. Cost effectiveness of screening colonoscopy depends on adequate bowel preparation rates - a modeling study. PLOS One. 2016;11(12): e0167452. doi:10.1371/journal.pone.0167452. Inadequate bowel prep defined as fair to poor bowel preparation. This derived from the American Society Gastrointestinal Endoscopy (ASGE)/American Gastrointestinal Association (AGA) taskforce on quality in endoscopy. |
| QoL_ongoing_early_stage_CRC | QoL ongoing early stage CRC | Normal | mean: 0.7391, stddev: 0.1729 | 0.7391 | Source: Foster C, et al. Pre-surgery depression and confidence to manage problems predict recovery trajectories of health and wellbeing in the first two years following colorectal cancer: Results from the CREW cohort study. PLoS One. doi:10.1371/journal. pone.0155434 |
| Complication_colonoscopy_bleeding | Bleeding complication resulting from diagnostic colonoscopy | Triangular | min: 0.0, likeliest: 0.0003, max: 0.009 | 0.0031 | Source: Heitman SJ et al. Colorectal cancer screening for average-risk North Amer. PLoS One 2010;7(11):e10000370 |
| Complication_polypectomy_serious_GI | Probability serious GI event polypectomy | Uniform | subtype: 2, low: 0.00002, high: 0.0029 | 0.00146 | Source: Meester RGS et al. Variation in adenoma detection rate and the lifetime benefits and cost of colorectal cancer screening. A microsimulation model. JAMA 313(23):2349-2358. |
| Adenoma_detection_rate_noncompliance | non compliant detection rate target in asymptomatic, average risk individuals | Triangular | min: 0.2, likeliest: 0.5, max: 0.6 | 0.43333 | Source: Rex et al. Quality indicators for colonoscopy. Gastrointestinal Endoscopy. 2015; 81(1):31-49 |
