# Supplementary material for: Colonoscopy in poorly prepped colons: a cost effectiveness analysis comparing standard of care to a new cleansing technology
Source: Cost Eff Resour Alloc. 2021 Apr 29;19:25. doi: 10.1186/s12962-021-00277-5 (PMC8082895; doi:10.1186/s12962-021-00277-5)
Supplement: Supplementary file 7 — Additional file 7: Appendix S7. Equations used in Markov model of standard of care (SOC). [file 12962_2021_277_MOESM7_ESM.docx]

**Appendix S7 – equations used in Markov Model Standard of Care**

| **Health State** | **Equation used** | **Cost disc.** | **QALY disc** | **Prob.** |
| --- | --- | --- | --- | --- |
| **Comply** |  | $3,041.00 | 8.76 | 59% |
| Initial cost | If(Mod(_stage;Interval1)=0;Diagnostic_colonoscopy_CPT45378+Diagnostic_colonoscopy_APC5311+CPT_00810_anesthesia+Complication_rate_diagnostic_colonoscopy*Cost_complications_diag_colonoscopy;0) |  |  |  |
| Incremental cost | If(Mod(_stage;Interval1)=0;Diagnostic_colonoscopy_CPT45378+Diagnostic_colonoscopy_APC5311+CPT_00810_anesthesia+Complication_rate_diagnostic_colonoscopy*Cost_complications_diag_colonoscopy;0) |  |  |  |
| Final cost | If(Mod(_stage;Interval1)=0;Diagnostic_colonoscopy_CPT45378+Diagnostic_colonoscopy_APC5311+CPT_00810_anesthesia+Complication_rate_diagnostic_colonoscopy*Cost_complications_diag_colonoscopy;0) |  |  |  |
| **Early CRC** |  | $2,397.00 | 0.37 | 3% |
| Initial cost | Cost_treatment_year1_early_stage_CRC |  |  |  |
| Incremental cost | Costs_treatment_ongoing_early_stage_CRC |  |  |  |
| Final cost | Costs_treatment_terminal_early_stage_CRC |  |  |  |
| **Advanced CRC** |  | $158.00 | 0.1 | 0.9% |
| Initial cost | Cost_treatment_year1_colorectal_cancer |  |  |  |
| Incremental cost | Cost_treatment_ongoing_colorectal_cancer |  |  |  |
| Final cost | Cost_treatment_last_year_life_colorectal_cancer |  |  |  |
| **Redo colonscopy due to inadequate prep** |  | $1,214.00 | 0.2 | 0.0% |
| Initial cost | Diagnostic_colonoscopy_CPT45378+Diagnostic_colonoscopy_APC5311+CPT_00810_anesthesia+Diagnostic_colonoscopy_CPT45380_adenoma+Diagnostic_colonoscopy_APC5311+CPT_00810_anesthesia+Complication_rate_diagnostic_colonoscopy*Cost_complications_diag_colonoscopy |  |  |  |
| Incremental cost | Diagnostic_colonoscopy_CPT45378+Diagnostic_colonoscopy_APC5311+CPT_00810_anesthesia+Diagnostic_colonoscopy_CPT45380_adenoma+Diagnostic_colonoscopy_APC5311+CPT_00810_anesthesia+Complication_rate_diagnostic_colonoscopy*Cost_complications_diag_colonoscopy |  |  |  |
| Final cost | Diagnostic_colonoscopy_CPT45378+Diagnostic_colonoscopy_APC5311+CPT_00810_anesthesia+Diagnostic_colonoscopy_CPT45380_adenoma+Diagnostic_colonoscopy_APC5311+CPT_00810_anesthesia+Complication_rate_diagnostic_colonoscopy*Cost_complications_diag_colonoscopy |  |  |  |
| **Adenoma surveillance** |  | $1,529.00 | 0.29 | 0.0% |
| Initial cost | Diagnostic_colonoscopy_APC5312_adenoma+Diagnostic_colonoscopy_CPT45380_adenoma+CPT_00810_anesthesia+Complication_rate_polypectomy*Cost_complications_post_polypectomy |  |  |  |
| Incremental cost | Diagnostic_colonoscopy_APC5312_adenoma+Diagnostic_colonoscopy_CPT45380_adenoma+CPT_00810_anesthesia+Complication_rate_polypectomy*Cost_complications_post_polypectomy |  |  |  |
| Final cost | Diagnostic_colonoscopy_APC5312_adenoma+Diagnostic_colonoscopy_CPT45380_adenoma+CPT_00810_anesthesia+Complication_rate_polypectomy*Cost_complications_post_polypectomy |  |  |  |
| Die |  | $815 | 5.48 | 37% |
|  |  |  |  |  |
| Sum |  | $9,154.00 | 15.10 | 100.0% |
