# Supplementary material for: Colonoscopy in poorly prepped colons: a cost effectiveness analysis comparing standard of care to a new cleansing technology
Source: Cost Eff Resour Alloc. 2021 Apr 29;19:25. doi: 10.1186/s12962-021-00277-5 (PMC8082895; doi:10.1186/s12962-021-00277-5)
Supplement: Supplementary file 8 — Additional file 8: Appendix S8. State and Stage transitions—PureVu. [file 12962_2021_277_MOESM8_ESM.docx]

**Appendix S8 state and state transitions PureVu**

| **STAGE** | **NODENAME** | **PROBABILITY** | **COSTDISC** | **COST** | **STAGECOST** | **TOTALCOST** | **EFFECTIVENESS DISC** | **EFFECTIVENESS** | **TOTAL EFFECTIVENESS** |
| --- | --- | --- | --- | --- | --- | --- | --- | --- | --- |
| 0 | comply | 60.300% | $2,202.33 | $1,328.00 | $1,328.00 | $1,328.00 | 0.90 | 0.54 | 0.54 |
| 0 | Non-comply post colonoscopy | 0.000% | $0.00 | $0.00 | $1,328.00 | $1,328.00 | 0.00 | 0.00 | 0.54 |
| 0 | Early CRC | 0.000% | $52,640.00 | $0.00 | $1,328.00 | $1,328.00 | 0.70 | 0.00 | 0.54 |
| 0 | Advanced CRC | 0.000% | $80,640.00 | $0.00 | $1,328.00 | $1,328.00 | 0.67 | 0.00 | 0.54 |
| 0 | Die other causes | 0.000% | $0.00 | $0.00 | $1,328.00 | $1,328.00 | 0.00 | 0.00 | 0.54 |
| 0 | Colonoscopy with PureVu due added to inadequate prep or not comply | 0.000% | $750.00 | $0.00 | $1,328.00 | $1,328.00 | 0.73 | 0.00 | 0.54 |
| 0 | Adenoma surveillance | 0.000% | $3,051.21 | $0.00 | $1,328.00 | $1,328.00 | 0.73 | 0.00 | 0.54 |
| 0 | Non-compliance with system | 39.700% | $0.00 | $0.00 | $1,328.00 | $1,328.00 | 0.00 | 0.00 | 0.54 |
| 0 | Dead | 0.000% | $80,640.00 | $0.00 | $1,328.00 | $1,328.00 | 0.00 | 0.00 | 0.54 |
| 0 | Screening every 10 years - average risk | 60.300% | $0.00 | $0.00 | $1,328.00 | $1,328.00 | 0.00 | 0.00 | 0.54 |
| 0 | Adequate Prep | 43.014% | $0.00 | $0.00 | $1,328.00 | $1,328.00 | 0.00 | 0.00 | 0.54 |
| 0 | no adenoma screening | 30.110% | $0.00 | $0.00 | $1,328.00 | $1,328.00 | 0.00 | 0.00 | 0.54 |
| 0 | Colonoscopy TN | 28.032% | $0.00 | $0.00 | $1,328.00 | $1,328.00 | 0.00 | 0.00 | 0.54 |
| 0 | Comply | 16.903% | $0.00 | $0.00 | $1,328.00 | $1,328.00 | 0.00 | 0.00 | 0.54 |
| 0 | Non-comply | 11.129% | $0.00 | $0.00 | $1,328.00 | $1,328.00 | 0.00 | 0.00 | 0.54 |
| 0 | Colonoscopy FP | 2.078% | $0.00 | $0.00 | $1,328.00 | $1,328.00 | 0.00 | 0.00 | 0.54 |
| 0 | adenoma | 12.904% | $0.00 | $0.00 | $1,328.00 | $1,328.00 | 0.00 | 0.00 | 0.54 |
| 0 | Distal Colon (includes descending, sigmoid colon, splenic flexure, rectum) | 6.633% | $0.00 | $0.00 | $1,328.00 | $1,328.00 | 0.00 | 0.00 | 0.54 |
| 0 | Colonoscopy TP | 5.969% | $0.00 | $0.00 | $1,328.00 | $1,328.00 | 0.00 | 0.00 | 0.54 |
| 0 | Colonoscopy FN | 0.663% | $0.00 | $0.00 | $1,328.00 | $1,328.00 | 0.00 | 0.00 | 0.54 |
| 0 | cancer | 0.003% | $0.00 | $0.00 | $1,328.00 | $1,328.00 | 0.00 | 0.00 | 0.54 |
| 0 | early | 0.002% | $0.00 | $0.00 | $1,328.00 | $1,328.00 | 0.00 | 0.00 | 0.54 |
| 0 | late | 0.000% | $0.00 | $0.00 | $1,328.00 | $1,328.00 | 0.00 | 0.00 | 0.54 |
| 0 | no cancer | 0.660% | $0.00 | $0.00 | $1,328.00 | $1,328.00 | 0.00 | 0.00 | 0.54 |
| 0 | Comply | 0.398% | $0.00 | $0.00 | $1,328.00 | $1,328.00 | 0.00 | 0.00 | 0.54 |
| 0 | Non-comply | 0.262% | $0.00 | $0.00 | $1,328.00 | $1,328.00 | 0.00 | 0.00 | 0.54 |
| 0 | Proximal colon (includes ascending, transverse) | 6.271% | $0.00 | $0.00 | $1,328.00 | $1,328.00 | 0.00 | 0.00 | 0.54 |
| 0 | Colonoscopy TP | 5.644% | $0.00 | $0.00 | $1,328.00 | $1,328.00 | 0.00 | 0.00 | 0.54 |
| 0 | Colonoscopy FN | 0.627% | $0.00 | $0.00 | $1,328.00 | $1,328.00 | 0.00 | 0.00 | 0.54 |
| 0 | cancer | 0.003% | $0.00 | $0.00 | $1,328.00 | $1,328.00 | 0.00 | 0.00 | 0.54 |
| 0 | early | 0.002% | $0.00 | $0.00 | $1,328.00 | $1,328.00 | 0.00 | 0.00 | 0.54 |
| 0 | late | 0.000% | $0.00 | $0.00 | $1,328.00 | $1,328.00 | 0.00 | 0.00 | 0.54 |
| 0 | no cancer | 0.624% | $0.00 | $0.00 | $1,328.00 | $1,328.00 | 0.00 | 0.00 | 0.54 |
| 0 | Comply | 0.377% | $0.00 | $0.00 | $1,328.00 | $1,328.00 | 0.00 | 0.00 | 0.54 |
| 0 | Non-comply | 0.248% | $0.00 | $0.00 | $1,328.00 | $1,328.00 | 0.00 | 0.00 | 0.54 |
| 0 | Inadequate Prep | 17.286% | $0.00 | $0.00 | $1,328.00 | $1,328.00 | 0.00 | 0.00 | 0.54 |
| 0 | PureVu usage | 13.483% | $0.00 | $0.00 | $1,328.00 | $1,328.00 | 0.00 | 0.00 | 0.54 |
| 0 | Colonoscopy FN | 3.803% | $0.00 | $0.00 | $1,328.00 | $1,328.00 | 0.00 | 0.00 | 0.54 |
| 0 | cancer | 0.100% | $0.00 | $0.00 | $1,328.00 | $1,328.00 | 0.00 | 0.00 | 0.54 |
| 0 | early | 0.085% | $0.00 | $0.00 | $1,328.00 | $1,328.00 | 0.00 | 0.00 | 0.54 |
| 0 | late | 0.015% | $0.00 | $0.00 | $1,328.00 | $1,328.00 | 0.00 | 0.00 | 0.54 |
| 0 | no cancer | 3.703% | $0.00 | $0.00 | $1,328.00 | $1,328.00 | 0.00 | 0.00 | 0.54 |
| 0 | Comply | 2.233% | $0.00 | $0.00 | $1,328.00 | $1,328.00 | 0.00 | 0.00 | 0.54 |
| 0 | Non-comply | 1.470% | $0.00 | $0.00 | $1,328.00 | $1,328.00 | 0.00 | 0.00 | 0.54 |
| 0 | No screening | 0.000% | $0.00 | $0.00 | $1,328.00 | $1,328.00 | 0.00 | 0.00 | 0.54 |
| 0 | Adenoma | 0.000% | $0.00 | $0.00 | $1,328.00 | $1,328.00 | 0.00 | 0.00 | 0.54 |
| 0 | Cancerous | 0.000% | $0.00 | $0.00 | $1,328.00 | $1,328.00 | 0.00 | 0.00 | 0.54 |
| 0 | Early | 0.000% | $0.00 | $0.00 | $1,328.00 | $1,328.00 | 0.00 | 0.00 | 0.54 |
| 0 | Advanced | 0.000% | $0.00 | $0.00 | $1,328.00 | $1,328.00 | 0.00 | 0.00 | 0.54 |
| 0 | Noncancerous | 0.000% | $0.00 | $0.00 | $1,328.00 | $1,328.00 | 0.00 | 0.00 | 0.54 |
| 0 | comply | 0.000% | $0.00 | $0.00 | $1,328.00 | $1,328.00 | 0.00 | 0.00 | 0.54 |
| 0 | Not comply | 0.000% | $0.00 | $0.00 | $1,328.00 | $1,328.00 | 0.00 | 0.00 | 0.54 |
| 0 | No adenoma | 0.000% | $0.00 | $0.00 | $1,328.00 | $1,328.00 | 0.00 | 0.00 | 0.54 |
| 0 | comply | 0.000% | $0.00 | $0.00 | $1,328.00 | $1,328.00 | 0.00 | 0.00 | 0.54 |
| 0 | Not comply | 0.000% | $0.00 | $0.00 | $1,328.00 | $1,328.00 | 0.00 | 0.00 | 0.54 |
| 0 | Continue | 0.000% | $0.00 | $0.00 | $1,328.00 | $1,328.00 | 0.00 | 0.00 | 0.54 |
| 0 | Die | 0.000% | $0.00 | $0.00 | $1,328.00 | $1,328.00 | 0.00 | 0.00 | 0.54 |
| 0 | PureVu usage | 0.000% | $0.00 | $0.00 | $1,328.00 | $1,328.00 | 0.00 | 0.00 | 0.54 |
| 0 | Later followup | 0.000% | $0.00 | $0.00 | $1,328.00 | $1,328.00 | 0.00 | 0.00 | 0.54 |
| 0 | Redo 2 years | 0.000% | $0.00 | $0.00 | $1,328.00 | $1,328.00 | 0.00 | 0.00 | 0.54 |
| 0 | comply | 0.000% | $0.00 | $0.00 | $1,328.00 | $1,328.00 | 0.00 | 0.00 | 0.54 |
| 0 | Not comply | 0.000% | $0.00 | $0.00 | $1,328.00 | $1,328.00 | 0.00 | 0.00 | 0.54 |
| 0 | No redo | 0.000% | $0.00 | $0.00 | $1,328.00 | $1,328.00 | 0.00 | 0.00 | 0.54 |
| 0 | Redo in at least 3 years | 0.000% | $0.00 | $0.00 | $1,328.00 | $1,328.00 | 0.00 | 0.00 | 0.54 |
| 0 | comply | 0.000% | $0.00 | $0.00 | $1,328.00 | $1,328.00 | 0.00 | 0.00 | 0.54 |
| 0 | Not comply | 0.000% | $0.00 | $0.00 | $1,328.00 | $1,328.00 | 0.00 | 0.00 | 0.54 |
| 0 | No redo | 0.000% | $0.00 | $0.00 | $1,328.00 | $1,328.00 | 0.00 | 0.00 | 0.54 |
| 0 | Adenoma | 17.203% | $0.00 | $0.00 | $1,328.00 | $1,328.00 | 0.00 | 0.00 | 0.54 |
| 0 | Cancerous | 3.064% | $0.00 | $0.00 | $1,328.00 | $1,328.00 | 0.00 | 0.00 | 0.54 |
| 0 | Early | 2.604% | $0.00 | $0.00 | $1,328.00 | $1,328.00 | 0.00 | 0.00 | 0.54 |
| 0 | Advanced | 0.460% | $0.00 | $0.00 | $1,328.00 | $1,328.00 | 0.00 | 0.00 | 0.54 |
| 0 | Noncancerous | 14.139% | $0.00 | $0.00 | $1,328.00 | $1,328.00 | 0.00 | 0.00 | 0.54 |
| 0 | No adenoma | 22.497% | $0.00 | $0.00 | $1,328.00 | $1,328.00 | 0.00 | 0.00 | 0.54 |
| 1 | comply | 19.911% | $0.00 | $0.00 | $647.01 | $1,975.02 | 0.87 | 0.17 | 1.30 |
| 1 | Non-comply post colonoscopy | 13.109% | $0.00 | $0.00 | $647.01 | $1,975.02 | 0.00 | 0.00 | 1.30 |
| 1 | Early CRC | 2.694% | $4,296.12 | $115.74 | $647.01 | $1,975.02 | 0.72 | 0.02 | 1.30 |
| 1 | Advanced CRC | 0.475% | $5,786.41 | $27.51 | $647.01 | $1,975.02 | 0.51 | 0.00 | 1.30 |
| 1 | Die other causes | 36.636% | $0.00 | $0.00 | $647.01 | $1,975.02 | 0.88 | 0.32 | 1.30 |
| 1 | Colonoscopy with PureVu due added to inadequate prep or not comply | 13.483% | $728.16 | $98.18 | $647.01 | $1,975.02 | 0.88 | 0.12 | 1.30 |
| 1 | Adenoma surveillance | 13.691% | $2,962.34 | $405.58 | $647.01 | $1,975.02 | 0.88 | 0.12 | 1.30 |
| 1 | Non-compliance with system | 0.000% | $0.00 | $0.00 | $647.01 | $1,975.02 | 0.00 | 0.00 | 1.30 |
| 1 | Dead | 0.000% | $5,786.41 | $0.00 | $647.01 | $1,975.02 | 0.00 | 0.00 | 1.30 |
| 1 | Screening every 10 years - average risk | 0.000% | $0.00 | $0.00 | $647.01 | $1,975.02 | 0.00 | 0.00 | 1.30 |
| 1 | Adequate Prep | 0.000% | $0.00 | $0.00 | $647.01 | $1,975.02 | 0.00 | 0.00 | 1.30 |
| 1 | no adenoma screening | 0.000% | $0.00 | $0.00 | $647.01 | $1,975.02 | 0.00 | 0.00 | 1.30 |
| 1 | Colonoscopy TN | 0.000% | $0.00 | $0.00 | $647.01 | $1,975.02 | 0.00 | 0.00 | 1.30 |
| 1 | Comply | 0.000% | $0.00 | $0.00 | $647.01 | $1,975.02 | 0.00 | 0.00 | 1.30 |
| 1 | Non-comply | 0.000% | $0.00 | $0.00 | $647.01 | $1,975.02 | 0.00 | 0.00 | 1.30 |
| 1 | Colonoscopy FP | 0.000% | $0.00 | $0.00 | $647.01 | $1,975.02 | 0.00 | 0.00 | 1.30 |
| 1 | adenoma | 0.000% | $0.00 | $0.00 | $647.01 | $1,975.02 | 0.00 | 0.00 | 1.30 |
| 1 | Distal Colon (includes descending, sigmoid colon, splenic flexure, rectum) | 0.000% | $0.00 | $0.00 | $647.01 | $1,975.02 | 0.00 | 0.00 | 1.30 |
| 1 | Colonoscopy TP | 0.000% | $0.00 | $0.00 | $647.01 | $1,975.02 | 0.00 | 0.00 | 1.30 |
| 1 | Colonoscopy FN | 0.000% | $0.00 | $0.00 | $647.01 | $1,975.02 | 0.00 | 0.00 | 1.30 |
| 1 | cancer | 0.000% | $0.00 | $0.00 | $647.01 | $1,975.02 | 0.00 | 0.00 | 1.30 |
| 1 | early | 0.000% | $0.00 | $0.00 | $647.01 | $1,975.02 | 0.00 | 0.00 | 1.30 |
| 1 | late | 0.000% | $0.00 | $0.00 | $647.01 | $1,975.02 | 0.00 | 0.00 | 1.30 |
| 1 | no cancer | 0.000% | $0.00 | $0.00 | $647.01 | $1,975.02 | 0.00 | 0.00 | 1.30 |
| 1 | Comply | 0.000% | $0.00 | $0.00 | $647.01 | $1,975.02 | 0.00 | 0.00 | 1.30 |
| 1 | Non-comply | 0.000% | $0.00 | $0.00 | $647.01 | $1,975.02 | 0.00 | 0.00 | 1.30 |
| 1 | Proximal colon (includes ascending, transverse) | 0.000% | $0.00 | $0.00 | $647.01 | $1,975.02 | 0.00 | 0.00 | 1.30 |
| 1 | Colonoscopy TP | 0.000% | $0.00 | $0.00 | $647.01 | $1,975.02 | 0.00 | 0.00 | 1.30 |
| 1 | Colonoscopy FN | 0.000% | $0.00 | $0.00 | $647.01 | $1,975.02 | 0.00 | 0.00 | 1.30 |
| 1 | cancer | 0.000% | $0.00 | $0.00 | $647.01 | $1,975.02 | 0.00 | 0.00 | 1.30 |
| 1 | early | 0.000% | $0.00 | $0.00 | $647.01 | $1,975.02 | 0.00 | 0.00 | 1.30 |
| 1 | late | 0.000% | $0.00 | $0.00 | $647.01 | $1,975.02 | 0.00 | 0.00 | 1.30 |
| 1 | no cancer | 0.000% | $0.00 | $0.00 | $647.01 | $1,975.02 | 0.00 | 0.00 | 1.30 |
| 1 | Comply | 0.000% | $0.00 | $0.00 | $647.01 | $1,975.02 | 0.00 | 0.00 | 1.30 |
| 1 | Non-comply | 0.000% | $0.00 | $0.00 | $647.01 | $1,975.02 | 0.00 | 0.00 | 1.30 |
| 1 | Inadequate Prep | 0.000% | $0.00 | $0.00 | $647.01 | $1,975.02 | 0.00 | 0.00 | 1.30 |
| 1 | PureVu usage | 0.000% | $0.00 | $0.00 | $647.01 | $1,975.02 | 0.00 | 0.00 | 1.30 |
| 1 | Colonoscopy FN | 0.000% | $0.00 | $0.00 | $647.01 | $1,975.02 | 0.00 | 0.00 | 1.30 |
| 1 | cancer | 0.000% | $0.00 | $0.00 | $647.01 | $1,975.02 | 0.00 | 0.00 | 1.30 |
| 1 | early | 0.000% | $0.00 | $0.00 | $647.01 | $1,975.02 | 0.00 | 0.00 | 1.30 |
| 1 | late | 0.000% | $0.00 | $0.00 | $647.01 | $1,975.02 | 0.00 | 0.00 | 1.30 |
| 1 | no cancer | 0.000% | $0.00 | $0.00 | $647.01 | $1,975.02 | 0.00 | 0.00 | 1.30 |
| 1 | Comply | 0.000% | $0.00 | $0.00 | $647.01 | $1,975.02 | 0.00 | 0.00 | 1.30 |
| 1 | Non-comply | 0.000% | $0.00 | $0.00 | $647.01 | $1,975.02 | 0.00 | 0.00 | 1.30 |
| 1 | No screening | 19.911% | $0.00 | $0.00 | $647.01 | $1,975.02 | 0.00 | 0.00 | 1.30 |
| 1 | Adenoma | 5.681% | $0.00 | $0.00 | $647.01 | $1,975.02 | 0.00 | 0.00 | 1.30 |
| 1 | Cancerous | 0.024% | $0.00 | $0.00 | $647.01 | $1,975.02 | 0.00 | 0.00 | 1.30 |
| 1 | Early | 0.021% | $0.00 | $0.00 | $647.01 | $1,975.02 | 0.00 | 0.00 | 1.30 |
| 1 | Advanced | 0.004% | $0.00 | $0.00 | $647.01 | $1,975.02 | 0.00 | 0.00 | 1.30 |
| 1 | Noncancerous | 5.656% | $0.00 | $0.00 | $647.01 | $1,975.02 | 0.00 | 0.00 | 1.30 |
| 1 | comply | 4.016% | $0.00 | $0.00 | $647.01 | $1,975.02 | 0.00 | 0.00 | 1.30 |
| 1 | Not comply | 1.640% | $0.00 | $0.00 | $647.01 | $1,975.02 | 0.00 | 0.00 | 1.30 |
| 1 | No adenoma | 7.428% | $0.00 | $0.00 | $647.01 | $1,975.02 | 0.00 | 0.00 | 1.30 |
| 1 | comply | 4.479% | $0.00 | $0.00 | $647.01 | $1,975.02 | 0.00 | 0.00 | 1.30 |
| 1 | Not comply | 2.949% | $0.00 | $0.00 | $647.01 | $1,975.02 | 0.00 | 0.00 | 1.30 |
| 1 | Continue | 0.380% | $0.00 | $0.00 | $647.01 | $1,975.02 | 0.00 | 0.00 | 1.30 |
| 1 | Die | 0.095% | $0.00 | $0.00 | $647.01 | $1,975.02 | 0.00 | 0.00 | 1.30 |
| 1 | PureVu usage | 10.517% | $0.00 | $0.00 | $647.01 | $1,975.02 | 0.00 | 0.00 | 1.30 |
| 1 | Later followup | 2.966% | $0.00 | $0.00 | $647.01 | $1,975.02 | 0.00 | 0.00 | 1.30 |
| 1 | Redo 2 years | 0.000% | $0.00 | $0.00 | $647.01 | $1,975.02 | 0.00 | 0.00 | 1.30 |
| 1 | comply | 0.000% | $0.00 | $0.00 | $647.01 | $1,975.02 | 0.00 | 0.00 | 1.30 |
| 1 | Not comply | 0.000% | $0.00 | $0.00 | $647.01 | $1,975.02 | 0.00 | 0.00 | 1.30 |
| 1 | No redo | 2.966% | $0.00 | $0.00 | $647.01 | $1,975.02 | 0.00 | 0.00 | 1.30 |
| 1 | Redo in at least 3 years | 0.000% | $0.00 | $0.00 | $647.01 | $1,975.02 | 0.00 | 0.00 | 1.30 |
| 1 | comply | 0.000% | $0.00 | $0.00 | $647.01 | $1,975.02 | 0.00 | 0.00 | 1.30 |
| 1 | Not comply | 0.000% | $0.00 | $0.00 | $647.01 | $1,975.02 | 0.00 | 0.00 | 1.30 |
| 1 | No redo | 13.691% | $0.00 | $0.00 | $647.01 | $1,975.02 | 0.00 | 0.00 | 1.30 |
| 1 | Adenoma | 0.000% | $0.00 | $0.00 | $647.01 | $1,975.02 | 0.00 | 0.00 | 1.30 |
| 1 | Cancerous | 0.000% | $0.00 | $0.00 | $647.01 | $1,975.02 | 0.00 | 0.00 | 1.30 |
| 1 | Early | 0.000% | $0.00 | $0.00 | $647.01 | $1,975.02 | 0.00 | 0.00 | 1.30 |
| 1 | Advanced | 0.000% | $0.00 | $0.00 | $647.01 | $1,975.02 | 0.00 | 0.00 | 1.30 |
| 1 | Noncancerous | 0.000% | $0.00 | $0.00 | $647.01 | $1,975.02 | 0.00 | 0.00 | 1.30 |
| 1 | No adenoma | 0.000% | $0.00 | $0.00 | $647.01 | $1,975.02 | 0.00 | 0.00 | 1.30 |
| 2 | comply | 45.064% | $0.00 | $0.00 | $214.49 | $2,189.51 | 0.85 | 0.38 | 2.11 |
| 2 | Non-comply post colonoscopy | 4.589% | $0.00 | $0.00 | $214.49 | $2,189.51 | 0.00 | 0.00 | 2.11 |
| 2 | Early CRC | 2.715% | $4,170.99 | $113.23 | $214.49 | $2,189.51 | 0.70 | 0.02 | 2.11 |
| 2 | Advanced CRC | 0.384% | $5,617.87 | $21.57 | $214.49 | $2,189.51 | 0.50 | 0.00 | 2.11 |
| 2 | Die other causes | 36.636% | $0.00 | $0.00 | $214.49 | $2,189.51 | 0.85 | 0.31 | 2.11 |
| 2 | Colonoscopy with PureVu due added to inadequate prep or not comply | 10.517% | $706.95 | $74.35 | $214.49 | $2,189.51 | 0.86 | 0.09 | 2.11 |
| 2 | Adenoma surveillance | 0.000% | $2,876.05 | $0.00 | $214.49 | $2,189.51 | 0.86 | 0.00 | 2.11 |
| 2 | Non-compliance with system | 0.000% | $0.00 | $0.00 | $214.49 | $2,189.51 | 0.00 | 0.00 | 2.11 |
| 2 | Dead | 0.095% | $5,617.87 | $5.34 | $214.49 | $2,189.51 | 0.00 | 0.00 | 2.11 |
| 2 | Screening every 10 years - average risk | 0.000% | $0.00 | $0.00 | $214.49 | $2,189.51 | 0.00 | 0.00 | 2.11 |
| 2 | Adequate Prep | 0.000% | $0.00 | $0.00 | $214.49 | $2,189.51 | 0.00 | 0.00 | 2.11 |
| 2 | no adenoma screening | 0.000% | $0.00 | $0.00 | $214.49 | $2,189.51 | 0.00 | 0.00 | 2.11 |
| 2 | Colonoscopy TN | 0.000% | $0.00 | $0.00 | $214.49 | $2,189.51 | 0.00 | 0.00 | 2.11 |
| 2 | Comply | 0.000% | $0.00 | $0.00 | $214.49 | $2,189.51 | 0.00 | 0.00 | 2.11 |
| 2 | Non-comply | 0.000% | $0.00 | $0.00 | $214.49 | $2,189.51 | 0.00 | 0.00 | 2.11 |
| 2 | Colonoscopy FP | 0.000% | $0.00 | $0.00 | $214.49 | $2,189.51 | 0.00 | 0.00 | 2.11 |
| 2 | adenoma | 0.000% | $0.00 | $0.00 | $214.49 | $2,189.51 | 0.00 | 0.00 | 2.11 |
| 2 | Distal Colon (includes descending, sigmoid colon, splenic flexure, rectum) | 0.000% | $0.00 | $0.00 | $214.49 | $2,189.51 | 0.00 | 0.00 | 2.11 |
| 2 | Colonoscopy TP | 0.000% | $0.00 | $0.00 | $214.49 | $2,189.51 | 0.00 | 0.00 | 2.11 |
| 2 | Colonoscopy FN | 0.000% | $0.00 | $0.00 | $214.49 | $2,189.51 | 0.00 | 0.00 | 2.11 |
| 2 | cancer | 0.000% | $0.00 | $0.00 | $214.49 | $2,189.51 | 0.00 | 0.00 | 2.11 |
| 2 | early | 0.000% | $0.00 | $0.00 | $214.49 | $2,189.51 | 0.00 | 0.00 | 2.11 |
| 2 | late | 0.000% | $0.00 | $0.00 | $214.49 | $2,189.51 | 0.00 | 0.00 | 2.11 |
| 2 | no cancer | 0.000% | $0.00 | $0.00 | $214.49 | $2,189.51 | 0.00 | 0.00 | 2.11 |
| 2 | Comply | 0.000% | $0.00 | $0.00 | $214.49 | $2,189.51 | 0.00 | 0.00 | 2.11 |
| 2 | Non-comply | 0.000% | $0.00 | $0.00 | $214.49 | $2,189.51 | 0.00 | 0.00 | 2.11 |
| 2 | Proximal colon (includes ascending, transverse) | 0.000% | $0.00 | $0.00 | $214.49 | $2,189.51 | 0.00 | 0.00 | 2.11 |
| 2 | Colonoscopy TP | 0.000% | $0.00 | $0.00 | $214.49 | $2,189.51 | 0.00 | 0.00 | 2.11 |
| 2 | Colonoscopy FN | 0.000% | $0.00 | $0.00 | $214.49 | $2,189.51 | 0.00 | 0.00 | 2.11 |
| 2 | cancer | 0.000% | $0.00 | $0.00 | $214.49 | $2,189.51 | 0.00 | 0.00 | 2.11 |
| 2 | early | 0.000% | $0.00 | $0.00 | $214.49 | $2,189.51 | 0.00 | 0.00 | 2.11 |
| 2 | late | 0.000% | $0.00 | $0.00 | $214.49 | $2,189.51 | 0.00 | 0.00 | 2.11 |
| 2 | no cancer | 0.000% | $0.00 | $0.00 | $214.49 | $2,189.51 | 0.00 | 0.00 | 2.11 |
| 2 | Comply | 0.000% | $0.00 | $0.00 | $214.49 | $2,189.51 | 0.00 | 0.00 | 2.11 |
| 2 | Non-comply | 0.000% | $0.00 | $0.00 | $214.49 | $2,189.51 | 0.00 | 0.00 | 2.11 |
| 2 | Inadequate Prep | 0.000% | $0.00 | $0.00 | $214.49 | $2,189.51 | 0.00 | 0.00 | 2.11 |
| 2 | PureVu usage | 0.000% | $0.00 | $0.00 | $214.49 | $2,189.51 | 0.00 | 0.00 | 2.11 |
| 2 | Colonoscopy FN | 0.000% | $0.00 | $0.00 | $214.49 | $2,189.51 | 0.00 | 0.00 | 2.11 |
| 2 | cancer | 0.000% | $0.00 | $0.00 | $214.49 | $2,189.51 | 0.00 | 0.00 | 2.11 |
| 2 | early | 0.000% | $0.00 | $0.00 | $214.49 | $2,189.51 | 0.00 | 0.00 | 2.11 |
| 2 | late | 0.000% | $0.00 | $0.00 | $214.49 | $2,189.51 | 0.00 | 0.00 | 2.11 |
| 2 | no cancer | 0.000% | $0.00 | $0.00 | $214.49 | $2,189.51 | 0.00 | 0.00 | 2.11 |
| 2 | Comply | 0.000% | $0.00 | $0.00 | $214.49 | $2,189.51 | 0.00 | 0.00 | 2.11 |
| 2 | Non-comply | 0.000% | $0.00 | $0.00 | $214.49 | $2,189.51 | 0.00 | 0.00 | 2.11 |
| 2 | No screening | 45.064% | $0.00 | $0.00 | $214.49 | $2,189.51 | 0.00 | 0.00 | 2.11 |
| 2 | Adenoma | 1.989% | $0.00 | $0.00 | $214.49 | $2,189.51 | 0.00 | 0.00 | 2.11 |
| 2 | Cancerous | 0.008% | $0.00 | $0.00 | $214.49 | $2,189.51 | 0.00 | 0.00 | 2.11 |
| 2 | Early | 0.007% | $0.00 | $0.00 | $214.49 | $2,189.51 | 0.00 | 0.00 | 2.11 |
| 2 | Advanced | 0.001% | $0.00 | $0.00 | $214.49 | $2,189.51 | 0.00 | 0.00 | 2.11 |
| 2 | Noncancerous | 1.980% | $0.00 | $0.00 | $214.49 | $2,189.51 | 0.00 | 0.00 | 2.11 |
| 2 | comply | 1.406% | $0.00 | $0.00 | $214.49 | $2,189.51 | 0.00 | 0.00 | 2.11 |
| 2 | Not comply | 0.574% | $0.00 | $0.00 | $214.49 | $2,189.51 | 0.00 | 0.00 | 2.11 |
| 2 | No adenoma | 2.601% | $0.00 | $0.00 | $214.49 | $2,189.51 | 0.00 | 0.00 | 2.11 |
| 2 | comply | 1.568% | $0.00 | $0.00 | $214.49 | $2,189.51 | 0.00 | 0.00 | 2.11 |
| 2 | Not comply | 1.032% | $0.00 | $0.00 | $214.49 | $2,189.51 | 0.00 | 0.00 | 2.11 |
| 2 | Continue | 0.307% | $0.00 | $0.00 | $214.49 | $2,189.51 | 0.00 | 0.00 | 2.11 |
| 2 | Die | 0.077% | $0.00 | $0.00 | $214.49 | $2,189.51 | 0.00 | 0.00 | 2.11 |
| 2 | PureVu usage | 8.203% | $0.00 | $0.00 | $214.49 | $2,189.51 | 0.00 | 0.00 | 2.11 |
| 2 | Later followup | 2.314% | $0.00 | $0.00 | $214.49 | $2,189.51 | 0.00 | 0.00 | 2.11 |
| 2 | Redo 2 years | 2.314% | $0.00 | $0.00 | $214.49 | $2,189.51 | 0.00 | 0.00 | 2.11 |
| 2 | comply | 1.293% | $0.00 | $0.00 | $214.49 | $2,189.51 | 0.00 | 0.00 | 2.11 |
| 2 | Not comply | 1.020% | $0.00 | $0.00 | $214.49 | $2,189.51 | 0.00 | 0.00 | 2.11 |
| 2 | No redo | 0.000% | $0.00 | $0.00 | $214.49 | $2,189.51 | 0.00 | 0.00 | 2.11 |
| 2 | Redo in at least 3 years | 0.000% | $0.00 | $0.00 | $214.49 | $2,189.51 | 0.00 | 0.00 | 2.11 |
| 2 | comply | 0.000% | $0.00 | $0.00 | $214.49 | $2,189.51 | 0.00 | 0.00 | 2.11 |
| 2 | Not comply | 0.000% | $0.00 | $0.00 | $214.49 | $2,189.51 | 0.00 | 0.00 | 2.11 |
| 2 | No redo | 0.000% | $0.00 | $0.00 | $214.49 | $2,189.51 | 0.00 | 0.00 | 2.11 |
| 2 | Adenoma | 0.000% | $0.00 | $0.00 | $214.49 | $2,189.51 | 0.00 | 0.00 | 2.11 |
| 2 | Cancerous | 0.000% | $0.00 | $0.00 | $214.49 | $2,189.51 | 0.00 | 0.00 | 2.11 |
| 2 | Early | 0.000% | $0.00 | $0.00 | $214.49 | $2,189.51 | 0.00 | 0.00 | 2.11 |
| 2 | Advanced | 0.000% | $0.00 | $0.00 | $214.49 | $2,189.51 | 0.00 | 0.00 | 2.11 |
| 2 | Noncancerous | 0.000% | $0.00 | $0.00 | $214.49 | $2,189.51 | 0.00 | 0.00 | 2.11 |
| 2 | No adenoma | 0.000% | $0.00 | $0.00 | $214.49 | $2,189.51 | 0.00 | 0.00 | 2.11 |
| 3 | comply | 49.332% | $0.00 | $0.00 | $192.72 | $2,382.23 | 0.82 | 0.41 | 2.91 |
| 3 | Non-comply post colonoscopy | 2.627% | $0.00 | $0.00 | $192.72 | $2,382.23 | 0.00 | 0.00 | 2.91 |
| 3 | Early CRC | 2.722% | $4,049.50 | $110.22 | $192.72 | $2,382.23 | 0.68 | 0.02 | 2.91 |
| 3 | Advanced CRC | 0.308% | $5,454.24 | $16.82 | $192.72 | $2,382.23 | 0.48 | 0.00 | 2.91 |
| 3 | Die other causes | 36.636% | $0.00 | $0.00 | $192.72 | $2,382.23 | 0.83 | 0.30 | 2.91 |
| 3 | Colonoscopy with PureVu due added to inadequate prep or not comply | 8.203% | $686.36 | $56.30 | $192.72 | $2,382.23 | 0.83 | 0.07 | 2.91 |
| 3 | Adenoma surveillance | 0.000% | $2,792.29 | $0.00 | $192.72 | $2,382.23 | 0.83 | 0.00 | 2.91 |
| 3 | Non-compliance with system | 0.000% | $0.00 | $0.00 | $192.72 | $2,382.23 | 0.00 | 0.00 | 2.91 |
| 3 | Dead | 0.172% | $5,454.24 | $9.37 | $192.72 | $2,382.23 | 0.00 | 0.00 | 2.91 |
| 3 | Screening every 10 years - average risk | 0.000% | $0.00 | $0.00 | $192.72 | $2,382.23 | 0.00 | 0.00 | 2.91 |
| 3 | Adequate Prep | 0.000% | $0.00 | $0.00 | $192.72 | $2,382.23 | 0.00 | 0.00 | 2.91 |
| 3 | no adenoma screening | 0.000% | $0.00 | $0.00 | $192.72 | $2,382.23 | 0.00 | 0.00 | 2.91 |
| 3 | Colonoscopy TN | 0.000% | $0.00 | $0.00 | $192.72 | $2,382.23 | 0.00 | 0.00 | 2.91 |
| 3 | Comply | 0.000% | $0.00 | $0.00 | $192.72 | $2,382.23 | 0.00 | 0.00 | 2.91 |
| 3 | Non-comply | 0.000% | $0.00 | $0.00 | $192.72 | $2,382.23 | 0.00 | 0.00 | 2.91 |
| 3 | Colonoscopy FP | 0.000% | $0.00 | $0.00 | $192.72 | $2,382.23 | 0.00 | 0.00 | 2.91 |
| 3 | adenoma | 0.000% | $0.00 | $0.00 | $192.72 | $2,382.23 | 0.00 | 0.00 | 2.91 |
| 3 | Distal Colon (includes descending, sigmoid colon, splenic flexure, rectum) | 0.000% | $0.00 | $0.00 | $192.72 | $2,382.23 | 0.00 | 0.00 | 2.91 |
| 3 | Colonoscopy TP | 0.000% | $0.00 | $0.00 | $192.72 | $2,382.23 | 0.00 | 0.00 | 2.91 |
| 3 | Colonoscopy FN | 0.000% | $0.00 | $0.00 | $192.72 | $2,382.23 | 0.00 | 0.00 | 2.91 |
| 3 | cancer | 0.000% | $0.00 | $0.00 | $192.72 | $2,382.23 | 0.00 | 0.00 | 2.91 |
| 3 | early | 0.000% | $0.00 | $0.00 | $192.72 | $2,382.23 | 0.00 | 0.00 | 2.91 |
| 3 | late | 0.000% | $0.00 | $0.00 | $192.72 | $2,382.23 | 0.00 | 0.00 | 2.91 |
| 3 | no cancer | 0.000% | $0.00 | $0.00 | $192.72 | $2,382.23 | 0.00 | 0.00 | 2.91 |
| 3 | Comply | 0.000% | $0.00 | $0.00 | $192.72 | $2,382.23 | 0.00 | 0.00 | 2.91 |
| 3 | Non-comply | 0.000% | $0.00 | $0.00 | $192.72 | $2,382.23 | 0.00 | 0.00 | 2.91 |
| 3 | Proximal colon (includes ascending, transverse) | 0.000% | $0.00 | $0.00 | $192.72 | $2,382.23 | 0.00 | 0.00 | 2.91 |
| 3 | Colonoscopy TP | 0.000% | $0.00 | $0.00 | $192.72 | $2,382.23 | 0.00 | 0.00 | 2.91 |
| 3 | Colonoscopy FN | 0.000% | $0.00 | $0.00 | $192.72 | $2,382.23 | 0.00 | 0.00 | 2.91 |
| 3 | cancer | 0.000% | $0.00 | $0.00 | $192.72 | $2,382.23 | 0.00 | 0.00 | 2.91 |
| 3 | early | 0.000% | $0.00 | $0.00 | $192.72 | $2,382.23 | 0.00 | 0.00 | 2.91 |
| 3 | late | 0.000% | $0.00 | $0.00 | $192.72 | $2,382.23 | 0.00 | 0.00 | 2.91 |
| 3 | no cancer | 0.000% | $0.00 | $0.00 | $192.72 | $2,382.23 | 0.00 | 0.00 | 2.91 |
| 3 | Comply | 0.000% | $0.00 | $0.00 | $192.72 | $2,382.23 | 0.00 | 0.00 | 2.91 |
| 3 | Non-comply | 0.000% | $0.00 | $0.00 | $192.72 | $2,382.23 | 0.00 | 0.00 | 2.91 |
| 3 | Inadequate Prep | 0.000% | $0.00 | $0.00 | $192.72 | $2,382.23 | 0.00 | 0.00 | 2.91 |
| 3 | PureVu usage | 0.000% | $0.00 | $0.00 | $192.72 | $2,382.23 | 0.00 | 0.00 | 2.91 |
| 3 | Colonoscopy FN | 0.000% | $0.00 | $0.00 | $192.72 | $2,382.23 | 0.00 | 0.00 | 2.91 |
| 3 | cancer | 0.000% | $0.00 | $0.00 | $192.72 | $2,382.23 | 0.00 | 0.00 | 2.91 |
| 3 | early | 0.000% | $0.00 | $0.00 | $192.72 | $2,382.23 | 0.00 | 0.00 | 2.91 |
| 3 | late | 0.000% | $0.00 | $0.00 | $192.72 | $2,382.23 | 0.00 | 0.00 | 2.91 |
| 3 | no cancer | 0.000% | $0.00 | $0.00 | $192.72 | $2,382.23 | 0.00 | 0.00 | 2.91 |
| 3 | Comply | 0.000% | $0.00 | $0.00 | $192.72 | $2,382.23 | 0.00 | 0.00 | 2.91 |
| 3 | Non-comply | 0.000% | $0.00 | $0.00 | $192.72 | $2,382.23 | 0.00 | 0.00 | 2.91 |
| 3 | No screening | 49.332% | $0.00 | $0.00 | $192.72 | $2,382.23 | 0.00 | 0.00 | 2.91 |
| 3 | Adenoma | 1.138% | $0.00 | $0.00 | $192.72 | $2,382.23 | 0.00 | 0.00 | 2.91 |
| 3 | Cancerous | 0.005% | $0.00 | $0.00 | $192.72 | $2,382.23 | 0.00 | 0.00 | 2.91 |
| 3 | Early | 0.004% | $0.00 | $0.00 | $192.72 | $2,382.23 | 0.00 | 0.00 | 2.91 |
| 3 | Advanced | 0.001% | $0.00 | $0.00 | $192.72 | $2,382.23 | 0.00 | 0.00 | 2.91 |
| 3 | Noncancerous | 1.134% | $0.00 | $0.00 | $192.72 | $2,382.23 | 0.00 | 0.00 | 2.91 |
| 3 | comply | 0.805% | $0.00 | $0.00 | $192.72 | $2,382.23 | 0.00 | 0.00 | 2.91 |
| 3 | Not comply | 0.329% | $0.00 | $0.00 | $192.72 | $2,382.23 | 0.00 | 0.00 | 2.91 |
| 3 | No adenoma | 1.489% | $0.00 | $0.00 | $192.72 | $2,382.23 | 0.00 | 0.00 | 2.91 |
| 3 | comply | 0.898% | $0.00 | $0.00 | $192.72 | $2,382.23 | 0.00 | 0.00 | 2.91 |
| 3 | Not comply | 0.591% | $0.00 | $0.00 | $192.72 | $2,382.23 | 0.00 | 0.00 | 2.91 |
| 3 | Continue | 0.247% | $0.00 | $0.00 | $192.72 | $2,382.23 | 0.00 | 0.00 | 2.91 |
| 3 | Die | 0.062% | $0.00 | $0.00 | $192.72 | $2,382.23 | 0.00 | 0.00 | 2.91 |
| 3 | PureVu usage | 6.398% | $0.00 | $0.00 | $192.72 | $2,382.23 | 0.00 | 0.00 | 2.91 |
| 3 | Later followup | 1.805% | $0.00 | $0.00 | $192.72 | $2,382.23 | 0.00 | 0.00 | 2.91 |
| 3 | Redo 2 years | 0.000% | $0.00 | $0.00 | $192.72 | $2,382.23 | 0.00 | 0.00 | 2.91 |
| 3 | comply | 0.000% | $0.00 | $0.00 | $192.72 | $2,382.23 | 0.00 | 0.00 | 2.91 |
| 3 | Not comply | 0.000% | $0.00 | $0.00 | $192.72 | $2,382.23 | 0.00 | 0.00 | 2.91 |
| 3 | No redo | 1.805% | $0.00 | $0.00 | $192.72 | $2,382.23 | 0.00 | 0.00 | 2.91 |
| 3 | Redo in at least 3 years | 0.000% | $0.00 | $0.00 | $192.72 | $2,382.23 | 0.00 | 0.00 | 2.91 |
| 3 | comply | 0.000% | $0.00 | $0.00 | $192.72 | $2,382.23 | 0.00 | 0.00 | 2.91 |
| 3 | Not comply | 0.000% | $0.00 | $0.00 | $192.72 | $2,382.23 | 0.00 | 0.00 | 2.91 |
| 3 | No redo | 0.000% | $0.00 | $0.00 | $192.72 | $2,382.23 | 0.00 | 0.00 | 2.91 |
| 3 | Adenoma | 0.000% | $0.00 | $0.00 | $192.72 | $2,382.23 | 0.00 | 0.00 | 2.91 |
| 3 | Cancerous | 0.000% | $0.00 | $0.00 | $192.72 | $2,382.23 | 0.00 | 0.00 | 2.91 |
| 3 | Early | 0.000% | $0.00 | $0.00 | $192.72 | $2,382.23 | 0.00 | 0.00 | 2.91 |
| 3 | Advanced | 0.000% | $0.00 | $0.00 | $192.72 | $2,382.23 | 0.00 | 0.00 | 2.91 |
| 3 | Noncancerous | 0.000% | $0.00 | $0.00 | $192.72 | $2,382.23 | 0.00 | 0.00 | 2.91 |
| 3 | No adenoma | 0.000% | $0.00 | $0.00 | $192.72 | $2,382.23 | 0.00 | 0.00 | 2.91 |
| 4 | comply | 52.839% | $0.00 | $0.00 | $175.28 | $2,557.51 | 0.80 | 0.42 | 3.69 |
| 4 | Non-comply post colonoscopy | 0.920% | $0.00 | $0.00 | $175.28 | $2,557.51 | 0.00 | 0.00 | 3.69 |
| 4 | Early CRC | 2.726% | $3,931.56 | $107.17 | $175.28 | $2,557.51 | 0.66 | 0.02 | 3.69 |
| 4 | Advanced CRC | 0.247% | $5,295.38 | $13.10 | $175.28 | $2,557.51 | 0.47 | 0.00 | 3.69 |
| 4 | Die other causes | 36.636% | $0.00 | $0.00 | $175.28 | $2,557.51 | 0.81 | 0.30 | 3.69 |
| 4 | Colonoscopy with PureVu due added to inadequate prep or not comply | 6.398% | $666.37 | $42.64 | $175.28 | $2,557.51 | 0.81 | 0.05 | 3.69 |
| 4 | Adenoma surveillance | 0.000% | $2,710.96 | $0.00 | $175.28 | $2,557.51 | 0.81 | 0.00 | 3.69 |
| 4 | Non-compliance with system | 0.000% | $0.00 | $0.00 | $175.28 | $2,557.51 | 0.00 | 0.00 | 3.69 |
| 4 | Dead | 0.234% | $5,295.38 | $12.37 | $175.28 | $2,557.51 | 0.00 | 0.00 | 3.69 |
| 4 | Screening every 10 years - average risk | 0.000% | $0.00 | $0.00 | $175.28 | $2,557.51 | 0.00 | 0.00 | 3.69 |
| 4 | Adequate Prep | 0.000% | $0.00 | $0.00 | $175.28 | $2,557.51 | 0.00 | 0.00 | 3.69 |
| 4 | no adenoma screening | 0.000% | $0.00 | $0.00 | $175.28 | $2,557.51 | 0.00 | 0.00 | 3.69 |
| 4 | Colonoscopy TN | 0.000% | $0.00 | $0.00 | $175.28 | $2,557.51 | 0.00 | 0.00 | 3.69 |
| 4 | Comply | 0.000% | $0.00 | $0.00 | $175.28 | $2,557.51 | 0.00 | 0.00 | 3.69 |
| 4 | Non-comply | 0.000% | $0.00 | $0.00 | $175.28 | $2,557.51 | 0.00 | 0.00 | 3.69 |
| 4 | Colonoscopy FP | 0.000% | $0.00 | $0.00 | $175.28 | $2,557.51 | 0.00 | 0.00 | 3.69 |
| 4 | adenoma | 0.000% | $0.00 | $0.00 | $175.28 | $2,557.51 | 0.00 | 0.00 | 3.69 |
| 4 | Distal Colon (includes descending, sigmoid colon, splenic flexure, rectum) | 0.000% | $0.00 | $0.00 | $175.28 | $2,557.51 | 0.00 | 0.00 | 3.69 |
| 4 | Colonoscopy TP | 0.000% | $0.00 | $0.00 | $175.28 | $2,557.51 | 0.00 | 0.00 | 3.69 |
| 4 | Colonoscopy FN | 0.000% | $0.00 | $0.00 | $175.28 | $2,557.51 | 0.00 | 0.00 | 3.69 |
| 4 | cancer | 0.000% | $0.00 | $0.00 | $175.28 | $2,557.51 | 0.00 | 0.00 | 3.69 |
| 4 | early | 0.000% | $0.00 | $0.00 | $175.28 | $2,557.51 | 0.00 | 0.00 | 3.69 |
| 4 | late | 0.000% | $0.00 | $0.00 | $175.28 | $2,557.51 | 0.00 | 0.00 | 3.69 |
| 4 | no cancer | 0.000% | $0.00 | $0.00 | $175.28 | $2,557.51 | 0.00 | 0.00 | 3.69 |
| 4 | Comply | 0.000% | $0.00 | $0.00 | $175.28 | $2,557.51 | 0.00 | 0.00 | 3.69 |
| 4 | Non-comply | 0.000% | $0.00 | $0.00 | $175.28 | $2,557.51 | 0.00 | 0.00 | 3.69 |
| 4 | Proximal colon (includes ascending, transverse) | 0.000% | $0.00 | $0.00 | $175.28 | $2,557.51 | 0.00 | 0.00 | 3.69 |
| 4 | Colonoscopy TP | 0.000% | $0.00 | $0.00 | $175.28 | $2,557.51 | 0.00 | 0.00 | 3.69 |
| 4 | Colonoscopy FN | 0.000% | $0.00 | $0.00 | $175.28 | $2,557.51 | 0.00 | 0.00 | 3.69 |
| 4 | cancer | 0.000% | $0.00 | $0.00 | $175.28 | $2,557.51 | 0.00 | 0.00 | 3.69 |
| 4 | early | 0.000% | $0.00 | $0.00 | $175.28 | $2,557.51 | 0.00 | 0.00 | 3.69 |
| 4 | late | 0.000% | $0.00 | $0.00 | $175.28 | $2,557.51 | 0.00 | 0.00 | 3.69 |
| 4 | no cancer | 0.000% | $0.00 | $0.00 | $175.28 | $2,557.51 | 0.00 | 0.00 | 3.69 |
| 4 | Comply | 0.000% | $0.00 | $0.00 | $175.28 | $2,557.51 | 0.00 | 0.00 | 3.69 |
| 4 | Non-comply | 0.000% | $0.00 | $0.00 | $175.28 | $2,557.51 | 0.00 | 0.00 | 3.69 |
| 4 | Inadequate Prep | 0.000% | $0.00 | $0.00 | $175.28 | $2,557.51 | 0.00 | 0.00 | 3.69 |
| 4 | PureVu usage | 0.000% | $0.00 | $0.00 | $175.28 | $2,557.51 | 0.00 | 0.00 | 3.69 |
| 4 | Colonoscopy FN | 0.000% | $0.00 | $0.00 | $175.28 | $2,557.51 | 0.00 | 0.00 | 3.69 |
| 4 | cancer | 0.000% | $0.00 | $0.00 | $175.28 | $2,557.51 | 0.00 | 0.00 | 3.69 |
| 4 | early | 0.000% | $0.00 | $0.00 | $175.28 | $2,557.51 | 0.00 | 0.00 | 3.69 |
| 4 | late | 0.000% | $0.00 | $0.00 | $175.28 | $2,557.51 | 0.00 | 0.00 | 3.69 |
| 4 | no cancer | 0.000% | $0.00 | $0.00 | $175.28 | $2,557.51 | 0.00 | 0.00 | 3.69 |
| 4 | Comply | 0.000% | $0.00 | $0.00 | $175.28 | $2,557.51 | 0.00 | 0.00 | 3.69 |
| 4 | Non-comply | 0.000% | $0.00 | $0.00 | $175.28 | $2,557.51 | 0.00 | 0.00 | 3.69 |
| 4 | No screening | 52.839% | $0.00 | $0.00 | $175.28 | $2,557.51 | 0.00 | 0.00 | 3.69 |
| 4 | Adenoma | 0.399% | $0.00 | $0.00 | $175.28 | $2,557.51 | 0.00 | 0.00 | 3.69 |
| 4 | Cancerous | 0.002% | $0.00 | $0.00 | $175.28 | $2,557.51 | 0.00 | 0.00 | 3.69 |
| 4 | Early | 0.001% | $0.00 | $0.00 | $175.28 | $2,557.51 | 0.00 | 0.00 | 3.69 |
| 4 | Advanced | 0.000% | $0.00 | $0.00 | $175.28 | $2,557.51 | 0.00 | 0.00 | 3.69 |
| 4 | Noncancerous | 0.397% | $0.00 | $0.00 | $175.28 | $2,557.51 | 0.00 | 0.00 | 3.69 |
| 4 | comply | 0.282% | $0.00 | $0.00 | $175.28 | $2,557.51 | 0.00 | 0.00 | 3.69 |
| 4 | Not comply | 0.115% | $0.00 | $0.00 | $175.28 | $2,557.51 | 0.00 | 0.00 | 3.69 |
| 4 | No adenoma | 0.521% | $0.00 | $0.00 | $175.28 | $2,557.51 | 0.00 | 0.00 | 3.69 |
| 4 | comply | 0.314% | $0.00 | $0.00 | $175.28 | $2,557.51 | 0.00 | 0.00 | 3.69 |
| 4 | Not comply | 0.207% | $0.00 | $0.00 | $175.28 | $2,557.51 | 0.00 | 0.00 | 3.69 |
| 4 | Continue | 0.198% | $0.00 | $0.00 | $175.28 | $2,557.51 | 0.00 | 0.00 | 3.69 |
| 4 | Die | 0.049% | $0.00 | $0.00 | $175.28 | $2,557.51 | 0.00 | 0.00 | 3.69 |
| 4 | PureVu usage | 4.991% | $0.00 | $0.00 | $175.28 | $2,557.51 | 0.00 | 0.00 | 3.69 |
| 4 | Later followup | 1.408% | $0.00 | $0.00 | $175.28 | $2,557.51 | 0.00 | 0.00 | 3.69 |
| 4 | Redo 2 years | 1.408% | $0.00 | $0.00 | $175.28 | $2,557.51 | 0.00 | 0.00 | 3.69 |
| 4 | comply | 0.787% | $0.00 | $0.00 | $175.28 | $2,557.51 | 0.00 | 0.00 | 3.69 |
| 4 | Not comply | 0.621% | $0.00 | $0.00 | $175.28 | $2,557.51 | 0.00 | 0.00 | 3.69 |
| 4 | No redo | 0.000% | $0.00 | $0.00 | $175.28 | $2,557.51 | 0.00 | 0.00 | 3.69 |
| 4 | Redo in at least 3 years | 0.000% | $0.00 | $0.00 | $175.28 | $2,557.51 | 0.00 | 0.00 | 3.69 |
| 4 | comply | 0.000% | $0.00 | $0.00 | $175.28 | $2,557.51 | 0.00 | 0.00 | 3.69 |
| 4 | Not comply | 0.000% | $0.00 | $0.00 | $175.28 | $2,557.51 | 0.00 | 0.00 | 3.69 |
| 4 | No redo | 0.000% | $0.00 | $0.00 | $175.28 | $2,557.51 | 0.00 | 0.00 | 3.69 |
| 4 | Adenoma | 0.000% | $0.00 | $0.00 | $175.28 | $2,557.51 | 0.00 | 0.00 | 3.69 |
| 4 | Cancerous | 0.000% | $0.00 | $0.00 | $175.28 | $2,557.51 | 0.00 | 0.00 | 3.69 |
| 4 | Early | 0.000% | $0.00 | $0.00 | $175.28 | $2,557.51 | 0.00 | 0.00 | 3.69 |
| 4 | Advanced | 0.000% | $0.00 | $0.00 | $175.28 | $2,557.51 | 0.00 | 0.00 | 3.69 |
| 4 | Noncancerous | 0.000% | $0.00 | $0.00 | $175.28 | $2,557.51 | 0.00 | 0.00 | 3.69 |
| 4 | No adenoma | 0.000% | $0.00 | $0.00 | $175.28 | $2,557.51 | 0.00 | 0.00 | 3.69 |
| 5 | comply | 54.222% | $0.00 | $0.00 | $161.14 | $2,718.64 | 0.78 | 0.42 | 4.46 |
| 5 | Non-comply post colonoscopy | 0.943% | $0.00 | $0.00 | $161.14 | $2,718.64 | 0.00 | 0.00 | 4.46 |
| 5 | Early CRC | 2.727% | $3,817.04 | $104.10 | $161.14 | $2,718.64 | 0.64 | 0.02 | 4.46 |
| 5 | Advanced CRC | 0.198% | $5,141.15 | $10.19 | $161.14 | $2,718.64 | 0.45 | 0.00 | 4.46 |
| 5 | Die other causes | 36.636% | $0.00 | $0.00 | $161.14 | $2,718.64 | 0.78 | 0.29 | 4.46 |
| 5 | Colonoscopy with PureVu due added to inadequate prep or not comply | 4.991% | $646.96 | $32.29 | $161.14 | $2,718.64 | 0.78 | 0.04 | 4.46 |
| 5 | Adenoma surveillance | 0.000% | $2,632.00 | $0.00 | $161.14 | $2,718.64 | 0.78 | 0.00 | 4.46 |
| 5 | Non-compliance with system | 0.000% | $0.00 | $0.00 | $161.14 | $2,718.64 | 0.00 | 0.00 | 4.46 |
| 5 | Dead | 0.283% | $5,141.15 | $14.55 | $161.14 | $2,718.64 | 0.00 | 0.00 | 4.46 |
| 5 | Screening every 10 years - average risk | 0.000% | $0.00 | $0.00 | $161.14 | $2,718.64 | 0.00 | 0.00 | 4.46 |
| 5 | Adequate Prep | 0.000% | $0.00 | $0.00 | $161.14 | $2,718.64 | 0.00 | 0.00 | 4.46 |
| 5 | no adenoma screening | 0.000% | $0.00 | $0.00 | $161.14 | $2,718.64 | 0.00 | 0.00 | 4.46 |
| 5 | Colonoscopy TN | 0.000% | $0.00 | $0.00 | $161.14 | $2,718.64 | 0.00 | 0.00 | 4.46 |
| 5 | Comply | 0.000% | $0.00 | $0.00 | $161.14 | $2,718.64 | 0.00 | 0.00 | 4.46 |
| 5 | Non-comply | 0.000% | $0.00 | $0.00 | $161.14 | $2,718.64 | 0.00 | 0.00 | 4.46 |
| 5 | Colonoscopy FP | 0.000% | $0.00 | $0.00 | $161.14 | $2,718.64 | 0.00 | 0.00 | 4.46 |
| 5 | adenoma | 0.000% | $0.00 | $0.00 | $161.14 | $2,718.64 | 0.00 | 0.00 | 4.46 |
| 5 | Distal Colon (includes descending, sigmoid colon, splenic flexure, rectum) | 0.000% | $0.00 | $0.00 | $161.14 | $2,718.64 | 0.00 | 0.00 | 4.46 |
| 5 | Colonoscopy TP | 0.000% | $0.00 | $0.00 | $161.14 | $2,718.64 | 0.00 | 0.00 | 4.46 |
| 5 | Colonoscopy FN | 0.000% | $0.00 | $0.00 | $161.14 | $2,718.64 | 0.00 | 0.00 | 4.46 |
| 5 | cancer | 0.000% | $0.00 | $0.00 | $161.14 | $2,718.64 | 0.00 | 0.00 | 4.46 |
| 5 | early | 0.000% | $0.00 | $0.00 | $161.14 | $2,718.64 | 0.00 | 0.00 | 4.46 |
| 5 | late | 0.000% | $0.00 | $0.00 | $161.14 | $2,718.64 | 0.00 | 0.00 | 4.46 |
| 5 | no cancer | 0.000% | $0.00 | $0.00 | $161.14 | $2,718.64 | 0.00 | 0.00 | 4.46 |
| 5 | Comply | 0.000% | $0.00 | $0.00 | $161.14 | $2,718.64 | 0.00 | 0.00 | 4.46 |
| 5 | Non-comply | 0.000% | $0.00 | $0.00 | $161.14 | $2,718.64 | 0.00 | 0.00 | 4.46 |
| 5 | Proximal colon (includes ascending, transverse) | 0.000% | $0.00 | $0.00 | $161.14 | $2,718.64 | 0.00 | 0.00 | 4.46 |
| 5 | Colonoscopy TP | 0.000% | $0.00 | $0.00 | $161.14 | $2,718.64 | 0.00 | 0.00 | 4.46 |
| 5 | Colonoscopy FN | 0.000% | $0.00 | $0.00 | $161.14 | $2,718.64 | 0.00 | 0.00 | 4.46 |
| 5 | cancer | 0.000% | $0.00 | $0.00 | $161.14 | $2,718.64 | 0.00 | 0.00 | 4.46 |
| 5 | early | 0.000% | $0.00 | $0.00 | $161.14 | $2,718.64 | 0.00 | 0.00 | 4.46 |
| 5 | late | 0.000% | $0.00 | $0.00 | $161.14 | $2,718.64 | 0.00 | 0.00 | 4.46 |
| 5 | no cancer | 0.000% | $0.00 | $0.00 | $161.14 | $2,718.64 | 0.00 | 0.00 | 4.46 |
| 5 | Comply | 0.000% | $0.00 | $0.00 | $161.14 | $2,718.64 | 0.00 | 0.00 | 4.46 |
| 5 | Non-comply | 0.000% | $0.00 | $0.00 | $161.14 | $2,718.64 | 0.00 | 0.00 | 4.46 |
| 5 | Inadequate Prep | 0.000% | $0.00 | $0.00 | $161.14 | $2,718.64 | 0.00 | 0.00 | 4.46 |
| 5 | PureVu usage | 0.000% | $0.00 | $0.00 | $161.14 | $2,718.64 | 0.00 | 0.00 | 4.46 |
| 5 | Colonoscopy FN | 0.000% | $0.00 | $0.00 | $161.14 | $2,718.64 | 0.00 | 0.00 | 4.46 |
| 5 | cancer | 0.000% | $0.00 | $0.00 | $161.14 | $2,718.64 | 0.00 | 0.00 | 4.46 |
| 5 | early | 0.000% | $0.00 | $0.00 | $161.14 | $2,718.64 | 0.00 | 0.00 | 4.46 |
| 5 | late | 0.000% | $0.00 | $0.00 | $161.14 | $2,718.64 | 0.00 | 0.00 | 4.46 |
| 5 | no cancer | 0.000% | $0.00 | $0.00 | $161.14 | $2,718.64 | 0.00 | 0.00 | 4.46 |
| 5 | Comply | 0.000% | $0.00 | $0.00 | $161.14 | $2,718.64 | 0.00 | 0.00 | 4.46 |
| 5 | Non-comply | 0.000% | $0.00 | $0.00 | $161.14 | $2,718.64 | 0.00 | 0.00 | 4.46 |
| 5 | No screening | 54.222% | $0.00 | $0.00 | $161.14 | $2,718.64 | 0.00 | 0.00 | 4.46 |
| 5 | Adenoma | 0.409% | $0.00 | $0.00 | $161.14 | $2,718.64 | 0.00 | 0.00 | 4.46 |
| 5 | Cancerous | 0.002% | $0.00 | $0.00 | $161.14 | $2,718.64 | 0.00 | 0.00 | 4.46 |
| 5 | Early | 0.001% | $0.00 | $0.00 | $161.14 | $2,718.64 | 0.00 | 0.00 | 4.46 |
| 5 | Advanced | 0.000% | $0.00 | $0.00 | $161.14 | $2,718.64 | 0.00 | 0.00 | 4.46 |
| 5 | Noncancerous | 0.407% | $0.00 | $0.00 | $161.14 | $2,718.64 | 0.00 | 0.00 | 4.46 |
| 5 | comply | 0.289% | $0.00 | $0.00 | $161.14 | $2,718.64 | 0.00 | 0.00 | 4.46 |
| 5 | Not comply | 0.118% | $0.00 | $0.00 | $161.14 | $2,718.64 | 0.00 | 0.00 | 4.46 |
| 5 | No adenoma | 0.534% | $0.00 | $0.00 | $161.14 | $2,718.64 | 0.00 | 0.00 | 4.46 |
| 5 | comply | 0.322% | $0.00 | $0.00 | $161.14 | $2,718.64 | 0.00 | 0.00 | 4.46 |
| 5 | Not comply | 0.212% | $0.00 | $0.00 | $161.14 | $2,718.64 | 0.00 | 0.00 | 4.46 |
| 5 | Continue | 0.159% | $0.00 | $0.00 | $161.14 | $2,718.64 | 0.00 | 0.00 | 4.46 |
| 5 | Die | 0.040% | $0.00 | $0.00 | $161.14 | $2,718.64 | 0.00 | 0.00 | 4.46 |
| 5 | PureVu usage | 3.893% | $0.00 | $0.00 | $161.14 | $2,718.64 | 0.00 | 0.00 | 4.46 |
| 5 | Later followup | 1.098% | $0.00 | $0.00 | $161.14 | $2,718.64 | 0.00 | 0.00 | 4.46 |
| 5 | Redo 2 years | 0.000% | $0.00 | $0.00 | $161.14 | $2,718.64 | 0.00 | 0.00 | 4.46 |
| 5 | comply | 0.000% | $0.00 | $0.00 | $161.14 | $2,718.64 | 0.00 | 0.00 | 4.46 |
| 5 | Not comply | 0.000% | $0.00 | $0.00 | $161.14 | $2,718.64 | 0.00 | 0.00 | 4.46 |
| 5 | No redo | 1.098% | $0.00 | $0.00 | $161.14 | $2,718.64 | 0.00 | 0.00 | 4.46 |
| 5 | Redo in at least 3 years | 0.000% | $0.00 | $0.00 | $161.14 | $2,718.64 | 0.00 | 0.00 | 4.46 |
| 5 | comply | 0.000% | $0.00 | $0.00 | $161.14 | $2,718.64 | 0.00 | 0.00 | 4.46 |
| 5 | Not comply | 0.000% | $0.00 | $0.00 | $161.14 | $2,718.64 | 0.00 | 0.00 | 4.46 |
| 5 | No redo | 0.000% | $0.00 | $0.00 | $161.14 | $2,718.64 | 0.00 | 0.00 | 4.46 |
| 5 | Adenoma | 0.000% | $0.00 | $0.00 | $161.14 | $2,718.64 | 0.00 | 0.00 | 4.46 |
| 5 | Cancerous | 0.000% | $0.00 | $0.00 | $161.14 | $2,718.64 | 0.00 | 0.00 | 4.46 |
| 5 | Early | 0.000% | $0.00 | $0.00 | $161.14 | $2,718.64 | 0.00 | 0.00 | 4.46 |
| 5 | Advanced | 0.000% | $0.00 | $0.00 | $161.14 | $2,718.64 | 0.00 | 0.00 | 4.46 |
| 5 | Noncancerous | 0.000% | $0.00 | $0.00 | $161.14 | $2,718.64 | 0.00 | 0.00 | 4.46 |
| 5 | No adenoma | 0.000% | $0.00 | $0.00 | $161.14 | $2,718.64 | 0.00 | 0.00 | 4.46 |
| 6 | comply | 55.931% | $0.00 | $0.00 | $149.62 | $2,868.26 | 0.75 | 0.42 | 5.21 |
| 6 | Non-comply post colonoscopy | 0.330% | $0.00 | $0.00 | $149.62 | $2,868.26 | 0.00 | 0.00 | 5.21 |
| 6 | Early CRC | 2.729% | $3,705.87 | $101.13 | $149.62 | $2,868.26 | 0.62 | 0.02 | 5.21 |
| 6 | Advanced CRC | 0.159% | $4,991.41 | $7.93 | $149.62 | $2,868.26 | 0.44 | 0.00 | 5.21 |
| 6 | Die other causes | 36.636% | $0.00 | $0.00 | $149.62 | $2,868.26 | 0.76 | 0.28 | 5.21 |
| 6 | Colonoscopy with PureVu due added to inadequate prep or not comply | 3.893% | $628.11 | $24.45 | $149.62 | $2,868.26 | 0.76 | 0.03 | 5.21 |
| 6 | Adenoma surveillance | 0.000% | $2,555.34 | $0.00 | $149.62 | $2,868.26 | 0.76 | 0.00 | 5.21 |
| 6 | Non-compliance with system | 0.000% | $0.00 | $0.00 | $149.62 | $2,868.26 | 0.00 | 0.00 | 5.21 |
| 6 | Dead | 0.323% | $4,991.41 | $16.11 | $149.62 | $2,868.26 | 0.00 | 0.00 | 5.21 |
| 6 | Screening every 10 years - average risk | 0.000% | $0.00 | $0.00 | $149.62 | $2,868.26 | 0.00 | 0.00 | 5.21 |
| 6 | Adequate Prep | 0.000% | $0.00 | $0.00 | $149.62 | $2,868.26 | 0.00 | 0.00 | 5.21 |
| 6 | no adenoma screening | 0.000% | $0.00 | $0.00 | $149.62 | $2,868.26 | 0.00 | 0.00 | 5.21 |
| 6 | Colonoscopy TN | 0.000% | $0.00 | $0.00 | $149.62 | $2,868.26 | 0.00 | 0.00 | 5.21 |
| 6 | Comply | 0.000% | $0.00 | $0.00 | $149.62 | $2,868.26 | 0.00 | 0.00 | 5.21 |
| 6 | Non-comply | 0.000% | $0.00 | $0.00 | $149.62 | $2,868.26 | 0.00 | 0.00 | 5.21 |
| 6 | Colonoscopy FP | 0.000% | $0.00 | $0.00 | $149.62 | $2,868.26 | 0.00 | 0.00 | 5.21 |
| 6 | adenoma | 0.000% | $0.00 | $0.00 | $149.62 | $2,868.26 | 0.00 | 0.00 | 5.21 |
| 6 | Distal Colon (includes descending, sigmoid colon, splenic flexure, rectum) | 0.000% | $0.00 | $0.00 | $149.62 | $2,868.26 | 0.00 | 0.00 | 5.21 |
| 6 | Colonoscopy TP | 0.000% | $0.00 | $0.00 | $149.62 | $2,868.26 | 0.00 | 0.00 | 5.21 |
| 6 | Colonoscopy FN | 0.000% | $0.00 | $0.00 | $149.62 | $2,868.26 | 0.00 | 0.00 | 5.21 |
| 6 | cancer | 0.000% | $0.00 | $0.00 | $149.62 | $2,868.26 | 0.00 | 0.00 | 5.21 |
| 6 | early | 0.000% | $0.00 | $0.00 | $149.62 | $2,868.26 | 0.00 | 0.00 | 5.21 |
| 6 | late | 0.000% | $0.00 | $0.00 | $149.62 | $2,868.26 | 0.00 | 0.00 | 5.21 |
| 6 | no cancer | 0.000% | $0.00 | $0.00 | $149.62 | $2,868.26 | 0.00 | 0.00 | 5.21 |
| 6 | Comply | 0.000% | $0.00 | $0.00 | $149.62 | $2,868.26 | 0.00 | 0.00 | 5.21 |
| 6 | Non-comply | 0.000% | $0.00 | $0.00 | $149.62 | $2,868.26 | 0.00 | 0.00 | 5.21 |
| 6 | Proximal colon (includes ascending, transverse) | 0.000% | $0.00 | $0.00 | $149.62 | $2,868.26 | 0.00 | 0.00 | 5.21 |
| 6 | Colonoscopy TP | 0.000% | $0.00 | $0.00 | $149.62 | $2,868.26 | 0.00 | 0.00 | 5.21 |
| 6 | Colonoscopy FN | 0.000% | $0.00 | $0.00 | $149.62 | $2,868.26 | 0.00 | 0.00 | 5.21 |
| 6 | cancer | 0.000% | $0.00 | $0.00 | $149.62 | $2,868.26 | 0.00 | 0.00 | 5.21 |
| 6 | early | 0.000% | $0.00 | $0.00 | $149.62 | $2,868.26 | 0.00 | 0.00 | 5.21 |
| 6 | late | 0.000% | $0.00 | $0.00 | $149.62 | $2,868.26 | 0.00 | 0.00 | 5.21 |
| 6 | no cancer | 0.000% | $0.00 | $0.00 | $149.62 | $2,868.26 | 0.00 | 0.00 | 5.21 |
| 6 | Comply | 0.000% | $0.00 | $0.00 | $149.62 | $2,868.26 | 0.00 | 0.00 | 5.21 |
| 6 | Non-comply | 0.000% | $0.00 | $0.00 | $149.62 | $2,868.26 | 0.00 | 0.00 | 5.21 |
| 6 | Inadequate Prep | 0.000% | $0.00 | $0.00 | $149.62 | $2,868.26 | 0.00 | 0.00 | 5.21 |
| 6 | PureVu usage | 0.000% | $0.00 | $0.00 | $149.62 | $2,868.26 | 0.00 | 0.00 | 5.21 |
| 6 | Colonoscopy FN | 0.000% | $0.00 | $0.00 | $149.62 | $2,868.26 | 0.00 | 0.00 | 5.21 |
| 6 | cancer | 0.000% | $0.00 | $0.00 | $149.62 | $2,868.26 | 0.00 | 0.00 | 5.21 |
| 6 | early | 0.000% | $0.00 | $0.00 | $149.62 | $2,868.26 | 0.00 | 0.00 | 5.21 |
| 6 | late | 0.000% | $0.00 | $0.00 | $149.62 | $2,868.26 | 0.00 | 0.00 | 5.21 |
| 6 | no cancer | 0.000% | $0.00 | $0.00 | $149.62 | $2,868.26 | 0.00 | 0.00 | 5.21 |
| 6 | Comply | 0.000% | $0.00 | $0.00 | $149.62 | $2,868.26 | 0.00 | 0.00 | 5.21 |
| 6 | Non-comply | 0.000% | $0.00 | $0.00 | $149.62 | $2,868.26 | 0.00 | 0.00 | 5.21 |
| 6 | No screening | 55.931% | $0.00 | $0.00 | $149.62 | $2,868.26 | 0.00 | 0.00 | 5.21 |
| 6 | Adenoma | 0.143% | $0.00 | $0.00 | $149.62 | $2,868.26 | 0.00 | 0.00 | 5.21 |
| 6 | Cancerous | 0.001% | $0.00 | $0.00 | $149.62 | $2,868.26 | 0.00 | 0.00 | 5.21 |
| 6 | Early | 0.001% | $0.00 | $0.00 | $149.62 | $2,868.26 | 0.00 | 0.00 | 5.21 |
| 6 | Advanced | 0.000% | $0.00 | $0.00 | $149.62 | $2,868.26 | 0.00 | 0.00 | 5.21 |
| 6 | Noncancerous | 0.142% | $0.00 | $0.00 | $149.62 | $2,868.26 | 0.00 | 0.00 | 5.21 |
| 6 | comply | 0.101% | $0.00 | $0.00 | $149.62 | $2,868.26 | 0.00 | 0.00 | 5.21 |
| 6 | Not comply | 0.041% | $0.00 | $0.00 | $149.62 | $2,868.26 | 0.00 | 0.00 | 5.21 |
| 6 | No adenoma | 0.187% | $0.00 | $0.00 | $149.62 | $2,868.26 | 0.00 | 0.00 | 5.21 |
| 6 | comply | 0.113% | $0.00 | $0.00 | $149.62 | $2,868.26 | 0.00 | 0.00 | 5.21 |
| 6 | Not comply | 0.074% | $0.00 | $0.00 | $149.62 | $2,868.26 | 0.00 | 0.00 | 5.21 |
| 6 | Continue | 0.127% | $0.00 | $0.00 | $149.62 | $2,868.26 | 0.00 | 0.00 | 5.21 |
| 6 | Die | 0.032% | $0.00 | $0.00 | $149.62 | $2,868.26 | 0.00 | 0.00 | 5.21 |
| 6 | PureVu usage | 3.036% | $0.00 | $0.00 | $149.62 | $2,868.26 | 0.00 | 0.00 | 5.21 |
| 6 | Later followup | 0.856% | $0.00 | $0.00 | $149.62 | $2,868.26 | 0.00 | 0.00 | 5.21 |
| 6 | Redo 2 years | 0.856% | $0.00 | $0.00 | $149.62 | $2,868.26 | 0.00 | 0.00 | 5.21 |
| 6 | comply | 0.479% | $0.00 | $0.00 | $149.62 | $2,868.26 | 0.00 | 0.00 | 5.21 |
| 6 | Not comply | 0.378% | $0.00 | $0.00 | $149.62 | $2,868.26 | 0.00 | 0.00 | 5.21 |
| 6 | No redo | 0.000% | $0.00 | $0.00 | $149.62 | $2,868.26 | 0.00 | 0.00 | 5.21 |
| 6 | Redo in at least 3 years | 0.000% | $0.00 | $0.00 | $149.62 | $2,868.26 | 0.00 | 0.00 | 5.21 |
| 6 | comply | 0.000% | $0.00 | $0.00 | $149.62 | $2,868.26 | 0.00 | 0.00 | 5.21 |
| 6 | Not comply | 0.000% | $0.00 | $0.00 | $149.62 | $2,868.26 | 0.00 | 0.00 | 5.21 |
| 6 | No redo | 0.000% | $0.00 | $0.00 | $149.62 | $2,868.26 | 0.00 | 0.00 | 5.21 |
| 6 | Adenoma | 0.000% | $0.00 | $0.00 | $149.62 | $2,868.26 | 0.00 | 0.00 | 5.21 |
| 6 | Cancerous | 0.000% | $0.00 | $0.00 | $149.62 | $2,868.26 | 0.00 | 0.00 | 5.21 |
| 6 | Early | 0.000% | $0.00 | $0.00 | $149.62 | $2,868.26 | 0.00 | 0.00 | 5.21 |
| 6 | Advanced | 0.000% | $0.00 | $0.00 | $149.62 | $2,868.26 | 0.00 | 0.00 | 5.21 |
| 6 | Noncancerous | 0.000% | $0.00 | $0.00 | $149.62 | $2,868.26 | 0.00 | 0.00 | 5.21 |
| 6 | No adenoma | 0.000% | $0.00 | $0.00 | $149.62 | $2,868.26 | 0.00 | 0.00 | 5.21 |
| 7 | comply | 56.623% | $0.00 | $0.00 | $140.06 | $3,008.32 | 0.73 | 0.41 | 5.93 |
| 7 | Non-comply post colonoscopy | 0.493% | $0.00 | $0.00 | $140.06 | $3,008.32 | 0.00 | 0.00 | 5.93 |
| 7 | Early CRC | 2.729% | $3,597.93 | $98.20 | $140.06 | $3,008.32 | 0.60 | 0.02 | 5.93 |
| 7 | Advanced CRC | 0.127% | $4,846.03 | $6.16 | $140.06 | $3,008.32 | 0.43 | 0.00 | 5.93 |
| 7 | Die other causes | 36.636% | $0.00 | $0.00 | $140.06 | $3,008.32 | 0.74 | 0.27 | 5.93 |
| 7 | Colonoscopy with PureVu due added to inadequate prep or not comply | 3.036% | $609.82 | $18.52 | $140.06 | $3,008.32 | 0.74 | 0.02 | 5.93 |
| 7 | Adenoma surveillance | 0.000% | $2,480.91 | $0.00 | $140.06 | $3,008.32 | 0.74 | 0.00 | 5.93 |
| 7 | Non-compliance with system | 0.000% | $0.00 | $0.00 | $140.06 | $3,008.32 | 0.00 | 0.00 | 5.93 |
| 7 | Dead | 0.354% | $4,846.03 | $17.18 | $140.06 | $3,008.32 | 0.00 | 0.00 | 5.93 |
| 7 | Screening every 10 years - average risk | 0.000% | $0.00 | $0.00 | $140.06 | $3,008.32 | 0.00 | 0.00 | 5.93 |
| 7 | Adequate Prep | 0.000% | $0.00 | $0.00 | $140.06 | $3,008.32 | 0.00 | 0.00 | 5.93 |
| 7 | no adenoma screening | 0.000% | $0.00 | $0.00 | $140.06 | $3,008.32 | 0.00 | 0.00 | 5.93 |
| 7 | Colonoscopy TN | 0.000% | $0.00 | $0.00 | $140.06 | $3,008.32 | 0.00 | 0.00 | 5.93 |
| 7 | Comply | 0.000% | $0.00 | $0.00 | $140.06 | $3,008.32 | 0.00 | 0.00 | 5.93 |
| 7 | Non-comply | 0.000% | $0.00 | $0.00 | $140.06 | $3,008.32 | 0.00 | 0.00 | 5.93 |
| 7 | Colonoscopy FP | 0.000% | $0.00 | $0.00 | $140.06 | $3,008.32 | 0.00 | 0.00 | 5.93 |
| 7 | adenoma | 0.000% | $0.00 | $0.00 | $140.06 | $3,008.32 | 0.00 | 0.00 | 5.93 |
| 7 | Distal Colon (includes descending, sigmoid colon, splenic flexure, rectum) | 0.000% | $0.00 | $0.00 | $140.06 | $3,008.32 | 0.00 | 0.00 | 5.93 |
| 7 | Colonoscopy TP | 0.000% | $0.00 | $0.00 | $140.06 | $3,008.32 | 0.00 | 0.00 | 5.93 |
| 7 | Colonoscopy FN | 0.000% | $0.00 | $0.00 | $140.06 | $3,008.32 | 0.00 | 0.00 | 5.93 |
| 7 | cancer | 0.000% | $0.00 | $0.00 | $140.06 | $3,008.32 | 0.00 | 0.00 | 5.93 |
| 7 | early | 0.000% | $0.00 | $0.00 | $140.06 | $3,008.32 | 0.00 | 0.00 | 5.93 |
| 7 | late | 0.000% | $0.00 | $0.00 | $140.06 | $3,008.32 | 0.00 | 0.00 | 5.93 |
| 7 | no cancer | 0.000% | $0.00 | $0.00 | $140.06 | $3,008.32 | 0.00 | 0.00 | 5.93 |
| 7 | Comply | 0.000% | $0.00 | $0.00 | $140.06 | $3,008.32 | 0.00 | 0.00 | 5.93 |
| 7 | Non-comply | 0.000% | $0.00 | $0.00 | $140.06 | $3,008.32 | 0.00 | 0.00 | 5.93 |
| 7 | Proximal colon (includes ascending, transverse) | 0.000% | $0.00 | $0.00 | $140.06 | $3,008.32 | 0.00 | 0.00 | 5.93 |
| 7 | Colonoscopy TP | 0.000% | $0.00 | $0.00 | $140.06 | $3,008.32 | 0.00 | 0.00 | 5.93 |
| 7 | Colonoscopy FN | 0.000% | $0.00 | $0.00 | $140.06 | $3,008.32 | 0.00 | 0.00 | 5.93 |
| 7 | cancer | 0.000% | $0.00 | $0.00 | $140.06 | $3,008.32 | 0.00 | 0.00 | 5.93 |
| 7 | early | 0.000% | $0.00 | $0.00 | $140.06 | $3,008.32 | 0.00 | 0.00 | 5.93 |
| 7 | late | 0.000% | $0.00 | $0.00 | $140.06 | $3,008.32 | 0.00 | 0.00 | 5.93 |
| 7 | no cancer | 0.000% | $0.00 | $0.00 | $140.06 | $3,008.32 | 0.00 | 0.00 | 5.93 |
| 7 | Comply | 0.000% | $0.00 | $0.00 | $140.06 | $3,008.32 | 0.00 | 0.00 | 5.93 |
| 7 | Non-comply | 0.000% | $0.00 | $0.00 | $140.06 | $3,008.32 | 0.00 | 0.00 | 5.93 |
| 7 | Inadequate Prep | 0.000% | $0.00 | $0.00 | $140.06 | $3,008.32 | 0.00 | 0.00 | 5.93 |
| 7 | PureVu usage | 0.000% | $0.00 | $0.00 | $140.06 | $3,008.32 | 0.00 | 0.00 | 5.93 |
| 7 | Colonoscopy FN | 0.000% | $0.00 | $0.00 | $140.06 | $3,008.32 | 0.00 | 0.00 | 5.93 |
| 7 | cancer | 0.000% | $0.00 | $0.00 | $140.06 | $3,008.32 | 0.00 | 0.00 | 5.93 |
| 7 | early | 0.000% | $0.00 | $0.00 | $140.06 | $3,008.32 | 0.00 | 0.00 | 5.93 |
| 7 | late | 0.000% | $0.00 | $0.00 | $140.06 | $3,008.32 | 0.00 | 0.00 | 5.93 |
| 7 | no cancer | 0.000% | $0.00 | $0.00 | $140.06 | $3,008.32 | 0.00 | 0.00 | 5.93 |
| 7 | Comply | 0.000% | $0.00 | $0.00 | $140.06 | $3,008.32 | 0.00 | 0.00 | 5.93 |
| 7 | Non-comply | 0.000% | $0.00 | $0.00 | $140.06 | $3,008.32 | 0.00 | 0.00 | 5.93 |
| 7 | No screening | 56.623% | $0.00 | $0.00 | $140.06 | $3,008.32 | 0.00 | 0.00 | 5.93 |
| 7 | Adenoma | 0.214% | $0.00 | $0.00 | $140.06 | $3,008.32 | 0.00 | 0.00 | 5.93 |
| 7 | Cancerous | 0.001% | $0.00 | $0.00 | $140.06 | $3,008.32 | 0.00 | 0.00 | 5.93 |
| 7 | Early | 0.001% | $0.00 | $0.00 | $140.06 | $3,008.32 | 0.00 | 0.00 | 5.93 |
| 7 | Advanced | 0.000% | $0.00 | $0.00 | $140.06 | $3,008.32 | 0.00 | 0.00 | 5.93 |
| 7 | Noncancerous | 0.213% | $0.00 | $0.00 | $140.06 | $3,008.32 | 0.00 | 0.00 | 5.93 |
| 7 | comply | 0.151% | $0.00 | $0.00 | $140.06 | $3,008.32 | 0.00 | 0.00 | 5.93 |
| 7 | Not comply | 0.062% | $0.00 | $0.00 | $140.06 | $3,008.32 | 0.00 | 0.00 | 5.93 |
| 7 | No adenoma | 0.279% | $0.00 | $0.00 | $140.06 | $3,008.32 | 0.00 | 0.00 | 5.93 |
| 7 | comply | 0.169% | $0.00 | $0.00 | $140.06 | $3,008.32 | 0.00 | 0.00 | 5.93 |
| 7 | Not comply | 0.111% | $0.00 | $0.00 | $140.06 | $3,008.32 | 0.00 | 0.00 | 5.93 |
| 7 | Continue | 0.102% | $0.00 | $0.00 | $140.06 | $3,008.32 | 0.00 | 0.00 | 5.93 |
| 7 | Die | 0.025% | $0.00 | $0.00 | $140.06 | $3,008.32 | 0.00 | 0.00 | 5.93 |
| 7 | PureVu usage | 2.368% | $0.00 | $0.00 | $140.06 | $3,008.32 | 0.00 | 0.00 | 5.93 |
| 7 | Later followup | 0.668% | $0.00 | $0.00 | $140.06 | $3,008.32 | 0.00 | 0.00 | 5.93 |
| 7 | Redo 2 years | 0.000% | $0.00 | $0.00 | $140.06 | $3,008.32 | 0.00 | 0.00 | 5.93 |
| 7 | comply | 0.000% | $0.00 | $0.00 | $140.06 | $3,008.32 | 0.00 | 0.00 | 5.93 |
| 7 | Not comply | 0.000% | $0.00 | $0.00 | $140.06 | $3,008.32 | 0.00 | 0.00 | 5.93 |
| 7 | No redo | 0.668% | $0.00 | $0.00 | $140.06 | $3,008.32 | 0.00 | 0.00 | 5.93 |
| 7 | Redo in at least 3 years | 0.000% | $0.00 | $0.00 | $140.06 | $3,008.32 | 0.00 | 0.00 | 5.93 |
| 7 | comply | 0.000% | $0.00 | $0.00 | $140.06 | $3,008.32 | 0.00 | 0.00 | 5.93 |
| 7 | Not comply | 0.000% | $0.00 | $0.00 | $140.06 | $3,008.32 | 0.00 | 0.00 | 5.93 |
| 7 | No redo | 0.000% | $0.00 | $0.00 | $140.06 | $3,008.32 | 0.00 | 0.00 | 5.93 |
| 7 | Adenoma | 0.000% | $0.00 | $0.00 | $140.06 | $3,008.32 | 0.00 | 0.00 | 5.93 |
| 7 | Cancerous | 0.000% | $0.00 | $0.00 | $140.06 | $3,008.32 | 0.00 | 0.00 | 5.93 |
| 7 | Early | 0.000% | $0.00 | $0.00 | $140.06 | $3,008.32 | 0.00 | 0.00 | 5.93 |
| 7 | Advanced | 0.000% | $0.00 | $0.00 | $140.06 | $3,008.32 | 0.00 | 0.00 | 5.93 |
| 7 | Noncancerous | 0.000% | $0.00 | $0.00 | $140.06 | $3,008.32 | 0.00 | 0.00 | 5.93 |
| 7 | No adenoma | 0.000% | $0.00 | $0.00 | $140.06 | $3,008.32 | 0.00 | 0.00 | 5.93 |
| 8 | comply | 57.611% | $0.00 | $0.00 | $132.06 | $3,140.37 | 0.71 | 0.41 | 6.64 |
| 8 | Non-comply post colonoscopy | 0.173% | $0.00 | $0.00 | $132.06 | $3,140.37 | 0.00 | 0.00 | 6.64 |
| 8 | Early CRC | 2.730% | $3,493.14 | $95.37 | $132.06 | $3,140.37 | 0.58 | 0.02 | 6.64 |
| 8 | Advanced CRC | 0.102% | $4,704.88 | $4.79 | $132.06 | $3,140.37 | 0.42 | 0.00 | 6.64 |
| 8 | Die other causes | 36.636% | $0.00 | $0.00 | $132.06 | $3,140.37 | 0.72 | 0.26 | 6.64 |
| 8 | Colonoscopy with PureVu due added to inadequate prep or not comply | 2.368% | $592.06 | $14.02 | $132.06 | $3,140.37 | 0.72 | 0.02 | 6.64 |
| 8 | Adenoma surveillance | 0.000% | $2,408.65 | $0.00 | $132.06 | $3,140.37 | 0.72 | 0.00 | 6.64 |
| 8 | Non-compliance with system | 0.000% | $0.00 | $0.00 | $132.06 | $3,140.37 | 0.00 | 0.00 | 6.64 |
| 8 | Dead | 0.380% | $4,704.88 | $17.87 | $132.06 | $3,140.37 | 0.00 | 0.00 | 6.64 |
| 8 | Screening every 10 years - average risk | 0.000% | $0.00 | $0.00 | $132.06 | $3,140.37 | 0.00 | 0.00 | 6.64 |
| 8 | Adequate Prep | 0.000% | $0.00 | $0.00 | $132.06 | $3,140.37 | 0.00 | 0.00 | 6.64 |
| 8 | no adenoma screening | 0.000% | $0.00 | $0.00 | $132.06 | $3,140.37 | 0.00 | 0.00 | 6.64 |
| 8 | Colonoscopy TN | 0.000% | $0.00 | $0.00 | $132.06 | $3,140.37 | 0.00 | 0.00 | 6.64 |
| 8 | Comply | 0.000% | $0.00 | $0.00 | $132.06 | $3,140.37 | 0.00 | 0.00 | 6.64 |
| 8 | Non-comply | 0.000% | $0.00 | $0.00 | $132.06 | $3,140.37 | 0.00 | 0.00 | 6.64 |
| 8 | Colonoscopy FP | 0.000% | $0.00 | $0.00 | $132.06 | $3,140.37 | 0.00 | 0.00 | 6.64 |
| 8 | adenoma | 0.000% | $0.00 | $0.00 | $132.06 | $3,140.37 | 0.00 | 0.00 | 6.64 |
| 8 | Distal Colon (includes descending, sigmoid colon, splenic flexure, rectum) | 0.000% | $0.00 | $0.00 | $132.06 | $3,140.37 | 0.00 | 0.00 | 6.64 |
| 8 | Colonoscopy TP | 0.000% | $0.00 | $0.00 | $132.06 | $3,140.37 | 0.00 | 0.00 | 6.64 |
| 8 | Colonoscopy FN | 0.000% | $0.00 | $0.00 | $132.06 | $3,140.37 | 0.00 | 0.00 | 6.64 |
| 8 | cancer | 0.000% | $0.00 | $0.00 | $132.06 | $3,140.37 | 0.00 | 0.00 | 6.64 |
| 8 | early | 0.000% | $0.00 | $0.00 | $132.06 | $3,140.37 | 0.00 | 0.00 | 6.64 |
| 8 | late | 0.000% | $0.00 | $0.00 | $132.06 | $3,140.37 | 0.00 | 0.00 | 6.64 |
| 8 | no cancer | 0.000% | $0.00 | $0.00 | $132.06 | $3,140.37 | 0.00 | 0.00 | 6.64 |
| 8 | Comply | 0.000% | $0.00 | $0.00 | $132.06 | $3,140.37 | 0.00 | 0.00 | 6.64 |
| 8 | Non-comply | 0.000% | $0.00 | $0.00 | $132.06 | $3,140.37 | 0.00 | 0.00 | 6.64 |
| 8 | Proximal colon (includes ascending, transverse) | 0.000% | $0.00 | $0.00 | $132.06 | $3,140.37 | 0.00 | 0.00 | 6.64 |
| 8 | Colonoscopy TP | 0.000% | $0.00 | $0.00 | $132.06 | $3,140.37 | 0.00 | 0.00 | 6.64 |
| 8 | Colonoscopy FN | 0.000% | $0.00 | $0.00 | $132.06 | $3,140.37 | 0.00 | 0.00 | 6.64 |
| 8 | cancer | 0.000% | $0.00 | $0.00 | $132.06 | $3,140.37 | 0.00 | 0.00 | 6.64 |
| 8 | early | 0.000% | $0.00 | $0.00 | $132.06 | $3,140.37 | 0.00 | 0.00 | 6.64 |
| 8 | late | 0.000% | $0.00 | $0.00 | $132.06 | $3,140.37 | 0.00 | 0.00 | 6.64 |
| 8 | no cancer | 0.000% | $0.00 | $0.00 | $132.06 | $3,140.37 | 0.00 | 0.00 | 6.64 |
| 8 | Comply | 0.000% | $0.00 | $0.00 | $132.06 | $3,140.37 | 0.00 | 0.00 | 6.64 |
| 8 | Non-comply | 0.000% | $0.00 | $0.00 | $132.06 | $3,140.37 | 0.00 | 0.00 | 6.64 |
| 8 | Inadequate Prep | 0.000% | $0.00 | $0.00 | $132.06 | $3,140.37 | 0.00 | 0.00 | 6.64 |
| 8 | PureVu usage | 0.000% | $0.00 | $0.00 | $132.06 | $3,140.37 | 0.00 | 0.00 | 6.64 |
| 8 | Colonoscopy FN | 0.000% | $0.00 | $0.00 | $132.06 | $3,140.37 | 0.00 | 0.00 | 6.64 |
| 8 | cancer | 0.000% | $0.00 | $0.00 | $132.06 | $3,140.37 | 0.00 | 0.00 | 6.64 |
| 8 | early | 0.000% | $0.00 | $0.00 | $132.06 | $3,140.37 | 0.00 | 0.00 | 6.64 |
| 8 | late | 0.000% | $0.00 | $0.00 | $132.06 | $3,140.37 | 0.00 | 0.00 | 6.64 |
| 8 | no cancer | 0.000% | $0.00 | $0.00 | $132.06 | $3,140.37 | 0.00 | 0.00 | 6.64 |
| 8 | Comply | 0.000% | $0.00 | $0.00 | $132.06 | $3,140.37 | 0.00 | 0.00 | 6.64 |
| 8 | Non-comply | 0.000% | $0.00 | $0.00 | $132.06 | $3,140.37 | 0.00 | 0.00 | 6.64 |
| 8 | No screening | 57.611% | $0.00 | $0.00 | $132.06 | $3,140.37 | 0.00 | 0.00 | 6.64 |
| 8 | Adenoma | 0.075% | $0.00 | $0.00 | $132.06 | $3,140.37 | 0.00 | 0.00 | 6.64 |
| 8 | Cancerous | 0.000% | $0.00 | $0.00 | $132.06 | $3,140.37 | 0.00 | 0.00 | 6.64 |
| 8 | Early | 0.000% | $0.00 | $0.00 | $132.06 | $3,140.37 | 0.00 | 0.00 | 6.64 |
| 8 | Advanced | 0.000% | $0.00 | $0.00 | $132.06 | $3,140.37 | 0.00 | 0.00 | 6.64 |
| 8 | Noncancerous | 0.075% | $0.00 | $0.00 | $132.06 | $3,140.37 | 0.00 | 0.00 | 6.64 |
| 8 | comply | 0.053% | $0.00 | $0.00 | $132.06 | $3,140.37 | 0.00 | 0.00 | 6.64 |
| 8 | Not comply | 0.022% | $0.00 | $0.00 | $132.06 | $3,140.37 | 0.00 | 0.00 | 6.64 |
| 8 | No adenoma | 0.098% | $0.00 | $0.00 | $132.06 | $3,140.37 | 0.00 | 0.00 | 6.64 |
| 8 | comply | 0.059% | $0.00 | $0.00 | $132.06 | $3,140.37 | 0.00 | 0.00 | 6.64 |
| 8 | Not comply | 0.039% | $0.00 | $0.00 | $132.06 | $3,140.37 | 0.00 | 0.00 | 6.64 |
| 8 | Continue | 0.081% | $0.00 | $0.00 | $132.06 | $3,140.37 | 0.00 | 0.00 | 6.64 |
| 8 | Die | 0.020% | $0.00 | $0.00 | $132.06 | $3,140.37 | 0.00 | 0.00 | 6.64 |
| 8 | PureVu usage | 1.847% | $0.00 | $0.00 | $132.06 | $3,140.37 | 0.00 | 0.00 | 6.64 |
| 8 | Later followup | 0.521% | $0.00 | $0.00 | $132.06 | $3,140.37 | 0.00 | 0.00 | 6.64 |
| 8 | Redo 2 years | 0.521% | $0.00 | $0.00 | $132.06 | $3,140.37 | 0.00 | 0.00 | 6.64 |
| 8 | comply | 0.291% | $0.00 | $0.00 | $132.06 | $3,140.37 | 0.00 | 0.00 | 6.64 |
| 8 | Not comply | 0.230% | $0.00 | $0.00 | $132.06 | $3,140.37 | 0.00 | 0.00 | 6.64 |
| 8 | No redo | 0.000% | $0.00 | $0.00 | $132.06 | $3,140.37 | 0.00 | 0.00 | 6.64 |
| 8 | Redo in at least 3 years | 0.000% | $0.00 | $0.00 | $132.06 | $3,140.37 | 0.00 | 0.00 | 6.64 |
| 8 | comply | 0.000% | $0.00 | $0.00 | $132.06 | $3,140.37 | 0.00 | 0.00 | 6.64 |
| 8 | Not comply | 0.000% | $0.00 | $0.00 | $132.06 | $3,140.37 | 0.00 | 0.00 | 6.64 |
| 8 | No redo | 0.000% | $0.00 | $0.00 | $132.06 | $3,140.37 | 0.00 | 0.00 | 6.64 |
| 8 | Adenoma | 0.000% | $0.00 | $0.00 | $132.06 | $3,140.37 | 0.00 | 0.00 | 6.64 |
| 8 | Cancerous | 0.000% | $0.00 | $0.00 | $132.06 | $3,140.37 | 0.00 | 0.00 | 6.64 |
| 8 | Early | 0.000% | $0.00 | $0.00 | $132.06 | $3,140.37 | 0.00 | 0.00 | 6.64 |
| 8 | Advanced | 0.000% | $0.00 | $0.00 | $132.06 | $3,140.37 | 0.00 | 0.00 | 6.64 |
| 8 | Noncancerous | 0.000% | $0.00 | $0.00 | $132.06 | $3,140.37 | 0.00 | 0.00 | 6.64 |
| 8 | No adenoma | 0.000% | $0.00 | $0.00 | $132.06 | $3,140.37 | 0.00 | 0.00 | 6.64 |
| 9 | comply | 58.014% | $0.00 | $0.00 | $125.23 | $3,265.60 | 0.69 | 0.40 | 7.32 |
| 9 | Non-comply post colonoscopy | 0.290% | $0.00 | $0.00 | $125.23 | $3,265.60 | 0.00 | 0.00 | 7.32 |
| 9 | Early CRC | 2.730% | $3,391.39 | $92.60 | $125.23 | $3,265.60 | 0.57 | 0.02 | 7.32 |
| 9 | Advanced CRC | 0.082% | $4,567.84 | $3.72 | $125.23 | $3,265.60 | 0.40 | 0.00 | 7.32 |
| 9 | Die other causes | 36.636% | $0.00 | $0.00 | $125.23 | $3,265.60 | 0.70 | 0.25 | 7.32 |
| 9 | Colonoscopy with PureVu due added to inadequate prep or not comply | 1.847% | $574.81 | $10.62 | $125.23 | $3,265.60 | 0.70 | 0.01 | 7.32 |
| 9 | Adenoma surveillance | 0.000% | $2,338.50 | $0.00 | $125.23 | $3,265.60 | 0.70 | 0.00 | 7.32 |
| 9 | Non-compliance with system | 0.000% | $0.00 | $0.00 | $125.23 | $3,265.60 | 0.00 | 0.00 | 7.32 |
| 9 | Dead | 0.400% | $4,567.84 | $18.28 | $125.23 | $3,265.60 | 0.00 | 0.00 | 7.32 |
| 9 | Screening every 10 years - average risk | 0.000% | $0.00 | $0.00 | $125.23 | $3,265.60 | 0.00 | 0.00 | 7.32 |
| 9 | Adequate Prep | 0.000% | $0.00 | $0.00 | $125.23 | $3,265.60 | 0.00 | 0.00 | 7.32 |
| 9 | no adenoma screening | 0.000% | $0.00 | $0.00 | $125.23 | $3,265.60 | 0.00 | 0.00 | 7.32 |
| 9 | Colonoscopy TN | 0.000% | $0.00 | $0.00 | $125.23 | $3,265.60 | 0.00 | 0.00 | 7.32 |
| 9 | Comply | 0.000% | $0.00 | $0.00 | $125.23 | $3,265.60 | 0.00 | 0.00 | 7.32 |
| 9 | Non-comply | 0.000% | $0.00 | $0.00 | $125.23 | $3,265.60 | 0.00 | 0.00 | 7.32 |
| 9 | Colonoscopy FP | 0.000% | $0.00 | $0.00 | $125.23 | $3,265.60 | 0.00 | 0.00 | 7.32 |
| 9 | adenoma | 0.000% | $0.00 | $0.00 | $125.23 | $3,265.60 | 0.00 | 0.00 | 7.32 |
| 9 | Distal Colon (includes descending, sigmoid colon, splenic flexure, rectum) | 0.000% | $0.00 | $0.00 | $125.23 | $3,265.60 | 0.00 | 0.00 | 7.32 |
| 9 | Colonoscopy TP | 0.000% | $0.00 | $0.00 | $125.23 | $3,265.60 | 0.00 | 0.00 | 7.32 |
| 9 | Colonoscopy FN | 0.000% | $0.00 | $0.00 | $125.23 | $3,265.60 | 0.00 | 0.00 | 7.32 |
| 9 | cancer | 0.000% | $0.00 | $0.00 | $125.23 | $3,265.60 | 0.00 | 0.00 | 7.32 |
| 9 | early | 0.000% | $0.00 | $0.00 | $125.23 | $3,265.60 | 0.00 | 0.00 | 7.32 |
| 9 | late | 0.000% | $0.00 | $0.00 | $125.23 | $3,265.60 | 0.00 | 0.00 | 7.32 |
| 9 | no cancer | 0.000% | $0.00 | $0.00 | $125.23 | $3,265.60 | 0.00 | 0.00 | 7.32 |
| 9 | Comply | 0.000% | $0.00 | $0.00 | $125.23 | $3,265.60 | 0.00 | 0.00 | 7.32 |
| 9 | Non-comply | 0.000% | $0.00 | $0.00 | $125.23 | $3,265.60 | 0.00 | 0.00 | 7.32 |
| 9 | Proximal colon (includes ascending, transverse) | 0.000% | $0.00 | $0.00 | $125.23 | $3,265.60 | 0.00 | 0.00 | 7.32 |
| 9 | Colonoscopy TP | 0.000% | $0.00 | $0.00 | $125.23 | $3,265.60 | 0.00 | 0.00 | 7.32 |
| 9 | Colonoscopy FN | 0.000% | $0.00 | $0.00 | $125.23 | $3,265.60 | 0.00 | 0.00 | 7.32 |
| 9 | cancer | 0.000% | $0.00 | $0.00 | $125.23 | $3,265.60 | 0.00 | 0.00 | 7.32 |
| 9 | early | 0.000% | $0.00 | $0.00 | $125.23 | $3,265.60 | 0.00 | 0.00 | 7.32 |
| 9 | late | 0.000% | $0.00 | $0.00 | $125.23 | $3,265.60 | 0.00 | 0.00 | 7.32 |
| 9 | no cancer | 0.000% | $0.00 | $0.00 | $125.23 | $3,265.60 | 0.00 | 0.00 | 7.32 |
| 9 | Comply | 0.000% | $0.00 | $0.00 | $125.23 | $3,265.60 | 0.00 | 0.00 | 7.32 |
| 9 | Non-comply | 0.000% | $0.00 | $0.00 | $125.23 | $3,265.60 | 0.00 | 0.00 | 7.32 |
| 9 | Inadequate Prep | 0.000% | $0.00 | $0.00 | $125.23 | $3,265.60 | 0.00 | 0.00 | 7.32 |
| 9 | PureVu usage | 0.000% | $0.00 | $0.00 | $125.23 | $3,265.60 | 0.00 | 0.00 | 7.32 |
| 9 | Colonoscopy FN | 0.000% | $0.00 | $0.00 | $125.23 | $3,265.60 | 0.00 | 0.00 | 7.32 |
| 9 | cancer | 0.000% | $0.00 | $0.00 | $125.23 | $3,265.60 | 0.00 | 0.00 | 7.32 |
| 9 | early | 0.000% | $0.00 | $0.00 | $125.23 | $3,265.60 | 0.00 | 0.00 | 7.32 |
| 9 | late | 0.000% | $0.00 | $0.00 | $125.23 | $3,265.60 | 0.00 | 0.00 | 7.32 |
| 9 | no cancer | 0.000% | $0.00 | $0.00 | $125.23 | $3,265.60 | 0.00 | 0.00 | 7.32 |
| 9 | Comply | 0.000% | $0.00 | $0.00 | $125.23 | $3,265.60 | 0.00 | 0.00 | 7.32 |
| 9 | Non-comply | 0.000% | $0.00 | $0.00 | $125.23 | $3,265.60 | 0.00 | 0.00 | 7.32 |
| 9 | No screening | 58.014% | $0.00 | $0.00 | $125.23 | $3,265.60 | 0.00 | 0.00 | 7.32 |
| 9 | Adenoma | 0.126% | $0.00 | $0.00 | $125.23 | $3,265.60 | 0.00 | 0.00 | 7.32 |
| 9 | Cancerous | 0.001% | $0.00 | $0.00 | $125.23 | $3,265.60 | 0.00 | 0.00 | 7.32 |
| 9 | Early | 0.000% | $0.00 | $0.00 | $125.23 | $3,265.60 | 0.00 | 0.00 | 7.32 |
| 9 | Advanced | 0.000% | $0.00 | $0.00 | $125.23 | $3,265.60 | 0.00 | 0.00 | 7.32 |
| 9 | Noncancerous | 0.125% | $0.00 | $0.00 | $125.23 | $3,265.60 | 0.00 | 0.00 | 7.32 |
| 9 | comply | 0.089% | $0.00 | $0.00 | $125.23 | $3,265.60 | 0.00 | 0.00 | 7.32 |
| 9 | Not comply | 0.036% | $0.00 | $0.00 | $125.23 | $3,265.60 | 0.00 | 0.00 | 7.32 |
| 9 | No adenoma | 0.164% | $0.00 | $0.00 | $125.23 | $3,265.60 | 0.00 | 0.00 | 7.32 |
| 9 | comply | 0.099% | $0.00 | $0.00 | $125.23 | $3,265.60 | 0.00 | 0.00 | 7.32 |
| 9 | Not comply | 0.065% | $0.00 | $0.00 | $125.23 | $3,265.60 | 0.00 | 0.00 | 7.32 |
| 9 | Continue | 0.065% | $0.00 | $0.00 | $125.23 | $3,265.60 | 0.00 | 0.00 | 7.32 |
| 9 | Die | 0.016% | $0.00 | $0.00 | $125.23 | $3,265.60 | 0.00 | 0.00 | 7.32 |
| 9 | PureVu usage | 1.441% | $0.00 | $0.00 | $125.23 | $3,265.60 | 0.00 | 0.00 | 7.32 |
| 9 | Later followup | 0.406% | $0.00 | $0.00 | $125.23 | $3,265.60 | 0.00 | 0.00 | 7.32 |
| 9 | Redo 2 years | 0.000% | $0.00 | $0.00 | $125.23 | $3,265.60 | 0.00 | 0.00 | 7.32 |
| 9 | comply | 0.000% | $0.00 | $0.00 | $125.23 | $3,265.60 | 0.00 | 0.00 | 7.32 |
| 9 | Not comply | 0.000% | $0.00 | $0.00 | $125.23 | $3,265.60 | 0.00 | 0.00 | 7.32 |
| 9 | No redo | 0.406% | $0.00 | $0.00 | $125.23 | $3,265.60 | 0.00 | 0.00 | 7.32 |
| 9 | Redo in at least 3 years | 0.000% | $0.00 | $0.00 | $125.23 | $3,265.60 | 0.00 | 0.00 | 7.32 |
| 9 | comply | 0.000% | $0.00 | $0.00 | $125.23 | $3,265.60 | 0.00 | 0.00 | 7.32 |
| 9 | Not comply | 0.000% | $0.00 | $0.00 | $125.23 | $3,265.60 | 0.00 | 0.00 | 7.32 |
| 9 | No redo | 0.000% | $0.00 | $0.00 | $125.23 | $3,265.60 | 0.00 | 0.00 | 7.32 |
| 9 | Adenoma | 0.000% | $0.00 | $0.00 | $125.23 | $3,265.60 | 0.00 | 0.00 | 7.32 |
| 9 | Cancerous | 0.000% | $0.00 | $0.00 | $125.23 | $3,265.60 | 0.00 | 0.00 | 7.32 |
| 9 | Early | 0.000% | $0.00 | $0.00 | $125.23 | $3,265.60 | 0.00 | 0.00 | 7.32 |
| 9 | Advanced | 0.000% | $0.00 | $0.00 | $125.23 | $3,265.60 | 0.00 | 0.00 | 7.32 |
| 9 | Noncancerous | 0.000% | $0.00 | $0.00 | $125.23 | $3,265.60 | 0.00 | 0.00 | 7.32 |
| 9 | No adenoma | 0.000% | $0.00 | $0.00 | $125.23 | $3,265.60 | 0.00 | 0.00 | 7.32 |
| 10 | comply | 58.609% | $1,638.74 | $960.44 | $1,079.77 | $4,345.37 | 0.67 | 0.39 | 7.98 |
| 10 | Non-comply post colonoscopy | 0.102% | $0.00 | $0.00 | $1,079.77 | $4,345.37 | 0.00 | 0.00 | 7.98 |
| 10 | Early CRC | 2.731% | $3,292.62 | $89.92 | $1,079.77 | $4,345.37 | 0.55 | 0.02 | 7.98 |
| 10 | Advanced CRC | 0.065% | $4,434.80 | $2.90 | $1,079.77 | $4,345.37 | 0.39 | 0.00 | 7.98 |
| 10 | Die other causes | 36.636% | $0.00 | $0.00 | $1,079.77 | $4,345.37 | 0.67 | 0.25 | 7.98 |
| 10 | Colonoscopy with PureVu due added to inadequate prep or not comply | 1.441% | $558.07 | $8.04 | $1,079.77 | $4,345.37 | 0.68 | 0.01 | 7.98 |
| 10 | Adenoma surveillance | 0.000% | $2,270.38 | $0.00 | $1,079.77 | $4,345.37 | 0.68 | 0.00 | 7.98 |
| 10 | Non-compliance with system | 0.000% | $0.00 | $0.00 | $1,079.77 | $4,345.37 | 0.00 | 0.00 | 7.98 |
| 10 | Dead | 0.417% | $4,434.80 | $18.48 | $1,079.77 | $4,345.37 | 0.00 | 0.00 | 7.98 |
| 10 | Screening every 10 years - average risk | 58.609% | $0.00 | $0.00 | $1,079.77 | $4,345.37 | 0.00 | 0.00 | 7.98 |
| 10 | Adequate Prep | 41.807% | $0.00 | $0.00 | $1,079.77 | $4,345.37 | 0.00 | 0.00 | 7.98 |
| 10 | no adenoma screening | 29.265% | $0.00 | $0.00 | $1,079.77 | $4,345.37 | 0.00 | 0.00 | 7.98 |
| 10 | Colonoscopy TN | 27.246% | $0.00 | $0.00 | $1,079.77 | $4,345.37 | 0.00 | 0.00 | 7.98 |
| 10 | Comply | 16.429% | $0.00 | $0.00 | $1,079.77 | $4,345.37 | 0.00 | 0.00 | 7.98 |
| 10 | Non-comply | 10.817% | $0.00 | $0.00 | $1,079.77 | $4,345.37 | 0.00 | 0.00 | 7.98 |
| 10 | Colonoscopy FP | 2.019% | $0.00 | $0.00 | $1,079.77 | $4,345.37 | 0.00 | 0.00 | 7.98 |
| 10 | adenoma | 12.542% | $0.00 | $0.00 | $1,079.77 | $4,345.37 | 0.00 | 0.00 | 7.98 |
| 10 | Distal Colon (includes descending, sigmoid colon, splenic flexure, rectum) | 6.447% | $0.00 | $0.00 | $1,079.77 | $4,345.37 | 0.00 | 0.00 | 7.98 |
| 10 | Colonoscopy TP | 5.802% | $0.00 | $0.00 | $1,079.77 | $4,345.37 | 0.00 | 0.00 | 7.98 |
| 10 | Colonoscopy FN | 0.645% | $0.00 | $0.00 | $1,079.77 | $4,345.37 | 0.00 | 0.00 | 7.98 |
| 10 | cancer | 0.003% | $0.00 | $0.00 | $1,079.77 | $4,345.37 | 0.00 | 0.00 | 7.98 |
| 10 | early | 0.002% | $0.00 | $0.00 | $1,079.77 | $4,345.37 | 0.00 | 0.00 | 7.98 |
| 10 | late | 0.000% | $0.00 | $0.00 | $1,079.77 | $4,345.37 | 0.00 | 0.00 | 7.98 |
| 10 | no cancer | 0.642% | $0.00 | $0.00 | $1,079.77 | $4,345.37 | 0.00 | 0.00 | 7.98 |
| 10 | Comply | 0.387% | $0.00 | $0.00 | $1,079.77 | $4,345.37 | 0.00 | 0.00 | 7.98 |
| 10 | Non-comply | 0.255% | $0.00 | $0.00 | $1,079.77 | $4,345.37 | 0.00 | 0.00 | 7.98 |
| 10 | Proximal colon (includes ascending, transverse) | 6.096% | $0.00 | $0.00 | $1,079.77 | $4,345.37 | 0.00 | 0.00 | 7.98 |
| 10 | Colonoscopy TP | 5.486% | $0.00 | $0.00 | $1,079.77 | $4,345.37 | 0.00 | 0.00 | 7.98 |
| 10 | Colonoscopy FN | 0.610% | $0.00 | $0.00 | $1,079.77 | $4,345.37 | 0.00 | 0.00 | 7.98 |
| 10 | cancer | 0.003% | $0.00 | $0.00 | $1,079.77 | $4,345.37 | 0.00 | 0.00 | 7.98 |
| 10 | early | 0.002% | $0.00 | $0.00 | $1,079.77 | $4,345.37 | 0.00 | 0.00 | 7.98 |
| 10 | late | 0.000% | $0.00 | $0.00 | $1,079.77 | $4,345.37 | 0.00 | 0.00 | 7.98 |
| 10 | no cancer | 0.607% | $0.00 | $0.00 | $1,079.77 | $4,345.37 | 0.00 | 0.00 | 7.98 |
| 10 | Comply | 0.366% | $0.00 | $0.00 | $1,079.77 | $4,345.37 | 0.00 | 0.00 | 7.98 |
| 10 | Non-comply | 0.241% | $0.00 | $0.00 | $1,079.77 | $4,345.37 | 0.00 | 0.00 | 7.98 |
| 10 | Inadequate Prep | 16.801% | $0.00 | $0.00 | $1,079.77 | $4,345.37 | 0.00 | 0.00 | 7.98 |
| 10 | PureVu usage | 13.105% | $0.00 | $0.00 | $1,079.77 | $4,345.37 | 0.00 | 0.00 | 7.98 |
| 10 | Colonoscopy FN | 3.696% | $0.00 | $0.00 | $1,079.77 | $4,345.37 | 0.00 | 0.00 | 7.98 |
| 10 | cancer | 0.097% | $0.00 | $0.00 | $1,079.77 | $4,345.37 | 0.00 | 0.00 | 7.98 |
| 10 | early | 0.083% | $0.00 | $0.00 | $1,079.77 | $4,345.37 | 0.00 | 0.00 | 7.98 |
| 10 | late | 0.015% | $0.00 | $0.00 | $1,079.77 | $4,345.37 | 0.00 | 0.00 | 7.98 |
| 10 | no cancer | 3.599% | $0.00 | $0.00 | $1,079.77 | $4,345.37 | 0.00 | 0.00 | 7.98 |
| 10 | Comply | 2.170% | $0.00 | $0.00 | $1,079.77 | $4,345.37 | 0.00 | 0.00 | 7.98 |
| 10 | Non-comply | 1.429% | $0.00 | $0.00 | $1,079.77 | $4,345.37 | 0.00 | 0.00 | 7.98 |
| 10 | No screening | 0.000% | $0.00 | $0.00 | $1,079.77 | $4,345.37 | 0.00 | 0.00 | 7.98 |
| 10 | Adenoma | 0.044% | $0.00 | $0.00 | $1,079.77 | $4,345.37 | 0.00 | 0.00 | 7.98 |
| 10 | Cancerous | 0.000% | $0.00 | $0.00 | $1,079.77 | $4,345.37 | 0.00 | 0.00 | 7.98 |
| 10 | Early | 0.000% | $0.00 | $0.00 | $1,079.77 | $4,345.37 | 0.00 | 0.00 | 7.98 |
| 10 | Advanced | 0.000% | $0.00 | $0.00 | $1,079.77 | $4,345.37 | 0.00 | 0.00 | 7.98 |
| 10 | Noncancerous | 0.044% | $0.00 | $0.00 | $1,079.77 | $4,345.37 | 0.00 | 0.00 | 7.98 |
| 10 | comply | 0.031% | $0.00 | $0.00 | $1,079.77 | $4,345.37 | 0.00 | 0.00 | 7.98 |
| 10 | Not comply | 0.013% | $0.00 | $0.00 | $1,079.77 | $4,345.37 | 0.00 | 0.00 | 7.98 |
| 10 | No adenoma | 0.058% | $0.00 | $0.00 | $1,079.77 | $4,345.37 | 0.00 | 0.00 | 7.98 |
| 10 | comply | 0.035% | $0.00 | $0.00 | $1,079.77 | $4,345.37 | 0.00 | 0.00 | 7.98 |
| 10 | Not comply | 0.023% | $0.00 | $0.00 | $1,079.77 | $4,345.37 | 0.00 | 0.00 | 7.98 |
| 10 | Continue | 0.052% | $0.00 | $0.00 | $1,079.77 | $4,345.37 | 0.00 | 0.00 | 7.98 |
| 10 | Die | 0.013% | $0.00 | $0.00 | $1,079.77 | $4,345.37 | 0.00 | 0.00 | 7.98 |
| 10 | PureVu usage | 1.124% | $0.00 | $0.00 | $1,079.77 | $4,345.37 | 0.00 | 0.00 | 7.98 |
| 10 | Later followup | 0.317% | $0.00 | $0.00 | $1,079.77 | $4,345.37 | 0.00 | 0.00 | 7.98 |
| 10 | Redo 2 years | 0.317% | $0.00 | $0.00 | $1,079.77 | $4,345.37 | 0.00 | 0.00 | 7.98 |
| 10 | comply | 0.177% | $0.00 | $0.00 | $1,079.77 | $4,345.37 | 0.00 | 0.00 | 7.98 |
| 10 | Not comply | 0.140% | $0.00 | $0.00 | $1,079.77 | $4,345.37 | 0.00 | 0.00 | 7.98 |
| 10 | No redo | 0.000% | $0.00 | $0.00 | $1,079.77 | $4,345.37 | 0.00 | 0.00 | 7.98 |
| 10 | Redo in at least 3 years | 0.000% | $0.00 | $0.00 | $1,079.77 | $4,345.37 | 0.00 | 0.00 | 7.98 |
| 10 | comply | 0.000% | $0.00 | $0.00 | $1,079.77 | $4,345.37 | 0.00 | 0.00 | 7.98 |
| 10 | Not comply | 0.000% | $0.00 | $0.00 | $1,079.77 | $4,345.37 | 0.00 | 0.00 | 7.98 |
| 10 | No redo | 0.000% | $0.00 | $0.00 | $1,079.77 | $4,345.37 | 0.00 | 0.00 | 7.98 |
| 10 | Adenoma | 0.000% | $0.00 | $0.00 | $1,079.77 | $4,345.37 | 0.00 | 0.00 | 7.98 |
| 10 | Cancerous | 0.000% | $0.00 | $0.00 | $1,079.77 | $4,345.37 | 0.00 | 0.00 | 7.98 |
| 10 | Early | 0.000% | $0.00 | $0.00 | $1,079.77 | $4,345.37 | 0.00 | 0.00 | 7.98 |
| 10 | Advanced | 0.000% | $0.00 | $0.00 | $1,079.77 | $4,345.37 | 0.00 | 0.00 | 7.98 |
| 10 | Noncancerous | 0.000% | $0.00 | $0.00 | $1,079.77 | $4,345.37 | 0.00 | 0.00 | 7.98 |
| 10 | No adenoma | 0.000% | $0.00 | $0.00 | $1,079.77 | $4,345.37 | 0.00 | 0.00 | 7.98 |
| 11 | comply | 19.596% | $0.00 | $0.00 | $481.93 | $4,827.30 | 0.65 | 0.13 | 8.55 |
| 11 | Non-comply post colonoscopy | 12.917% | $0.00 | $0.00 | $481.93 | $4,827.30 | 0.00 | 0.00 | 8.55 |
| 11 | Early CRC | 2.818% | $3,196.71 | $90.09 | $481.93 | $4,827.30 | 0.53 | 0.02 | 8.55 |
| 11 | Advanced CRC | 0.068% | $4,305.63 | $2.91 | $481.93 | $4,827.30 | 0.38 | 0.00 | 8.55 |
| 11 | Die other causes | 36.636% | $0.00 | $0.00 | $481.93 | $4,827.30 | 0.66 | 0.24 | 8.55 |
| 11 | Colonoscopy with PureVu due added to inadequate prep or not comply | 14.229% | $541.82 | $77.09 | $481.93 | $4,827.30 | 0.66 | 0.09 | 8.55 |
| 11 | Adenoma surveillance | 13.307% | $2,204.26 | $293.33 | $481.93 | $4,827.30 | 0.66 | 0.09 | 8.55 |
| 11 | Non-compliance with system | 0.000% | $0.00 | $0.00 | $481.93 | $4,827.30 | 0.00 | 0.00 | 8.55 |
| 11 | Dead | 0.430% | $4,305.63 | $18.50 | $481.93 | $4,827.30 | 0.00 | 0.00 | 8.55 |
| 11 | Screening every 10 years - average risk | 0.000% | $0.00 | $0.00 | $481.93 | $4,827.30 | 0.00 | 0.00 | 8.55 |
| 11 | Adequate Prep | 0.000% | $0.00 | $0.00 | $481.93 | $4,827.30 | 0.00 | 0.00 | 8.55 |
| 11 | no adenoma screening | 0.000% | $0.00 | $0.00 | $481.93 | $4,827.30 | 0.00 | 0.00 | 8.55 |
| 11 | Colonoscopy TN | 0.000% | $0.00 | $0.00 | $481.93 | $4,827.30 | 0.00 | 0.00 | 8.55 |
| 11 | Comply | 0.000% | $0.00 | $0.00 | $481.93 | $4,827.30 | 0.00 | 0.00 | 8.55 |
| 11 | Non-comply | 0.000% | $0.00 | $0.00 | $481.93 | $4,827.30 | 0.00 | 0.00 | 8.55 |
| 11 | Colonoscopy FP | 0.000% | $0.00 | $0.00 | $481.93 | $4,827.30 | 0.00 | 0.00 | 8.55 |
| 11 | adenoma | 0.000% | $0.00 | $0.00 | $481.93 | $4,827.30 | 0.00 | 0.00 | 8.55 |
| 11 | Distal Colon (includes descending, sigmoid colon, splenic flexure, rectum) | 0.000% | $0.00 | $0.00 | $481.93 | $4,827.30 | 0.00 | 0.00 | 8.55 |
| 11 | Colonoscopy TP | 0.000% | $0.00 | $0.00 | $481.93 | $4,827.30 | 0.00 | 0.00 | 8.55 |
| 11 | Colonoscopy FN | 0.000% | $0.00 | $0.00 | $481.93 | $4,827.30 | 0.00 | 0.00 | 8.55 |
| 11 | cancer | 0.000% | $0.00 | $0.00 | $481.93 | $4,827.30 | 0.00 | 0.00 | 8.55 |
| 11 | early | 0.000% | $0.00 | $0.00 | $481.93 | $4,827.30 | 0.00 | 0.00 | 8.55 |
| 11 | late | 0.000% | $0.00 | $0.00 | $481.93 | $4,827.30 | 0.00 | 0.00 | 8.55 |
| 11 | no cancer | 0.000% | $0.00 | $0.00 | $481.93 | $4,827.30 | 0.00 | 0.00 | 8.55 |
| 11 | Comply | 0.000% | $0.00 | $0.00 | $481.93 | $4,827.30 | 0.00 | 0.00 | 8.55 |
| 11 | Non-comply | 0.000% | $0.00 | $0.00 | $481.93 | $4,827.30 | 0.00 | 0.00 | 8.55 |
| 11 | Proximal colon (includes ascending, transverse) | 0.000% | $0.00 | $0.00 | $481.93 | $4,827.30 | 0.00 | 0.00 | 8.55 |
| 11 | Colonoscopy TP | 0.000% | $0.00 | $0.00 | $481.93 | $4,827.30 | 0.00 | 0.00 | 8.55 |
| 11 | Colonoscopy FN | 0.000% | $0.00 | $0.00 | $481.93 | $4,827.30 | 0.00 | 0.00 | 8.55 |
| 11 | cancer | 0.000% | $0.00 | $0.00 | $481.93 | $4,827.30 | 0.00 | 0.00 | 8.55 |
| 11 | early | 0.000% | $0.00 | $0.00 | $481.93 | $4,827.30 | 0.00 | 0.00 | 8.55 |
| 11 | late | 0.000% | $0.00 | $0.00 | $481.93 | $4,827.30 | 0.00 | 0.00 | 8.55 |
| 11 | no cancer | 0.000% | $0.00 | $0.00 | $481.93 | $4,827.30 | 0.00 | 0.00 | 8.55 |
| 11 | Comply | 0.000% | $0.00 | $0.00 | $481.93 | $4,827.30 | 0.00 | 0.00 | 8.55 |
| 11 | Non-comply | 0.000% | $0.00 | $0.00 | $481.93 | $4,827.30 | 0.00 | 0.00 | 8.55 |
| 11 | Inadequate Prep | 0.000% | $0.00 | $0.00 | $481.93 | $4,827.30 | 0.00 | 0.00 | 8.55 |
| 11 | PureVu usage | 0.000% | $0.00 | $0.00 | $481.93 | $4,827.30 | 0.00 | 0.00 | 8.55 |
| 11 | Colonoscopy FN | 0.000% | $0.00 | $0.00 | $481.93 | $4,827.30 | 0.00 | 0.00 | 8.55 |
| 11 | cancer | 0.000% | $0.00 | $0.00 | $481.93 | $4,827.30 | 0.00 | 0.00 | 8.55 |
| 11 | early | 0.000% | $0.00 | $0.00 | $481.93 | $4,827.30 | 0.00 | 0.00 | 8.55 |
| 11 | late | 0.000% | $0.00 | $0.00 | $481.93 | $4,827.30 | 0.00 | 0.00 | 8.55 |
| 11 | no cancer | 0.000% | $0.00 | $0.00 | $481.93 | $4,827.30 | 0.00 | 0.00 | 8.55 |
| 11 | Comply | 0.000% | $0.00 | $0.00 | $481.93 | $4,827.30 | 0.00 | 0.00 | 8.55 |
| 11 | Non-comply | 0.000% | $0.00 | $0.00 | $481.93 | $4,827.30 | 0.00 | 0.00 | 8.55 |
| 11 | No screening | 19.596% | $0.00 | $0.00 | $481.93 | $4,827.30 | 0.00 | 0.00 | 8.55 |
| 11 | Adenoma | 5.597% | $0.00 | $0.00 | $481.93 | $4,827.30 | 0.00 | 0.00 | 8.55 |
| 11 | Cancerous | 0.024% | $0.00 | $0.00 | $481.93 | $4,827.30 | 0.00 | 0.00 | 8.55 |
| 11 | Early | 0.020% | $0.00 | $0.00 | $481.93 | $4,827.30 | 0.00 | 0.00 | 8.55 |
| 11 | Advanced | 0.004% | $0.00 | $0.00 | $481.93 | $4,827.30 | 0.00 | 0.00 | 8.55 |
| 11 | Noncancerous | 5.573% | $0.00 | $0.00 | $481.93 | $4,827.30 | 0.00 | 0.00 | 8.55 |
| 11 | comply | 3.957% | $0.00 | $0.00 | $481.93 | $4,827.30 | 0.00 | 0.00 | 8.55 |
| 11 | Not comply | 1.616% | $0.00 | $0.00 | $481.93 | $4,827.30 | 0.00 | 0.00 | 8.55 |
| 11 | No adenoma | 7.319% | $0.00 | $0.00 | $481.93 | $4,827.30 | 0.00 | 0.00 | 8.55 |
| 11 | comply | 4.414% | $0.00 | $0.00 | $481.93 | $4,827.30 | 0.00 | 0.00 | 8.55 |
| 11 | Not comply | 2.906% | $0.00 | $0.00 | $481.93 | $4,827.30 | 0.00 | 0.00 | 8.55 |
| 11 | Continue | 0.054% | $0.00 | $0.00 | $481.93 | $4,827.30 | 0.00 | 0.00 | 8.55 |
| 11 | Die | 0.014% | $0.00 | $0.00 | $481.93 | $4,827.30 | 0.00 | 0.00 | 8.55 |
| 11 | PureVu usage | 11.098% | $0.00 | $0.00 | $481.93 | $4,827.30 | 0.00 | 0.00 | 8.55 |
| 11 | Later followup | 3.130% | $0.00 | $0.00 | $481.93 | $4,827.30 | 0.00 | 0.00 | 8.55 |
| 11 | Redo 2 years | 0.000% | $0.00 | $0.00 | $481.93 | $4,827.30 | 0.00 | 0.00 | 8.55 |
| 11 | comply | 0.000% | $0.00 | $0.00 | $481.93 | $4,827.30 | 0.00 | 0.00 | 8.55 |
| 11 | Not comply | 0.000% | $0.00 | $0.00 | $481.93 | $4,827.30 | 0.00 | 0.00 | 8.55 |
| 11 | No redo | 3.130% | $0.00 | $0.00 | $481.93 | $4,827.30 | 0.00 | 0.00 | 8.55 |
| 11 | Redo in at least 3 years | 0.000% | $0.00 | $0.00 | $481.93 | $4,827.30 | 0.00 | 0.00 | 8.55 |
| 11 | comply | 0.000% | $0.00 | $0.00 | $481.93 | $4,827.30 | 0.00 | 0.00 | 8.55 |
| 11 | Not comply | 0.000% | $0.00 | $0.00 | $481.93 | $4,827.30 | 0.00 | 0.00 | 8.55 |
| 11 | No redo | 13.307% | $0.00 | $0.00 | $481.93 | $4,827.30 | 0.00 | 0.00 | 8.55 |
| 11 | Adenoma | 0.000% | $0.00 | $0.00 | $481.93 | $4,827.30 | 0.00 | 0.00 | 8.55 |
| 11 | Cancerous | 0.000% | $0.00 | $0.00 | $481.93 | $4,827.30 | 0.00 | 0.00 | 8.55 |
| 11 | Early | 0.000% | $0.00 | $0.00 | $481.93 | $4,827.30 | 0.00 | 0.00 | 8.55 |
| 11 | Advanced | 0.000% | $0.00 | $0.00 | $481.93 | $4,827.30 | 0.00 | 0.00 | 8.55 |
| 11 | Noncancerous | 0.000% | $0.00 | $0.00 | $481.93 | $4,827.30 | 0.00 | 0.00 | 8.55 |
| 11 | No adenoma | 0.000% | $0.00 | $0.00 | $481.93 | $4,827.30 | 0.00 | 0.00 | 8.55 |
| 12 | comply | 44.404% | $0.00 | $0.00 | $167.42 | $4,994.72 | 0.63 | 0.28 | 9.15 |
| 12 | Non-comply post colonoscopy | 4.522% | $0.00 | $0.00 | $167.42 | $4,994.72 | 0.00 | 0.00 | 9.15 |
| 12 | Early CRC | 2.839% | $3,103.61 | $88.10 | $167.42 | $4,994.72 | 0.52 | 0.01 | 9.15 |
| 12 | Advanced CRC | 0.058% | $4,180.22 | $2.41 | $167.42 | $4,994.72 | 0.37 | 0.00 | 9.15 |
| 12 | Die other causes | 36.636% | $0.00 | $0.00 | $167.42 | $4,994.72 | 0.64 | 0.23 | 9.15 |
| 12 | Colonoscopy with PureVu due added to inadequate prep or not comply | 11.098% | $526.03 | $58.38 | $167.42 | $4,994.72 | 0.64 | 0.07 | 9.15 |
| 12 | Adenoma surveillance | 0.000% | $2,140.05 | $0.00 | $167.42 | $4,994.72 | 0.64 | 0.00 | 9.15 |
| 12 | Non-compliance with system | 0.000% | $0.00 | $0.00 | $167.42 | $4,994.72 | 0.00 | 0.00 | 9.15 |
| 12 | Dead | 0.443% | $4,180.22 | $18.53 | $167.42 | $4,994.72 | 0.00 | 0.00 | 9.15 |
| 12 | Screening every 10 years - average risk | 0.000% | $0.00 | $0.00 | $167.42 | $4,994.72 | 0.00 | 0.00 | 9.15 |
| 12 | Adequate Prep | 0.000% | $0.00 | $0.00 | $167.42 | $4,994.72 | 0.00 | 0.00 | 9.15 |
| 12 | no adenoma screening | 0.000% | $0.00 | $0.00 | $167.42 | $4,994.72 | 0.00 | 0.00 | 9.15 |
| 12 | Colonoscopy TN | 0.000% | $0.00 | $0.00 | $167.42 | $4,994.72 | 0.00 | 0.00 | 9.15 |
| 12 | Comply | 0.000% | $0.00 | $0.00 | $167.42 | $4,994.72 | 0.00 | 0.00 | 9.15 |
| 12 | Non-comply | 0.000% | $0.00 | $0.00 | $167.42 | $4,994.72 | 0.00 | 0.00 | 9.15 |
| 12 | Colonoscopy FP | 0.000% | $0.00 | $0.00 | $167.42 | $4,994.72 | 0.00 | 0.00 | 9.15 |
| 12 | adenoma | 0.000% | $0.00 | $0.00 | $167.42 | $4,994.72 | 0.00 | 0.00 | 9.15 |
| 12 | Distal Colon (includes descending, sigmoid colon, splenic flexure, rectum) | 0.000% | $0.00 | $0.00 | $167.42 | $4,994.72 | 0.00 | 0.00 | 9.15 |
| 12 | Colonoscopy TP | 0.000% | $0.00 | $0.00 | $167.42 | $4,994.72 | 0.00 | 0.00 | 9.15 |
| 12 | Colonoscopy FN | 0.000% | $0.00 | $0.00 | $167.42 | $4,994.72 | 0.00 | 0.00 | 9.15 |
| 12 | cancer | 0.000% | $0.00 | $0.00 | $167.42 | $4,994.72 | 0.00 | 0.00 | 9.15 |
| 12 | early | 0.000% | $0.00 | $0.00 | $167.42 | $4,994.72 | 0.00 | 0.00 | 9.15 |
| 12 | late | 0.000% | $0.00 | $0.00 | $167.42 | $4,994.72 | 0.00 | 0.00 | 9.15 |
| 12 | no cancer | 0.000% | $0.00 | $0.00 | $167.42 | $4,994.72 | 0.00 | 0.00 | 9.15 |
| 12 | Comply | 0.000% | $0.00 | $0.00 | $167.42 | $4,994.72 | 0.00 | 0.00 | 9.15 |
| 12 | Non-comply | 0.000% | $0.00 | $0.00 | $167.42 | $4,994.72 | 0.00 | 0.00 | 9.15 |
| 12 | Proximal colon (includes ascending, transverse) | 0.000% | $0.00 | $0.00 | $167.42 | $4,994.72 | 0.00 | 0.00 | 9.15 |
| 12 | Colonoscopy TP | 0.000% | $0.00 | $0.00 | $167.42 | $4,994.72 | 0.00 | 0.00 | 9.15 |
| 12 | Colonoscopy FN | 0.000% | $0.00 | $0.00 | $167.42 | $4,994.72 | 0.00 | 0.00 | 9.15 |
| 12 | cancer | 0.000% | $0.00 | $0.00 | $167.42 | $4,994.72 | 0.00 | 0.00 | 9.15 |
| 12 | early | 0.000% | $0.00 | $0.00 | $167.42 | $4,994.72 | 0.00 | 0.00 | 9.15 |
| 12 | late | 0.000% | $0.00 | $0.00 | $167.42 | $4,994.72 | 0.00 | 0.00 | 9.15 |
| 12 | no cancer | 0.000% | $0.00 | $0.00 | $167.42 | $4,994.72 | 0.00 | 0.00 | 9.15 |
| 12 | Comply | 0.000% | $0.00 | $0.00 | $167.42 | $4,994.72 | 0.00 | 0.00 | 9.15 |
| 12 | Non-comply | 0.000% | $0.00 | $0.00 | $167.42 | $4,994.72 | 0.00 | 0.00 | 9.15 |
| 12 | Inadequate Prep | 0.000% | $0.00 | $0.00 | $167.42 | $4,994.72 | 0.00 | 0.00 | 9.15 |
| 12 | PureVu usage | 0.000% | $0.00 | $0.00 | $167.42 | $4,994.72 | 0.00 | 0.00 | 9.15 |
| 12 | Colonoscopy FN | 0.000% | $0.00 | $0.00 | $167.42 | $4,994.72 | 0.00 | 0.00 | 9.15 |
| 12 | cancer | 0.000% | $0.00 | $0.00 | $167.42 | $4,994.72 | 0.00 | 0.00 | 9.15 |
| 12 | early | 0.000% | $0.00 | $0.00 | $167.42 | $4,994.72 | 0.00 | 0.00 | 9.15 |
| 12 | late | 0.000% | $0.00 | $0.00 | $167.42 | $4,994.72 | 0.00 | 0.00 | 9.15 |
| 12 | no cancer | 0.000% | $0.00 | $0.00 | $167.42 | $4,994.72 | 0.00 | 0.00 | 9.15 |
| 12 | Comply | 0.000% | $0.00 | $0.00 | $167.42 | $4,994.72 | 0.00 | 0.00 | 9.15 |
| 12 | Non-comply | 0.000% | $0.00 | $0.00 | $167.42 | $4,994.72 | 0.00 | 0.00 | 9.15 |
| 12 | No screening | 44.404% | $0.00 | $0.00 | $167.42 | $4,994.72 | 0.00 | 0.00 | 9.15 |
| 12 | Adenoma | 1.960% | $0.00 | $0.00 | $167.42 | $4,994.72 | 0.00 | 0.00 | 9.15 |
| 12 | Cancerous | 0.008% | $0.00 | $0.00 | $167.42 | $4,994.72 | 0.00 | 0.00 | 9.15 |
| 12 | Early | 0.007% | $0.00 | $0.00 | $167.42 | $4,994.72 | 0.00 | 0.00 | 9.15 |
| 12 | Advanced | 0.001% | $0.00 | $0.00 | $167.42 | $4,994.72 | 0.00 | 0.00 | 9.15 |
| 12 | Noncancerous | 1.951% | $0.00 | $0.00 | $167.42 | $4,994.72 | 0.00 | 0.00 | 9.15 |
| 12 | comply | 1.385% | $0.00 | $0.00 | $167.42 | $4,994.72 | 0.00 | 0.00 | 9.15 |
| 12 | Not comply | 0.566% | $0.00 | $0.00 | $167.42 | $4,994.72 | 0.00 | 0.00 | 9.15 |
| 12 | No adenoma | 2.563% | $0.00 | $0.00 | $167.42 | $4,994.72 | 0.00 | 0.00 | 9.15 |
| 12 | comply | 1.545% | $0.00 | $0.00 | $167.42 | $4,994.72 | 0.00 | 0.00 | 9.15 |
| 12 | Not comply | 1.017% | $0.00 | $0.00 | $167.42 | $4,994.72 | 0.00 | 0.00 | 9.15 |
| 12 | Continue | 0.046% | $0.00 | $0.00 | $167.42 | $4,994.72 | 0.00 | 0.00 | 9.15 |
| 12 | Die | 0.012% | $0.00 | $0.00 | $167.42 | $4,994.72 | 0.00 | 0.00 | 9.15 |
| 12 | PureVu usage | 8.657% | $0.00 | $0.00 | $167.42 | $4,994.72 | 0.00 | 0.00 | 9.15 |
| 12 | Later followup | 2.442% | $0.00 | $0.00 | $167.42 | $4,994.72 | 0.00 | 0.00 | 9.15 |
| 12 | Redo 2 years | 2.442% | $0.00 | $0.00 | $167.42 | $4,994.72 | 0.00 | 0.00 | 9.15 |
| 12 | comply | 1.365% | $0.00 | $0.00 | $167.42 | $4,994.72 | 0.00 | 0.00 | 9.15 |
| 12 | Not comply | 1.077% | $0.00 | $0.00 | $167.42 | $4,994.72 | 0.00 | 0.00 | 9.15 |
| 12 | No redo | 0.000% | $0.00 | $0.00 | $167.42 | $4,994.72 | 0.00 | 0.00 | 9.15 |
| 12 | Redo in at least 3 years | 0.000% | $0.00 | $0.00 | $167.42 | $4,994.72 | 0.00 | 0.00 | 9.15 |
| 12 | comply | 0.000% | $0.00 | $0.00 | $167.42 | $4,994.72 | 0.00 | 0.00 | 9.15 |
| 12 | Not comply | 0.000% | $0.00 | $0.00 | $167.42 | $4,994.72 | 0.00 | 0.00 | 9.15 |
| 12 | No redo | 0.000% | $0.00 | $0.00 | $167.42 | $4,994.72 | 0.00 | 0.00 | 9.15 |
| 12 | Adenoma | 0.000% | $0.00 | $0.00 | $167.42 | $4,994.72 | 0.00 | 0.00 | 9.15 |
| 12 | Cancerous | 0.000% | $0.00 | $0.00 | $167.42 | $4,994.72 | 0.00 | 0.00 | 9.15 |
| 12 | Early | 0.000% | $0.00 | $0.00 | $167.42 | $4,994.72 | 0.00 | 0.00 | 9.15 |
| 12 | Advanced | 0.000% | $0.00 | $0.00 | $167.42 | $4,994.72 | 0.00 | 0.00 | 9.15 |
| 12 | Noncancerous | 0.000% | $0.00 | $0.00 | $167.42 | $4,994.72 | 0.00 | 0.00 | 9.15 |
| 12 | No adenoma | 0.000% | $0.00 | $0.00 | $167.42 | $4,994.72 | 0.00 | 0.00 | 9.15 |
| 13 | comply | 48.699% | $0.00 | $0.00 | $150.33 | $5,145.05 | 0.61 | 0.30 | 9.74 |
| 13 | Non-comply post colonoscopy | 2.660% | $0.00 | $0.00 | $150.33 | $5,145.05 | 0.00 | 0.00 | 9.74 |
| 13 | Early CRC | 2.846% | $3,013.21 | $85.74 | $150.33 | $5,145.05 | 0.50 | 0.01 | 9.74 |
| 13 | Advanced CRC | 0.047% | $4,058.47 | $1.92 | $150.33 | $5,145.05 | 0.36 | 0.00 | 9.74 |
| 13 | Die other causes | 36.636% | $0.00 | $0.00 | $150.33 | $5,145.05 | 0.62 | 0.23 | 9.74 |
| 13 | Colonoscopy with PureVu due added to inadequate prep or not comply | 8.657% | $510.71 | $44.21 | $150.33 | $5,145.05 | 0.62 | 0.05 | 9.74 |
| 13 | Adenoma surveillance | 0.000% | $2,077.72 | $0.00 | $150.33 | $5,145.05 | 0.62 | 0.00 | 9.74 |
| 13 | Non-compliance with system | 0.000% | $0.00 | $0.00 | $150.33 | $5,145.05 | 0.00 | 0.00 | 9.74 |
| 13 | Dead | 0.455% | $4,058.47 | $18.46 | $150.33 | $5,145.05 | 0.00 | 0.00 | 9.74 |
| 13 | Screening every 10 years - average risk | 0.000% | $0.00 | $0.00 | $150.33 | $5,145.05 | 0.00 | 0.00 | 9.74 |
| 13 | Adequate Prep | 0.000% | $0.00 | $0.00 | $150.33 | $5,145.05 | 0.00 | 0.00 | 9.74 |
| 13 | no adenoma screening | 0.000% | $0.00 | $0.00 | $150.33 | $5,145.05 | 0.00 | 0.00 | 9.74 |
| 13 | Colonoscopy TN | 0.000% | $0.00 | $0.00 | $150.33 | $5,145.05 | 0.00 | 0.00 | 9.74 |
| 13 | Comply | 0.000% | $0.00 | $0.00 | $150.33 | $5,145.05 | 0.00 | 0.00 | 9.74 |
| 13 | Non-comply | 0.000% | $0.00 | $0.00 | $150.33 | $5,145.05 | 0.00 | 0.00 | 9.74 |
| 13 | Colonoscopy FP | 0.000% | $0.00 | $0.00 | $150.33 | $5,145.05 | 0.00 | 0.00 | 9.74 |
| 13 | adenoma | 0.000% | $0.00 | $0.00 | $150.33 | $5,145.05 | 0.00 | 0.00 | 9.74 |
| 13 | Distal Colon (includes descending, sigmoid colon, splenic flexure, rectum) | 0.000% | $0.00 | $0.00 | $150.33 | $5,145.05 | 0.00 | 0.00 | 9.74 |
| 13 | Colonoscopy TP | 0.000% | $0.00 | $0.00 | $150.33 | $5,145.05 | 0.00 | 0.00 | 9.74 |
| 13 | Colonoscopy FN | 0.000% | $0.00 | $0.00 | $150.33 | $5,145.05 | 0.00 | 0.00 | 9.74 |
| 13 | cancer | 0.000% | $0.00 | $0.00 | $150.33 | $5,145.05 | 0.00 | 0.00 | 9.74 |
| 13 | early | 0.000% | $0.00 | $0.00 | $150.33 | $5,145.05 | 0.00 | 0.00 | 9.74 |
| 13 | late | 0.000% | $0.00 | $0.00 | $150.33 | $5,145.05 | 0.00 | 0.00 | 9.74 |
| 13 | no cancer | 0.000% | $0.00 | $0.00 | $150.33 | $5,145.05 | 0.00 | 0.00 | 9.74 |
| 13 | Comply | 0.000% | $0.00 | $0.00 | $150.33 | $5,145.05 | 0.00 | 0.00 | 9.74 |
| 13 | Non-comply | 0.000% | $0.00 | $0.00 | $150.33 | $5,145.05 | 0.00 | 0.00 | 9.74 |
| 13 | Proximal colon (includes ascending, transverse) | 0.000% | $0.00 | $0.00 | $150.33 | $5,145.05 | 0.00 | 0.00 | 9.74 |
| 13 | Colonoscopy TP | 0.000% | $0.00 | $0.00 | $150.33 | $5,145.05 | 0.00 | 0.00 | 9.74 |
| 13 | Colonoscopy FN | 0.000% | $0.00 | $0.00 | $150.33 | $5,145.05 | 0.00 | 0.00 | 9.74 |
| 13 | cancer | 0.000% | $0.00 | $0.00 | $150.33 | $5,145.05 | 0.00 | 0.00 | 9.74 |
| 13 | early | 0.000% | $0.00 | $0.00 | $150.33 | $5,145.05 | 0.00 | 0.00 | 9.74 |
| 13 | late | 0.000% | $0.00 | $0.00 | $150.33 | $5,145.05 | 0.00 | 0.00 | 9.74 |
| 13 | no cancer | 0.000% | $0.00 | $0.00 | $150.33 | $5,145.05 | 0.00 | 0.00 | 9.74 |
| 13 | Comply | 0.000% | $0.00 | $0.00 | $150.33 | $5,145.05 | 0.00 | 0.00 | 9.74 |
| 13 | Non-comply | 0.000% | $0.00 | $0.00 | $150.33 | $5,145.05 | 0.00 | 0.00 | 9.74 |
| 13 | Inadequate Prep | 0.000% | $0.00 | $0.00 | $150.33 | $5,145.05 | 0.00 | 0.00 | 9.74 |
| 13 | PureVu usage | 0.000% | $0.00 | $0.00 | $150.33 | $5,145.05 | 0.00 | 0.00 | 9.74 |
| 13 | Colonoscopy FN | 0.000% | $0.00 | $0.00 | $150.33 | $5,145.05 | 0.00 | 0.00 | 9.74 |
| 13 | cancer | 0.000% | $0.00 | $0.00 | $150.33 | $5,145.05 | 0.00 | 0.00 | 9.74 |
| 13 | early | 0.000% | $0.00 | $0.00 | $150.33 | $5,145.05 | 0.00 | 0.00 | 9.74 |
| 13 | late | 0.000% | $0.00 | $0.00 | $150.33 | $5,145.05 | 0.00 | 0.00 | 9.74 |
| 13 | no cancer | 0.000% | $0.00 | $0.00 | $150.33 | $5,145.05 | 0.00 | 0.00 | 9.74 |
| 13 | Comply | 0.000% | $0.00 | $0.00 | $150.33 | $5,145.05 | 0.00 | 0.00 | 9.74 |
| 13 | Non-comply | 0.000% | $0.00 | $0.00 | $150.33 | $5,145.05 | 0.00 | 0.00 | 9.74 |
| 13 | No screening | 48.699% | $0.00 | $0.00 | $150.33 | $5,145.05 | 0.00 | 0.00 | 9.74 |
| 13 | Adenoma | 1.153% | $0.00 | $0.00 | $150.33 | $5,145.05 | 0.00 | 0.00 | 9.74 |
| 13 | Cancerous | 0.005% | $0.00 | $0.00 | $150.33 | $5,145.05 | 0.00 | 0.00 | 9.74 |
| 13 | Early | 0.004% | $0.00 | $0.00 | $150.33 | $5,145.05 | 0.00 | 0.00 | 9.74 |
| 13 | Advanced | 0.001% | $0.00 | $0.00 | $150.33 | $5,145.05 | 0.00 | 0.00 | 9.74 |
| 13 | Noncancerous | 1.148% | $0.00 | $0.00 | $150.33 | $5,145.05 | 0.00 | 0.00 | 9.74 |
| 13 | comply | 0.815% | $0.00 | $0.00 | $150.33 | $5,145.05 | 0.00 | 0.00 | 9.74 |
| 13 | Not comply | 0.333% | $0.00 | $0.00 | $150.33 | $5,145.05 | 0.00 | 0.00 | 9.74 |
| 13 | No adenoma | 1.507% | $0.00 | $0.00 | $150.33 | $5,145.05 | 0.00 | 0.00 | 9.74 |
| 13 | comply | 0.909% | $0.00 | $0.00 | $150.33 | $5,145.05 | 0.00 | 0.00 | 9.74 |
| 13 | Not comply | 0.598% | $0.00 | $0.00 | $150.33 | $5,145.05 | 0.00 | 0.00 | 9.74 |
| 13 | Continue | 0.038% | $0.00 | $0.00 | $150.33 | $5,145.05 | 0.00 | 0.00 | 9.74 |
| 13 | Die | 0.009% | $0.00 | $0.00 | $150.33 | $5,145.05 | 0.00 | 0.00 | 9.74 |
| 13 | PureVu usage | 6.752% | $0.00 | $0.00 | $150.33 | $5,145.05 | 0.00 | 0.00 | 9.74 |
| 13 | Later followup | 1.904% | $0.00 | $0.00 | $150.33 | $5,145.05 | 0.00 | 0.00 | 9.74 |
| 13 | Redo 2 years | 0.000% | $0.00 | $0.00 | $150.33 | $5,145.05 | 0.00 | 0.00 | 9.74 |
| 13 | comply | 0.000% | $0.00 | $0.00 | $150.33 | $5,145.05 | 0.00 | 0.00 | 9.74 |
| 13 | Not comply | 0.000% | $0.00 | $0.00 | $150.33 | $5,145.05 | 0.00 | 0.00 | 9.74 |
| 13 | No redo | 1.904% | $0.00 | $0.00 | $150.33 | $5,145.05 | 0.00 | 0.00 | 9.74 |
| 13 | Redo in at least 3 years | 0.000% | $0.00 | $0.00 | $150.33 | $5,145.05 | 0.00 | 0.00 | 9.74 |
| 13 | comply | 0.000% | $0.00 | $0.00 | $150.33 | $5,145.05 | 0.00 | 0.00 | 9.74 |
| 13 | Not comply | 0.000% | $0.00 | $0.00 | $150.33 | $5,145.05 | 0.00 | 0.00 | 9.74 |
| 13 | No redo | 0.000% | $0.00 | $0.00 | $150.33 | $5,145.05 | 0.00 | 0.00 | 9.74 |
| 13 | Adenoma | 0.000% | $0.00 | $0.00 | $150.33 | $5,145.05 | 0.00 | 0.00 | 9.74 |
| 13 | Cancerous | 0.000% | $0.00 | $0.00 | $150.33 | $5,145.05 | 0.00 | 0.00 | 9.74 |
| 13 | Early | 0.000% | $0.00 | $0.00 | $150.33 | $5,145.05 | 0.00 | 0.00 | 9.74 |
| 13 | Advanced | 0.000% | $0.00 | $0.00 | $150.33 | $5,145.05 | 0.00 | 0.00 | 9.74 |
| 13 | Noncancerous | 0.000% | $0.00 | $0.00 | $150.33 | $5,145.05 | 0.00 | 0.00 | 9.74 |
| 13 | No adenoma | 0.000% | $0.00 | $0.00 | $150.33 | $5,145.05 | 0.00 | 0.00 | 9.74 |
| 14 | comply | 52.328% | $0.00 | $0.00 | $136.66 | $5,281.72 | 0.60 | 0.31 | 10.33 |
| 14 | Non-comply post colonoscopy | 0.931% | $0.00 | $0.00 | $136.66 | $5,281.72 | 0.00 | 0.00 | 10.33 |
| 14 | Early CRC | 2.850% | $2,925.45 | $83.37 | $136.66 | $5,281.72 | 0.49 | 0.01 | 10.33 |
| 14 | Advanced CRC | 0.039% | $3,940.26 | $1.52 | $136.66 | $5,281.72 | 0.35 | 0.00 | 10.33 |
| 14 | Die other causes | 36.636% | $0.00 | $0.00 | $136.66 | $5,281.72 | 0.60 | 0.22 | 10.33 |
| 14 | Colonoscopy with PureVu due added to inadequate prep or not comply | 6.752% | $495.84 | $33.48 | $136.66 | $5,281.72 | 0.60 | 0.04 | 10.33 |
| 14 | Adenoma surveillance | 0.000% | $2,017.21 | $0.00 | $136.66 | $5,281.72 | 0.60 | 0.00 | 10.33 |
| 14 | Non-compliance with system | 0.000% | $0.00 | $0.00 | $136.66 | $5,281.72 | 0.00 | 0.00 | 10.33 |
| 14 | Dead | 0.464% | $3,940.26 | $18.29 | $136.66 | $5,281.72 | 0.00 | 0.00 | 10.33 |
| 14 | Screening every 10 years - average risk | 0.000% | $0.00 | $0.00 | $136.66 | $5,281.72 | 0.00 | 0.00 | 10.33 |
| 14 | Adequate Prep | 0.000% | $0.00 | $0.00 | $136.66 | $5,281.72 | 0.00 | 0.00 | 10.33 |
| 14 | no adenoma screening | 0.000% | $0.00 | $0.00 | $136.66 | $5,281.72 | 0.00 | 0.00 | 10.33 |
| 14 | Colonoscopy TN | 0.000% | $0.00 | $0.00 | $136.66 | $5,281.72 | 0.00 | 0.00 | 10.33 |
| 14 | Comply | 0.000% | $0.00 | $0.00 | $136.66 | $5,281.72 | 0.00 | 0.00 | 10.33 |
| 14 | Non-comply | 0.000% | $0.00 | $0.00 | $136.66 | $5,281.72 | 0.00 | 0.00 | 10.33 |
| 14 | Colonoscopy FP | 0.000% | $0.00 | $0.00 | $136.66 | $5,281.72 | 0.00 | 0.00 | 10.33 |
| 14 | adenoma | 0.000% | $0.00 | $0.00 | $136.66 | $5,281.72 | 0.00 | 0.00 | 10.33 |
| 14 | Distal Colon (includes descending, sigmoid colon, splenic flexure, rectum) | 0.000% | $0.00 | $0.00 | $136.66 | $5,281.72 | 0.00 | 0.00 | 10.33 |
| 14 | Colonoscopy TP | 0.000% | $0.00 | $0.00 | $136.66 | $5,281.72 | 0.00 | 0.00 | 10.33 |
| 14 | Colonoscopy FN | 0.000% | $0.00 | $0.00 | $136.66 | $5,281.72 | 0.00 | 0.00 | 10.33 |
| 14 | cancer | 0.000% | $0.00 | $0.00 | $136.66 | $5,281.72 | 0.00 | 0.00 | 10.33 |
| 14 | early | 0.000% | $0.00 | $0.00 | $136.66 | $5,281.72 | 0.00 | 0.00 | 10.33 |
| 14 | late | 0.000% | $0.00 | $0.00 | $136.66 | $5,281.72 | 0.00 | 0.00 | 10.33 |
| 14 | no cancer | 0.000% | $0.00 | $0.00 | $136.66 | $5,281.72 | 0.00 | 0.00 | 10.33 |
| 14 | Comply | 0.000% | $0.00 | $0.00 | $136.66 | $5,281.72 | 0.00 | 0.00 | 10.33 |
| 14 | Non-comply | 0.000% | $0.00 | $0.00 | $136.66 | $5,281.72 | 0.00 | 0.00 | 10.33 |
| 14 | Proximal colon (includes ascending, transverse) | 0.000% | $0.00 | $0.00 | $136.66 | $5,281.72 | 0.00 | 0.00 | 10.33 |
| 14 | Colonoscopy TP | 0.000% | $0.00 | $0.00 | $136.66 | $5,281.72 | 0.00 | 0.00 | 10.33 |
| 14 | Colonoscopy FN | 0.000% | $0.00 | $0.00 | $136.66 | $5,281.72 | 0.00 | 0.00 | 10.33 |
| 14 | cancer | 0.000% | $0.00 | $0.00 | $136.66 | $5,281.72 | 0.00 | 0.00 | 10.33 |
| 14 | early | 0.000% | $0.00 | $0.00 | $136.66 | $5,281.72 | 0.00 | 0.00 | 10.33 |
| 14 | late | 0.000% | $0.00 | $0.00 | $136.66 | $5,281.72 | 0.00 | 0.00 | 10.33 |
| 14 | no cancer | 0.000% | $0.00 | $0.00 | $136.66 | $5,281.72 | 0.00 | 0.00 | 10.33 |
| 14 | Comply | 0.000% | $0.00 | $0.00 | $136.66 | $5,281.72 | 0.00 | 0.00 | 10.33 |
| 14 | Non-comply | 0.000% | $0.00 | $0.00 | $136.66 | $5,281.72 | 0.00 | 0.00 | 10.33 |
| 14 | Inadequate Prep | 0.000% | $0.00 | $0.00 | $136.66 | $5,281.72 | 0.00 | 0.00 | 10.33 |
| 14 | PureVu usage | 0.000% | $0.00 | $0.00 | $136.66 | $5,281.72 | 0.00 | 0.00 | 10.33 |
| 14 | Colonoscopy FN | 0.000% | $0.00 | $0.00 | $136.66 | $5,281.72 | 0.00 | 0.00 | 10.33 |
| 14 | cancer | 0.000% | $0.00 | $0.00 | $136.66 | $5,281.72 | 0.00 | 0.00 | 10.33 |
| 14 | early | 0.000% | $0.00 | $0.00 | $136.66 | $5,281.72 | 0.00 | 0.00 | 10.33 |
| 14 | late | 0.000% | $0.00 | $0.00 | $136.66 | $5,281.72 | 0.00 | 0.00 | 10.33 |
| 14 | no cancer | 0.000% | $0.00 | $0.00 | $136.66 | $5,281.72 | 0.00 | 0.00 | 10.33 |
| 14 | Comply | 0.000% | $0.00 | $0.00 | $136.66 | $5,281.72 | 0.00 | 0.00 | 10.33 |
| 14 | Non-comply | 0.000% | $0.00 | $0.00 | $136.66 | $5,281.72 | 0.00 | 0.00 | 10.33 |
| 14 | No screening | 52.328% | $0.00 | $0.00 | $136.66 | $5,281.72 | 0.00 | 0.00 | 10.33 |
| 14 | Adenoma | 0.404% | $0.00 | $0.00 | $136.66 | $5,281.72 | 0.00 | 0.00 | 10.33 |
| 14 | Cancerous | 0.002% | $0.00 | $0.00 | $136.66 | $5,281.72 | 0.00 | 0.00 | 10.33 |
| 14 | Early | 0.001% | $0.00 | $0.00 | $136.66 | $5,281.72 | 0.00 | 0.00 | 10.33 |
| 14 | Advanced | 0.000% | $0.00 | $0.00 | $136.66 | $5,281.72 | 0.00 | 0.00 | 10.33 |
| 14 | Noncancerous | 0.402% | $0.00 | $0.00 | $136.66 | $5,281.72 | 0.00 | 0.00 | 10.33 |
| 14 | comply | 0.285% | $0.00 | $0.00 | $136.66 | $5,281.72 | 0.00 | 0.00 | 10.33 |
| 14 | Not comply | 0.117% | $0.00 | $0.00 | $136.66 | $5,281.72 | 0.00 | 0.00 | 10.33 |
| 14 | No adenoma | 0.528% | $0.00 | $0.00 | $136.66 | $5,281.72 | 0.00 | 0.00 | 10.33 |
| 14 | comply | 0.318% | $0.00 | $0.00 | $136.66 | $5,281.72 | 0.00 | 0.00 | 10.33 |
| 14 | Not comply | 0.209% | $0.00 | $0.00 | $136.66 | $5,281.72 | 0.00 | 0.00 | 10.33 |
| 14 | Continue | 0.031% | $0.00 | $0.00 | $136.66 | $5,281.72 | 0.00 | 0.00 | 10.33 |
| 14 | Die | 0.008% | $0.00 | $0.00 | $136.66 | $5,281.72 | 0.00 | 0.00 | 10.33 |
| 14 | PureVu usage | 5.267% | $0.00 | $0.00 | $136.66 | $5,281.72 | 0.00 | 0.00 | 10.33 |
| 14 | Later followup | 1.486% | $0.00 | $0.00 | $136.66 | $5,281.72 | 0.00 | 0.00 | 10.33 |
| 14 | Redo 2 years | 1.486% | $0.00 | $0.00 | $136.66 | $5,281.72 | 0.00 | 0.00 | 10.33 |
| 14 | comply | 0.830% | $0.00 | $0.00 | $136.66 | $5,281.72 | 0.00 | 0.00 | 10.33 |
| 14 | Not comply | 0.655% | $0.00 | $0.00 | $136.66 | $5,281.72 | 0.00 | 0.00 | 10.33 |
| 14 | No redo | 0.000% | $0.00 | $0.00 | $136.66 | $5,281.72 | 0.00 | 0.00 | 10.33 |
| 14 | Redo in at least 3 years | 0.000% | $0.00 | $0.00 | $136.66 | $5,281.72 | 0.00 | 0.00 | 10.33 |
| 14 | comply | 0.000% | $0.00 | $0.00 | $136.66 | $5,281.72 | 0.00 | 0.00 | 10.33 |
| 14 | Not comply | 0.000% | $0.00 | $0.00 | $136.66 | $5,281.72 | 0.00 | 0.00 | 10.33 |
| 14 | No redo | 0.000% | $0.00 | $0.00 | $136.66 | $5,281.72 | 0.00 | 0.00 | 10.33 |
| 14 | Adenoma | 0.000% | $0.00 | $0.00 | $136.66 | $5,281.72 | 0.00 | 0.00 | 10.33 |
| 14 | Cancerous | 0.000% | $0.00 | $0.00 | $136.66 | $5,281.72 | 0.00 | 0.00 | 10.33 |
| 14 | Early | 0.000% | $0.00 | $0.00 | $136.66 | $5,281.72 | 0.00 | 0.00 | 10.33 |
| 14 | Advanced | 0.000% | $0.00 | $0.00 | $136.66 | $5,281.72 | 0.00 | 0.00 | 10.33 |
| 14 | Noncancerous | 0.000% | $0.00 | $0.00 | $136.66 | $5,281.72 | 0.00 | 0.00 | 10.33 |
| 14 | No adenoma | 0.000% | $0.00 | $0.00 | $136.66 | $5,281.72 | 0.00 | 0.00 | 10.33 |
| 15 | comply | 53.762% | $0.00 | $0.00 | $125.58 | $5,407.30 | 0.58 | 0.31 | 10.89 |
| 15 | Non-comply post colonoscopy | 0.981% | $0.00 | $0.00 | $125.58 | $5,407.30 | 0.00 | 0.00 | 10.89 |
| 15 | Early CRC | 2.851% | $2,840.24 | $80.98 | $125.58 | $5,407.30 | 0.47 | 0.01 | 10.89 |
| 15 | Advanced CRC | 0.031% | $3,825.50 | $1.19 | $125.58 | $5,407.30 | 0.34 | 0.00 | 10.89 |
| 15 | Die other causes | 36.636% | $0.00 | $0.00 | $125.58 | $5,407.30 | 0.58 | 0.21 | 10.89 |
| 15 | Colonoscopy with PureVu due added to inadequate prep or not comply | 5.267% | $481.40 | $25.35 | $125.58 | $5,407.30 | 0.58 | 0.03 | 10.89 |
| 15 | Adenoma surveillance | 0.000% | $1,958.45 | $0.00 | $125.58 | $5,407.30 | 0.58 | 0.00 | 10.89 |
| 15 | Non-compliance with system | 0.000% | $0.00 | $0.00 | $125.58 | $5,407.30 | 0.00 | 0.00 | 10.89 |
| 15 | Dead | 0.472% | $3,825.50 | $18.05 | $125.58 | $5,407.30 | 0.00 | 0.00 | 10.89 |
| 15 | Screening every 10 years - average risk | 0.000% | $0.00 | $0.00 | $125.58 | $5,407.30 | 0.00 | 0.00 | 10.89 |
| 15 | Adequate Prep | 0.000% | $0.00 | $0.00 | $125.58 | $5,407.30 | 0.00 | 0.00 | 10.89 |
| 15 | no adenoma screening | 0.000% | $0.00 | $0.00 | $125.58 | $5,407.30 | 0.00 | 0.00 | 10.89 |
| 15 | Colonoscopy TN | 0.000% | $0.00 | $0.00 | $125.58 | $5,407.30 | 0.00 | 0.00 | 10.89 |
| 15 | Comply | 0.000% | $0.00 | $0.00 | $125.58 | $5,407.30 | 0.00 | 0.00 | 10.89 |
| 15 | Non-comply | 0.000% | $0.00 | $0.00 | $125.58 | $5,407.30 | 0.00 | 0.00 | 10.89 |
| 15 | Colonoscopy FP | 0.000% | $0.00 | $0.00 | $125.58 | $5,407.30 | 0.00 | 0.00 | 10.89 |
| 15 | adenoma | 0.000% | $0.00 | $0.00 | $125.58 | $5,407.30 | 0.00 | 0.00 | 10.89 |
| 15 | Distal Colon (includes descending, sigmoid colon, splenic flexure, rectum) | 0.000% | $0.00 | $0.00 | $125.58 | $5,407.30 | 0.00 | 0.00 | 10.89 |
| 15 | Colonoscopy TP | 0.000% | $0.00 | $0.00 | $125.58 | $5,407.30 | 0.00 | 0.00 | 10.89 |
| 15 | Colonoscopy FN | 0.000% | $0.00 | $0.00 | $125.58 | $5,407.30 | 0.00 | 0.00 | 10.89 |
| 15 | cancer | 0.000% | $0.00 | $0.00 | $125.58 | $5,407.30 | 0.00 | 0.00 | 10.89 |
| 15 | early | 0.000% | $0.00 | $0.00 | $125.58 | $5,407.30 | 0.00 | 0.00 | 10.89 |
| 15 | late | 0.000% | $0.00 | $0.00 | $125.58 | $5,407.30 | 0.00 | 0.00 | 10.89 |
| 15 | no cancer | 0.000% | $0.00 | $0.00 | $125.58 | $5,407.30 | 0.00 | 0.00 | 10.89 |
| 15 | Comply | 0.000% | $0.00 | $0.00 | $125.58 | $5,407.30 | 0.00 | 0.00 | 10.89 |
| 15 | Non-comply | 0.000% | $0.00 | $0.00 | $125.58 | $5,407.30 | 0.00 | 0.00 | 10.89 |
| 15 | Proximal colon (includes ascending, transverse) | 0.000% | $0.00 | $0.00 | $125.58 | $5,407.30 | 0.00 | 0.00 | 10.89 |
| 15 | Colonoscopy TP | 0.000% | $0.00 | $0.00 | $125.58 | $5,407.30 | 0.00 | 0.00 | 10.89 |
| 15 | Colonoscopy FN | 0.000% | $0.00 | $0.00 | $125.58 | $5,407.30 | 0.00 | 0.00 | 10.89 |
| 15 | cancer | 0.000% | $0.00 | $0.00 | $125.58 | $5,407.30 | 0.00 | 0.00 | 10.89 |
| 15 | early | 0.000% | $0.00 | $0.00 | $125.58 | $5,407.30 | 0.00 | 0.00 | 10.89 |
| 15 | late | 0.000% | $0.00 | $0.00 | $125.58 | $5,407.30 | 0.00 | 0.00 | 10.89 |
| 15 | no cancer | 0.000% | $0.00 | $0.00 | $125.58 | $5,407.30 | 0.00 | 0.00 | 10.89 |
| 15 | Comply | 0.000% | $0.00 | $0.00 | $125.58 | $5,407.30 | 0.00 | 0.00 | 10.89 |
| 15 | Non-comply | 0.000% | $0.00 | $0.00 | $125.58 | $5,407.30 | 0.00 | 0.00 | 10.89 |
| 15 | Inadequate Prep | 0.000% | $0.00 | $0.00 | $125.58 | $5,407.30 | 0.00 | 0.00 | 10.89 |
| 15 | PureVu usage | 0.000% | $0.00 | $0.00 | $125.58 | $5,407.30 | 0.00 | 0.00 | 10.89 |
| 15 | Colonoscopy FN | 0.000% | $0.00 | $0.00 | $125.58 | $5,407.30 | 0.00 | 0.00 | 10.89 |
| 15 | cancer | 0.000% | $0.00 | $0.00 | $125.58 | $5,407.30 | 0.00 | 0.00 | 10.89 |
| 15 | early | 0.000% | $0.00 | $0.00 | $125.58 | $5,407.30 | 0.00 | 0.00 | 10.89 |
| 15 | late | 0.000% | $0.00 | $0.00 | $125.58 | $5,407.30 | 0.00 | 0.00 | 10.89 |
| 15 | no cancer | 0.000% | $0.00 | $0.00 | $125.58 | $5,407.30 | 0.00 | 0.00 | 10.89 |
| 15 | Comply | 0.000% | $0.00 | $0.00 | $125.58 | $5,407.30 | 0.00 | 0.00 | 10.89 |
| 15 | Non-comply | 0.000% | $0.00 | $0.00 | $125.58 | $5,407.30 | 0.00 | 0.00 | 10.89 |
| 15 | No screening | 53.762% | $0.00 | $0.00 | $125.58 | $5,407.30 | 0.00 | 0.00 | 10.89 |
| 15 | Adenoma | 0.425% | $0.00 | $0.00 | $125.58 | $5,407.30 | 0.00 | 0.00 | 10.89 |
| 15 | Cancerous | 0.002% | $0.00 | $0.00 | $125.58 | $5,407.30 | 0.00 | 0.00 | 10.89 |
| 15 | Early | 0.002% | $0.00 | $0.00 | $125.58 | $5,407.30 | 0.00 | 0.00 | 10.89 |
| 15 | Advanced | 0.000% | $0.00 | $0.00 | $125.58 | $5,407.30 | 0.00 | 0.00 | 10.89 |
| 15 | Noncancerous | 0.423% | $0.00 | $0.00 | $125.58 | $5,407.30 | 0.00 | 0.00 | 10.89 |
| 15 | comply | 0.301% | $0.00 | $0.00 | $125.58 | $5,407.30 | 0.00 | 0.00 | 10.89 |
| 15 | Not comply | 0.123% | $0.00 | $0.00 | $125.58 | $5,407.30 | 0.00 | 0.00 | 10.89 |
| 15 | No adenoma | 0.556% | $0.00 | $0.00 | $125.58 | $5,407.30 | 0.00 | 0.00 | 10.89 |
| 15 | comply | 0.335% | $0.00 | $0.00 | $125.58 | $5,407.30 | 0.00 | 0.00 | 10.89 |
| 15 | Not comply | 0.221% | $0.00 | $0.00 | $125.58 | $5,407.30 | 0.00 | 0.00 | 10.89 |
| 15 | Continue | 0.025% | $0.00 | $0.00 | $125.58 | $5,407.30 | 0.00 | 0.00 | 10.89 |
| 15 | Die | 0.006% | $0.00 | $0.00 | $125.58 | $5,407.30 | 0.00 | 0.00 | 10.89 |
| 15 | PureVu usage | 4.108% | $0.00 | $0.00 | $125.58 | $5,407.30 | 0.00 | 0.00 | 10.89 |
| 15 | Later followup | 1.159% | $0.00 | $0.00 | $125.58 | $5,407.30 | 0.00 | 0.00 | 10.89 |
| 15 | Redo 2 years | 0.000% | $0.00 | $0.00 | $125.58 | $5,407.30 | 0.00 | 0.00 | 10.89 |
| 15 | comply | 0.000% | $0.00 | $0.00 | $125.58 | $5,407.30 | 0.00 | 0.00 | 10.89 |
| 15 | Not comply | 0.000% | $0.00 | $0.00 | $125.58 | $5,407.30 | 0.00 | 0.00 | 10.89 |
| 15 | No redo | 1.159% | $0.00 | $0.00 | $125.58 | $5,407.30 | 0.00 | 0.00 | 10.89 |
| 15 | Redo in at least 3 years | 0.000% | $0.00 | $0.00 | $125.58 | $5,407.30 | 0.00 | 0.00 | 10.89 |
| 15 | comply | 0.000% | $0.00 | $0.00 | $125.58 | $5,407.30 | 0.00 | 0.00 | 10.89 |
| 15 | Not comply | 0.000% | $0.00 | $0.00 | $125.58 | $5,407.30 | 0.00 | 0.00 | 10.89 |
| 15 | No redo | 0.000% | $0.00 | $0.00 | $125.58 | $5,407.30 | 0.00 | 0.00 | 10.89 |
| 15 | Adenoma | 0.000% | $0.00 | $0.00 | $125.58 | $5,407.30 | 0.00 | 0.00 | 10.89 |
| 15 | Cancerous | 0.000% | $0.00 | $0.00 | $125.58 | $5,407.30 | 0.00 | 0.00 | 10.89 |
| 15 | Early | 0.000% | $0.00 | $0.00 | $125.58 | $5,407.30 | 0.00 | 0.00 | 10.89 |
| 15 | Advanced | 0.000% | $0.00 | $0.00 | $125.58 | $5,407.30 | 0.00 | 0.00 | 10.89 |
| 15 | Noncancerous | 0.000% | $0.00 | $0.00 | $125.58 | $5,407.30 | 0.00 | 0.00 | 10.89 |
| 15 | No adenoma | 0.000% | $0.00 | $0.00 | $125.58 | $5,407.30 | 0.00 | 0.00 | 10.89 |
| 16 | comply | 55.556% | $0.00 | $0.00 | $116.56 | $5,523.86 | 0.56 | 0.31 | 11.45 |
| 16 | Non-comply post colonoscopy | 0.343% | $0.00 | $0.00 | $116.56 | $5,523.86 | 0.00 | 0.00 | 11.45 |
| 16 | Early CRC | 2.853% | $2,757.51 | $78.66 | $116.56 | $5,523.86 | 0.46 | 0.01 | 11.45 |
| 16 | Advanced CRC | 0.025% | $3,714.07 | $0.94 | $116.56 | $5,523.86 | 0.33 | 0.00 | 11.45 |
| 16 | Die other causes | 36.636% | $0.00 | $0.00 | $116.56 | $5,523.86 | 0.57 | 0.21 | 11.45 |
| 16 | Colonoscopy with PureVu due added to inadequate prep or not comply | 4.108% | $467.38 | $19.20 | $116.56 | $5,523.86 | 0.57 | 0.02 | 11.45 |
| 16 | Adenoma surveillance | 0.000% | $1,901.41 | $0.00 | $116.56 | $5,523.86 | 0.57 | 0.00 | 11.45 |
| 16 | Non-compliance with system | 0.000% | $0.00 | $0.00 | $116.56 | $5,523.86 | 0.00 | 0.00 | 11.45 |
| 16 | Dead | 0.478% | $3,714.07 | $17.76 | $116.56 | $5,523.86 | 0.00 | 0.00 | 11.45 |
| 16 | Screening every 10 years - average risk | 0.000% | $0.00 | $0.00 | $116.56 | $5,523.86 | 0.00 | 0.00 | 11.45 |
| 16 | Adequate Prep | 0.000% | $0.00 | $0.00 | $116.56 | $5,523.86 | 0.00 | 0.00 | 11.45 |
| 16 | no adenoma screening | 0.000% | $0.00 | $0.00 | $116.56 | $5,523.86 | 0.00 | 0.00 | 11.45 |
| 16 | Colonoscopy TN | 0.000% | $0.00 | $0.00 | $116.56 | $5,523.86 | 0.00 | 0.00 | 11.45 |
| 16 | Comply | 0.000% | $0.00 | $0.00 | $116.56 | $5,523.86 | 0.00 | 0.00 | 11.45 |
| 16 | Non-comply | 0.000% | $0.00 | $0.00 | $116.56 | $5,523.86 | 0.00 | 0.00 | 11.45 |
| 16 | Colonoscopy FP | 0.000% | $0.00 | $0.00 | $116.56 | $5,523.86 | 0.00 | 0.00 | 11.45 |
| 16 | adenoma | 0.000% | $0.00 | $0.00 | $116.56 | $5,523.86 | 0.00 | 0.00 | 11.45 |
| 16 | Distal Colon (includes descending, sigmoid colon, splenic flexure, rectum) | 0.000% | $0.00 | $0.00 | $116.56 | $5,523.86 | 0.00 | 0.00 | 11.45 |
| 16 | Colonoscopy TP | 0.000% | $0.00 | $0.00 | $116.56 | $5,523.86 | 0.00 | 0.00 | 11.45 |
| 16 | Colonoscopy FN | 0.000% | $0.00 | $0.00 | $116.56 | $5,523.86 | 0.00 | 0.00 | 11.45 |
| 16 | cancer | 0.000% | $0.00 | $0.00 | $116.56 | $5,523.86 | 0.00 | 0.00 | 11.45 |
| 16 | early | 0.000% | $0.00 | $0.00 | $116.56 | $5,523.86 | 0.00 | 0.00 | 11.45 |
| 16 | late | 0.000% | $0.00 | $0.00 | $116.56 | $5,523.86 | 0.00 | 0.00 | 11.45 |
| 16 | no cancer | 0.000% | $0.00 | $0.00 | $116.56 | $5,523.86 | 0.00 | 0.00 | 11.45 |
| 16 | Comply | 0.000% | $0.00 | $0.00 | $116.56 | $5,523.86 | 0.00 | 0.00 | 11.45 |
| 16 | Non-comply | 0.000% | $0.00 | $0.00 | $116.56 | $5,523.86 | 0.00 | 0.00 | 11.45 |
| 16 | Proximal colon (includes ascending, transverse) | 0.000% | $0.00 | $0.00 | $116.56 | $5,523.86 | 0.00 | 0.00 | 11.45 |
| 16 | Colonoscopy TP | 0.000% | $0.00 | $0.00 | $116.56 | $5,523.86 | 0.00 | 0.00 | 11.45 |
| 16 | Colonoscopy FN | 0.000% | $0.00 | $0.00 | $116.56 | $5,523.86 | 0.00 | 0.00 | 11.45 |
| 16 | cancer | 0.000% | $0.00 | $0.00 | $116.56 | $5,523.86 | 0.00 | 0.00 | 11.45 |
| 16 | early | 0.000% | $0.00 | $0.00 | $116.56 | $5,523.86 | 0.00 | 0.00 | 11.45 |
| 16 | late | 0.000% | $0.00 | $0.00 | $116.56 | $5,523.86 | 0.00 | 0.00 | 11.45 |
| 16 | no cancer | 0.000% | $0.00 | $0.00 | $116.56 | $5,523.86 | 0.00 | 0.00 | 11.45 |
| 16 | Comply | 0.000% | $0.00 | $0.00 | $116.56 | $5,523.86 | 0.00 | 0.00 | 11.45 |
| 16 | Non-comply | 0.000% | $0.00 | $0.00 | $116.56 | $5,523.86 | 0.00 | 0.00 | 11.45 |
| 16 | Inadequate Prep | 0.000% | $0.00 | $0.00 | $116.56 | $5,523.86 | 0.00 | 0.00 | 11.45 |
| 16 | PureVu usage | 0.000% | $0.00 | $0.00 | $116.56 | $5,523.86 | 0.00 | 0.00 | 11.45 |
| 16 | Colonoscopy FN | 0.000% | $0.00 | $0.00 | $116.56 | $5,523.86 | 0.00 | 0.00 | 11.45 |
| 16 | cancer | 0.000% | $0.00 | $0.00 | $116.56 | $5,523.86 | 0.00 | 0.00 | 11.45 |
| 16 | early | 0.000% | $0.00 | $0.00 | $116.56 | $5,523.86 | 0.00 | 0.00 | 11.45 |
| 16 | late | 0.000% | $0.00 | $0.00 | $116.56 | $5,523.86 | 0.00 | 0.00 | 11.45 |
| 16 | no cancer | 0.000% | $0.00 | $0.00 | $116.56 | $5,523.86 | 0.00 | 0.00 | 11.45 |
| 16 | Comply | 0.000% | $0.00 | $0.00 | $116.56 | $5,523.86 | 0.00 | 0.00 | 11.45 |
| 16 | Non-comply | 0.000% | $0.00 | $0.00 | $116.56 | $5,523.86 | 0.00 | 0.00 | 11.45 |
| 16 | No screening | 55.556% | $0.00 | $0.00 | $116.56 | $5,523.86 | 0.00 | 0.00 | 11.45 |
| 16 | Adenoma | 0.149% | $0.00 | $0.00 | $116.56 | $5,523.86 | 0.00 | 0.00 | 11.45 |
| 16 | Cancerous | 0.001% | $0.00 | $0.00 | $116.56 | $5,523.86 | 0.00 | 0.00 | 11.45 |
| 16 | Early | 0.001% | $0.00 | $0.00 | $116.56 | $5,523.86 | 0.00 | 0.00 | 11.45 |
| 16 | Advanced | 0.000% | $0.00 | $0.00 | $116.56 | $5,523.86 | 0.00 | 0.00 | 11.45 |
| 16 | Noncancerous | 0.148% | $0.00 | $0.00 | $116.56 | $5,523.86 | 0.00 | 0.00 | 11.45 |
| 16 | comply | 0.105% | $0.00 | $0.00 | $116.56 | $5,523.86 | 0.00 | 0.00 | 11.45 |
| 16 | Not comply | 0.043% | $0.00 | $0.00 | $116.56 | $5,523.86 | 0.00 | 0.00 | 11.45 |
| 16 | No adenoma | 0.195% | $0.00 | $0.00 | $116.56 | $5,523.86 | 0.00 | 0.00 | 11.45 |
| 16 | comply | 0.117% | $0.00 | $0.00 | $116.56 | $5,523.86 | 0.00 | 0.00 | 11.45 |
| 16 | Not comply | 0.077% | $0.00 | $0.00 | $116.56 | $5,523.86 | 0.00 | 0.00 | 11.45 |
| 16 | Continue | 0.020% | $0.00 | $0.00 | $116.56 | $5,523.86 | 0.00 | 0.00 | 11.45 |
| 16 | Die | 0.005% | $0.00 | $0.00 | $116.56 | $5,523.86 | 0.00 | 0.00 | 11.45 |
| 16 | PureVu usage | 3.204% | $0.00 | $0.00 | $116.56 | $5,523.86 | 0.00 | 0.00 | 11.45 |
| 16 | Later followup | 0.904% | $0.00 | $0.00 | $116.56 | $5,523.86 | 0.00 | 0.00 | 11.45 |
| 16 | Redo 2 years | 0.904% | $0.00 | $0.00 | $116.56 | $5,523.86 | 0.00 | 0.00 | 11.45 |
| 16 | comply | 0.505% | $0.00 | $0.00 | $116.56 | $5,523.86 | 0.00 | 0.00 | 11.45 |
| 16 | Not comply | 0.399% | $0.00 | $0.00 | $116.56 | $5,523.86 | 0.00 | 0.00 | 11.45 |
| 16 | No redo | 0.000% | $0.00 | $0.00 | $116.56 | $5,523.86 | 0.00 | 0.00 | 11.45 |
| 16 | Redo in at least 3 years | 0.000% | $0.00 | $0.00 | $116.56 | $5,523.86 | 0.00 | 0.00 | 11.45 |
| 16 | comply | 0.000% | $0.00 | $0.00 | $116.56 | $5,523.86 | 0.00 | 0.00 | 11.45 |
| 16 | Not comply | 0.000% | $0.00 | $0.00 | $116.56 | $5,523.86 | 0.00 | 0.00 | 11.45 |
| 16 | No redo | 0.000% | $0.00 | $0.00 | $116.56 | $5,523.86 | 0.00 | 0.00 | 11.45 |
| 16 | Adenoma | 0.000% | $0.00 | $0.00 | $116.56 | $5,523.86 | 0.00 | 0.00 | 11.45 |
| 16 | Cancerous | 0.000% | $0.00 | $0.00 | $116.56 | $5,523.86 | 0.00 | 0.00 | 11.45 |
| 16 | Early | 0.000% | $0.00 | $0.00 | $116.56 | $5,523.86 | 0.00 | 0.00 | 11.45 |
| 16 | Advanced | 0.000% | $0.00 | $0.00 | $116.56 | $5,523.86 | 0.00 | 0.00 | 11.45 |
| 16 | Noncancerous | 0.000% | $0.00 | $0.00 | $116.56 | $5,523.86 | 0.00 | 0.00 | 11.45 |
| 16 | No adenoma | 0.000% | $0.00 | $0.00 | $116.56 | $5,523.86 | 0.00 | 0.00 | 11.45 |
| 17 | comply | 56.284% | $0.00 | $0.00 | $109.08 | $5,632.95 | 0.54 | 0.31 | 11.99 |
| 17 | Non-comply post colonoscopy | 0.519% | $0.00 | $0.00 | $109.08 | $5,632.95 | 0.00 | 0.00 | 11.99 |
| 17 | Early CRC | 2.853% | $2,677.20 | $76.39 | $109.08 | $5,632.95 | 0.45 | 0.01 | 11.99 |
| 17 | Advanced CRC | 0.020% | $3,605.90 | $0.73 | $109.08 | $5,632.95 | 0.32 | 0.00 | 11.99 |
| 17 | Die other causes | 36.636% | $0.00 | $0.00 | $109.08 | $5,632.95 | 0.55 | 0.20 | 11.99 |
| 17 | Colonoscopy with PureVu due added to inadequate prep or not comply | 3.204% | $453.76 | $14.54 | $109.08 | $5,632.95 | 0.55 | 0.02 | 11.99 |
| 17 | Adenoma surveillance | 0.000% | $1,846.03 | $0.00 | $109.08 | $5,632.95 | 0.55 | 0.00 | 11.99 |
| 17 | Non-compliance with system | 0.000% | $0.00 | $0.00 | $109.08 | $5,632.95 | 0.00 | 0.00 | 11.99 |
| 17 | Dead | 0.483% | $3,605.90 | $17.43 | $109.08 | $5,632.95 | 0.00 | 0.00 | 11.99 |
| 17 | Screening every 10 years - average risk | 0.000% | $0.00 | $0.00 | $109.08 | $5,632.95 | 0.00 | 0.00 | 11.99 |
| 17 | Adequate Prep | 0.000% | $0.00 | $0.00 | $109.08 | $5,632.95 | 0.00 | 0.00 | 11.99 |
| 17 | no adenoma screening | 0.000% | $0.00 | $0.00 | $109.08 | $5,632.95 | 0.00 | 0.00 | 11.99 |
| 17 | Colonoscopy TN | 0.000% | $0.00 | $0.00 | $109.08 | $5,632.95 | 0.00 | 0.00 | 11.99 |
| 17 | Comply | 0.000% | $0.00 | $0.00 | $109.08 | $5,632.95 | 0.00 | 0.00 | 11.99 |
| 17 | Non-comply | 0.000% | $0.00 | $0.00 | $109.08 | $5,632.95 | 0.00 | 0.00 | 11.99 |
| 17 | Colonoscopy FP | 0.000% | $0.00 | $0.00 | $109.08 | $5,632.95 | 0.00 | 0.00 | 11.99 |
| 17 | adenoma | 0.000% | $0.00 | $0.00 | $109.08 | $5,632.95 | 0.00 | 0.00 | 11.99 |
| 17 | Distal Colon (includes descending, sigmoid colon, splenic flexure, rectum) | 0.000% | $0.00 | $0.00 | $109.08 | $5,632.95 | 0.00 | 0.00 | 11.99 |
| 17 | Colonoscopy TP | 0.000% | $0.00 | $0.00 | $109.08 | $5,632.95 | 0.00 | 0.00 | 11.99 |
| 17 | Colonoscopy FN | 0.000% | $0.00 | $0.00 | $109.08 | $5,632.95 | 0.00 | 0.00 | 11.99 |
| 17 | cancer | 0.000% | $0.00 | $0.00 | $109.08 | $5,632.95 | 0.00 | 0.00 | 11.99 |
| 17 | early | 0.000% | $0.00 | $0.00 | $109.08 | $5,632.95 | 0.00 | 0.00 | 11.99 |
| 17 | late | 0.000% | $0.00 | $0.00 | $109.08 | $5,632.95 | 0.00 | 0.00 | 11.99 |
| 17 | no cancer | 0.000% | $0.00 | $0.00 | $109.08 | $5,632.95 | 0.00 | 0.00 | 11.99 |
| 17 | Comply | 0.000% | $0.00 | $0.00 | $109.08 | $5,632.95 | 0.00 | 0.00 | 11.99 |
| 17 | Non-comply | 0.000% | $0.00 | $0.00 | $109.08 | $5,632.95 | 0.00 | 0.00 | 11.99 |
| 17 | Proximal colon (includes ascending, transverse) | 0.000% | $0.00 | $0.00 | $109.08 | $5,632.95 | 0.00 | 0.00 | 11.99 |
| 17 | Colonoscopy TP | 0.000% | $0.00 | $0.00 | $109.08 | $5,632.95 | 0.00 | 0.00 | 11.99 |
| 17 | Colonoscopy FN | 0.000% | $0.00 | $0.00 | $109.08 | $5,632.95 | 0.00 | 0.00 | 11.99 |
| 17 | cancer | 0.000% | $0.00 | $0.00 | $109.08 | $5,632.95 | 0.00 | 0.00 | 11.99 |
| 17 | early | 0.000% | $0.00 | $0.00 | $109.08 | $5,632.95 | 0.00 | 0.00 | 11.99 |
| 17 | late | 0.000% | $0.00 | $0.00 | $109.08 | $5,632.95 | 0.00 | 0.00 | 11.99 |
| 17 | no cancer | 0.000% | $0.00 | $0.00 | $109.08 | $5,632.95 | 0.00 | 0.00 | 11.99 |
| 17 | Comply | 0.000% | $0.00 | $0.00 | $109.08 | $5,632.95 | 0.00 | 0.00 | 11.99 |
| 17 | Non-comply | 0.000% | $0.00 | $0.00 | $109.08 | $5,632.95 | 0.00 | 0.00 | 11.99 |
| 17 | Inadequate Prep | 0.000% | $0.00 | $0.00 | $109.08 | $5,632.95 | 0.00 | 0.00 | 11.99 |
| 17 | PureVu usage | 0.000% | $0.00 | $0.00 | $109.08 | $5,632.95 | 0.00 | 0.00 | 11.99 |
| 17 | Colonoscopy FN | 0.000% | $0.00 | $0.00 | $109.08 | $5,632.95 | 0.00 | 0.00 | 11.99 |
| 17 | cancer | 0.000% | $0.00 | $0.00 | $109.08 | $5,632.95 | 0.00 | 0.00 | 11.99 |
| 17 | early | 0.000% | $0.00 | $0.00 | $109.08 | $5,632.95 | 0.00 | 0.00 | 11.99 |
| 17 | late | 0.000% | $0.00 | $0.00 | $109.08 | $5,632.95 | 0.00 | 0.00 | 11.99 |
| 17 | no cancer | 0.000% | $0.00 | $0.00 | $109.08 | $5,632.95 | 0.00 | 0.00 | 11.99 |
| 17 | Comply | 0.000% | $0.00 | $0.00 | $109.08 | $5,632.95 | 0.00 | 0.00 | 11.99 |
| 17 | Non-comply | 0.000% | $0.00 | $0.00 | $109.08 | $5,632.95 | 0.00 | 0.00 | 11.99 |
| 17 | No screening | 56.284% | $0.00 | $0.00 | $109.08 | $5,632.95 | 0.00 | 0.00 | 11.99 |
| 17 | Adenoma | 0.225% | $0.00 | $0.00 | $109.08 | $5,632.95 | 0.00 | 0.00 | 11.99 |
| 17 | Cancerous | 0.001% | $0.00 | $0.00 | $109.08 | $5,632.95 | 0.00 | 0.00 | 11.99 |
| 17 | Early | 0.001% | $0.00 | $0.00 | $109.08 | $5,632.95 | 0.00 | 0.00 | 11.99 |
| 17 | Advanced | 0.000% | $0.00 | $0.00 | $109.08 | $5,632.95 | 0.00 | 0.00 | 11.99 |
| 17 | Noncancerous | 0.224% | $0.00 | $0.00 | $109.08 | $5,632.95 | 0.00 | 0.00 | 11.99 |
| 17 | comply | 0.159% | $0.00 | $0.00 | $109.08 | $5,632.95 | 0.00 | 0.00 | 11.99 |
| 17 | Not comply | 0.065% | $0.00 | $0.00 | $109.08 | $5,632.95 | 0.00 | 0.00 | 11.99 |
| 17 | No adenoma | 0.294% | $0.00 | $0.00 | $109.08 | $5,632.95 | 0.00 | 0.00 | 11.99 |
| 17 | comply | 0.177% | $0.00 | $0.00 | $109.08 | $5,632.95 | 0.00 | 0.00 | 11.99 |
| 17 | Not comply | 0.117% | $0.00 | $0.00 | $109.08 | $5,632.95 | 0.00 | 0.00 | 11.99 |
| 17 | Continue | 0.016% | $0.00 | $0.00 | $109.08 | $5,632.95 | 0.00 | 0.00 | 11.99 |
| 17 | Die | 0.004% | $0.00 | $0.00 | $109.08 | $5,632.95 | 0.00 | 0.00 | 11.99 |
| 17 | PureVu usage | 2.499% | $0.00 | $0.00 | $109.08 | $5,632.95 | 0.00 | 0.00 | 11.99 |
| 17 | Later followup | 0.705% | $0.00 | $0.00 | $109.08 | $5,632.95 | 0.00 | 0.00 | 11.99 |
| 17 | Redo 2 years | 0.000% | $0.00 | $0.00 | $109.08 | $5,632.95 | 0.00 | 0.00 | 11.99 |
| 17 | comply | 0.000% | $0.00 | $0.00 | $109.08 | $5,632.95 | 0.00 | 0.00 | 11.99 |
| 17 | Not comply | 0.000% | $0.00 | $0.00 | $109.08 | $5,632.95 | 0.00 | 0.00 | 11.99 |
| 17 | No redo | 0.705% | $0.00 | $0.00 | $109.08 | $5,632.95 | 0.00 | 0.00 | 11.99 |
| 17 | Redo in at least 3 years | 0.000% | $0.00 | $0.00 | $109.08 | $5,632.95 | 0.00 | 0.00 | 11.99 |
| 17 | comply | 0.000% | $0.00 | $0.00 | $109.08 | $5,632.95 | 0.00 | 0.00 | 11.99 |
| 17 | Not comply | 0.000% | $0.00 | $0.00 | $109.08 | $5,632.95 | 0.00 | 0.00 | 11.99 |
| 17 | No redo | 0.000% | $0.00 | $0.00 | $109.08 | $5,632.95 | 0.00 | 0.00 | 11.99 |
| 17 | Adenoma | 0.000% | $0.00 | $0.00 | $109.08 | $5,632.95 | 0.00 | 0.00 | 11.99 |
| 17 | Cancerous | 0.000% | $0.00 | $0.00 | $109.08 | $5,632.95 | 0.00 | 0.00 | 11.99 |
| 17 | Early | 0.000% | $0.00 | $0.00 | $109.08 | $5,632.95 | 0.00 | 0.00 | 11.99 |
| 17 | Advanced | 0.000% | $0.00 | $0.00 | $109.08 | $5,632.95 | 0.00 | 0.00 | 11.99 |
| 17 | Noncancerous | 0.000% | $0.00 | $0.00 | $109.08 | $5,632.95 | 0.00 | 0.00 | 11.99 |
| 17 | No adenoma | 0.000% | $0.00 | $0.00 | $109.08 | $5,632.95 | 0.00 | 0.00 | 11.99 |
| 18 | comply | 57.325% | $0.00 | $0.00 | $102.83 | $5,735.77 | 0.53 | 0.30 | 12.51 |
| 18 | Non-comply post colonoscopy | 0.182% | $0.00 | $0.00 | $102.83 | $5,735.77 | 0.00 | 0.00 | 12.51 |
| 18 | Early CRC | 2.854% | $2,599.22 | $74.18 | $102.83 | $5,735.77 | 0.43 | 0.01 | 12.51 |
| 18 | Advanced CRC | 0.016% | $3,500.87 | $0.57 | $102.83 | $5,735.77 | 0.31 | 0.00 | 12.51 |
| 18 | Die other causes | 36.636% | $0.00 | $0.00 | $102.83 | $5,735.77 | 0.53 | 0.20 | 12.51 |
| 18 | Colonoscopy with PureVu due added to inadequate prep or not comply | 2.499% | $440.55 | $11.01 | $102.83 | $5,735.77 | 0.53 | 0.01 | 12.51 |
| 18 | Adenoma surveillance | 0.000% | $1,792.26 | $0.00 | $102.83 | $5,735.77 | 0.53 | 0.00 | 12.51 |
| 18 | Non-compliance with system | 0.000% | $0.00 | $0.00 | $102.83 | $5,735.77 | 0.00 | 0.00 | 12.51 |
| 18 | Dead | 0.487% | $3,500.87 | $17.06 | $102.83 | $5,735.77 | 0.00 | 0.00 | 12.51 |
| 18 | Screening every 10 years - average risk | 0.000% | $0.00 | $0.00 | $102.83 | $5,735.77 | 0.00 | 0.00 | 12.51 |
| 18 | Adequate Prep | 0.000% | $0.00 | $0.00 | $102.83 | $5,735.77 | 0.00 | 0.00 | 12.51 |
| 18 | no adenoma screening | 0.000% | $0.00 | $0.00 | $102.83 | $5,735.77 | 0.00 | 0.00 | 12.51 |
| 18 | Colonoscopy TN | 0.000% | $0.00 | $0.00 | $102.83 | $5,735.77 | 0.00 | 0.00 | 12.51 |
| 18 | Comply | 0.000% | $0.00 | $0.00 | $102.83 | $5,735.77 | 0.00 | 0.00 | 12.51 |
| 18 | Non-comply | 0.000% | $0.00 | $0.00 | $102.83 | $5,735.77 | 0.00 | 0.00 | 12.51 |
| 18 | Colonoscopy FP | 0.000% | $0.00 | $0.00 | $102.83 | $5,735.77 | 0.00 | 0.00 | 12.51 |
| 18 | adenoma | 0.000% | $0.00 | $0.00 | $102.83 | $5,735.77 | 0.00 | 0.00 | 12.51 |
| 18 | Distal Colon (includes descending, sigmoid colon, splenic flexure, rectum) | 0.000% | $0.00 | $0.00 | $102.83 | $5,735.77 | 0.00 | 0.00 | 12.51 |
| 18 | Colonoscopy TP | 0.000% | $0.00 | $0.00 | $102.83 | $5,735.77 | 0.00 | 0.00 | 12.51 |
| 18 | Colonoscopy FN | 0.000% | $0.00 | $0.00 | $102.83 | $5,735.77 | 0.00 | 0.00 | 12.51 |
| 18 | cancer | 0.000% | $0.00 | $0.00 | $102.83 | $5,735.77 | 0.00 | 0.00 | 12.51 |
| 18 | early | 0.000% | $0.00 | $0.00 | $102.83 | $5,735.77 | 0.00 | 0.00 | 12.51 |
| 18 | late | 0.000% | $0.00 | $0.00 | $102.83 | $5,735.77 | 0.00 | 0.00 | 12.51 |
| 18 | no cancer | 0.000% | $0.00 | $0.00 | $102.83 | $5,735.77 | 0.00 | 0.00 | 12.51 |
| 18 | Comply | 0.000% | $0.00 | $0.00 | $102.83 | $5,735.77 | 0.00 | 0.00 | 12.51 |
| 18 | Non-comply | 0.000% | $0.00 | $0.00 | $102.83 | $5,735.77 | 0.00 | 0.00 | 12.51 |
| 18 | Proximal colon (includes ascending, transverse) | 0.000% | $0.00 | $0.00 | $102.83 | $5,735.77 | 0.00 | 0.00 | 12.51 |
| 18 | Colonoscopy TP | 0.000% | $0.00 | $0.00 | $102.83 | $5,735.77 | 0.00 | 0.00 | 12.51 |
| 18 | Colonoscopy FN | 0.000% | $0.00 | $0.00 | $102.83 | $5,735.77 | 0.00 | 0.00 | 12.51 |
| 18 | cancer | 0.000% | $0.00 | $0.00 | $102.83 | $5,735.77 | 0.00 | 0.00 | 12.51 |
| 18 | early | 0.000% | $0.00 | $0.00 | $102.83 | $5,735.77 | 0.00 | 0.00 | 12.51 |
| 18 | late | 0.000% | $0.00 | $0.00 | $102.83 | $5,735.77 | 0.00 | 0.00 | 12.51 |
| 18 | no cancer | 0.000% | $0.00 | $0.00 | $102.83 | $5,735.77 | 0.00 | 0.00 | 12.51 |
| 18 | Comply | 0.000% | $0.00 | $0.00 | $102.83 | $5,735.77 | 0.00 | 0.00 | 12.51 |
| 18 | Non-comply | 0.000% | $0.00 | $0.00 | $102.83 | $5,735.77 | 0.00 | 0.00 | 12.51 |
| 18 | Inadequate Prep | 0.000% | $0.00 | $0.00 | $102.83 | $5,735.77 | 0.00 | 0.00 | 12.51 |
| 18 | PureVu usage | 0.000% | $0.00 | $0.00 | $102.83 | $5,735.77 | 0.00 | 0.00 | 12.51 |
| 18 | Colonoscopy FN | 0.000% | $0.00 | $0.00 | $102.83 | $5,735.77 | 0.00 | 0.00 | 12.51 |
| 18 | cancer | 0.000% | $0.00 | $0.00 | $102.83 | $5,735.77 | 0.00 | 0.00 | 12.51 |
| 18 | early | 0.000% | $0.00 | $0.00 | $102.83 | $5,735.77 | 0.00 | 0.00 | 12.51 |
| 18 | late | 0.000% | $0.00 | $0.00 | $102.83 | $5,735.77 | 0.00 | 0.00 | 12.51 |
| 18 | no cancer | 0.000% | $0.00 | $0.00 | $102.83 | $5,735.77 | 0.00 | 0.00 | 12.51 |
| 18 | Comply | 0.000% | $0.00 | $0.00 | $102.83 | $5,735.77 | 0.00 | 0.00 | 12.51 |
| 18 | Non-comply | 0.000% | $0.00 | $0.00 | $102.83 | $5,735.77 | 0.00 | 0.00 | 12.51 |
| 18 | No screening | 57.325% | $0.00 | $0.00 | $102.83 | $5,735.77 | 0.00 | 0.00 | 12.51 |
| 18 | Adenoma | 0.079% | $0.00 | $0.00 | $102.83 | $5,735.77 | 0.00 | 0.00 | 12.51 |
| 18 | Cancerous | 0.000% | $0.00 | $0.00 | $102.83 | $5,735.77 | 0.00 | 0.00 | 12.51 |
| 18 | Early | 0.000% | $0.00 | $0.00 | $102.83 | $5,735.77 | 0.00 | 0.00 | 12.51 |
| 18 | Advanced | 0.000% | $0.00 | $0.00 | $102.83 | $5,735.77 | 0.00 | 0.00 | 12.51 |
| 18 | Noncancerous | 0.078% | $0.00 | $0.00 | $102.83 | $5,735.77 | 0.00 | 0.00 | 12.51 |
| 18 | comply | 0.056% | $0.00 | $0.00 | $102.83 | $5,735.77 | 0.00 | 0.00 | 12.51 |
| 18 | Not comply | 0.023% | $0.00 | $0.00 | $102.83 | $5,735.77 | 0.00 | 0.00 | 12.51 |
| 18 | No adenoma | 0.103% | $0.00 | $0.00 | $102.83 | $5,735.77 | 0.00 | 0.00 | 12.51 |
| 18 | comply | 0.062% | $0.00 | $0.00 | $102.83 | $5,735.77 | 0.00 | 0.00 | 12.51 |
| 18 | Not comply | 0.041% | $0.00 | $0.00 | $102.83 | $5,735.77 | 0.00 | 0.00 | 12.51 |
| 18 | Continue | 0.013% | $0.00 | $0.00 | $102.83 | $5,735.77 | 0.00 | 0.00 | 12.51 |
| 18 | Die | 0.003% | $0.00 | $0.00 | $102.83 | $5,735.77 | 0.00 | 0.00 | 12.51 |
| 18 | PureVu usage | 1.950% | $0.00 | $0.00 | $102.83 | $5,735.77 | 0.00 | 0.00 | 12.51 |
| 18 | Later followup | 0.550% | $0.00 | $0.00 | $102.83 | $5,735.77 | 0.00 | 0.00 | 12.51 |
| 18 | Redo 2 years | 0.550% | $0.00 | $0.00 | $102.83 | $5,735.77 | 0.00 | 0.00 | 12.51 |
| 18 | comply | 0.307% | $0.00 | $0.00 | $102.83 | $5,735.77 | 0.00 | 0.00 | 12.51 |
| 18 | Not comply | 0.242% | $0.00 | $0.00 | $102.83 | $5,735.77 | 0.00 | 0.00 | 12.51 |
| 18 | No redo | 0.000% | $0.00 | $0.00 | $102.83 | $5,735.77 | 0.00 | 0.00 | 12.51 |
| 18 | Redo in at least 3 years | 0.000% | $0.00 | $0.00 | $102.83 | $5,735.77 | 0.00 | 0.00 | 12.51 |
| 18 | comply | 0.000% | $0.00 | $0.00 | $102.83 | $5,735.77 | 0.00 | 0.00 | 12.51 |
| 18 | Not comply | 0.000% | $0.00 | $0.00 | $102.83 | $5,735.77 | 0.00 | 0.00 | 12.51 |
| 18 | No redo | 0.000% | $0.00 | $0.00 | $102.83 | $5,735.77 | 0.00 | 0.00 | 12.51 |
| 18 | Adenoma | 0.000% | $0.00 | $0.00 | $102.83 | $5,735.77 | 0.00 | 0.00 | 12.51 |
| 18 | Cancerous | 0.000% | $0.00 | $0.00 | $102.83 | $5,735.77 | 0.00 | 0.00 | 12.51 |
| 18 | Early | 0.000% | $0.00 | $0.00 | $102.83 | $5,735.77 | 0.00 | 0.00 | 12.51 |
| 18 | Advanced | 0.000% | $0.00 | $0.00 | $102.83 | $5,735.77 | 0.00 | 0.00 | 12.51 |
| 18 | Noncancerous | 0.000% | $0.00 | $0.00 | $102.83 | $5,735.77 | 0.00 | 0.00 | 12.51 |
| 18 | No adenoma | 0.000% | $0.00 | $0.00 | $102.83 | $5,735.77 | 0.00 | 0.00 | 12.51 |
| 19 | comply | 57.750% | $0.00 | $0.00 | $97.49 | $5,833.26 | 0.51 | 0.30 | 13.02 |
| 19 | Non-comply post colonoscopy | 0.306% | $0.00 | $0.00 | $97.49 | $5,833.26 | 0.00 | 0.00 | 13.02 |
| 19 | Early CRC | 2.854% | $2,523.52 | $72.03 | $97.49 | $5,833.26 | 0.42 | 0.01 | 13.02 |
| 19 | Advanced CRC | 0.013% | $3,398.90 | $0.45 | $97.49 | $5,833.26 | 0.30 | 0.00 | 13.02 |
| 19 | Die other causes | 36.636% | $0.00 | $0.00 | $97.49 | $5,833.26 | 0.52 | 0.19 | 13.02 |
| 19 | Colonoscopy with PureVu due added to inadequate prep or not comply | 1.950% | $427.71 | $8.34 | $97.49 | $5,833.26 | 0.52 | 0.01 | 13.02 |
| 19 | Adenoma surveillance | 0.000% | $1,740.06 | $0.00 | $97.49 | $5,833.26 | 0.52 | 0.00 | 13.02 |
| 19 | Non-compliance with system | 0.000% | $0.00 | $0.00 | $97.49 | $5,833.26 | 0.00 | 0.00 | 13.02 |
| 19 | Dead | 0.491% | $3,398.90 | $16.67 | $97.49 | $5,833.26 | 0.00 | 0.00 | 13.02 |
| 19 | Screening every 10 years - average risk | 0.000% | $0.00 | $0.00 | $97.49 | $5,833.26 | 0.00 | 0.00 | 13.02 |
| 19 | Adequate Prep | 0.000% | $0.00 | $0.00 | $97.49 | $5,833.26 | 0.00 | 0.00 | 13.02 |
| 19 | no adenoma screening | 0.000% | $0.00 | $0.00 | $97.49 | $5,833.26 | 0.00 | 0.00 | 13.02 |
| 19 | Colonoscopy TN | 0.000% | $0.00 | $0.00 | $97.49 | $5,833.26 | 0.00 | 0.00 | 13.02 |
| 19 | Comply | 0.000% | $0.00 | $0.00 | $97.49 | $5,833.26 | 0.00 | 0.00 | 13.02 |
| 19 | Non-comply | 0.000% | $0.00 | $0.00 | $97.49 | $5,833.26 | 0.00 | 0.00 | 13.02 |
| 19 | Colonoscopy FP | 0.000% | $0.00 | $0.00 | $97.49 | $5,833.26 | 0.00 | 0.00 | 13.02 |
| 19 | adenoma | 0.000% | $0.00 | $0.00 | $97.49 | $5,833.26 | 0.00 | 0.00 | 13.02 |
| 19 | Distal Colon (includes descending, sigmoid colon, splenic flexure, rectum) | 0.000% | $0.00 | $0.00 | $97.49 | $5,833.26 | 0.00 | 0.00 | 13.02 |
| 19 | Colonoscopy TP | 0.000% | $0.00 | $0.00 | $97.49 | $5,833.26 | 0.00 | 0.00 | 13.02 |
| 19 | Colonoscopy FN | 0.000% | $0.00 | $0.00 | $97.49 | $5,833.26 | 0.00 | 0.00 | 13.02 |
| 19 | cancer | 0.000% | $0.00 | $0.00 | $97.49 | $5,833.26 | 0.00 | 0.00 | 13.02 |
| 19 | early | 0.000% | $0.00 | $0.00 | $97.49 | $5,833.26 | 0.00 | 0.00 | 13.02 |
| 19 | late | 0.000% | $0.00 | $0.00 | $97.49 | $5,833.26 | 0.00 | 0.00 | 13.02 |
| 19 | no cancer | 0.000% | $0.00 | $0.00 | $97.49 | $5,833.26 | 0.00 | 0.00 | 13.02 |
| 19 | Comply | 0.000% | $0.00 | $0.00 | $97.49 | $5,833.26 | 0.00 | 0.00 | 13.02 |
| 19 | Non-comply | 0.000% | $0.00 | $0.00 | $97.49 | $5,833.26 | 0.00 | 0.00 | 13.02 |
| 19 | Proximal colon (includes ascending, transverse) | 0.000% | $0.00 | $0.00 | $97.49 | $5,833.26 | 0.00 | 0.00 | 13.02 |
| 19 | Colonoscopy TP | 0.000% | $0.00 | $0.00 | $97.49 | $5,833.26 | 0.00 | 0.00 | 13.02 |
| 19 | Colonoscopy FN | 0.000% | $0.00 | $0.00 | $97.49 | $5,833.26 | 0.00 | 0.00 | 13.02 |
| 19 | cancer | 0.000% | $0.00 | $0.00 | $97.49 | $5,833.26 | 0.00 | 0.00 | 13.02 |
| 19 | early | 0.000% | $0.00 | $0.00 | $97.49 | $5,833.26 | 0.00 | 0.00 | 13.02 |
| 19 | late | 0.000% | $0.00 | $0.00 | $97.49 | $5,833.26 | 0.00 | 0.00 | 13.02 |
| 19 | no cancer | 0.000% | $0.00 | $0.00 | $97.49 | $5,833.26 | 0.00 | 0.00 | 13.02 |
| 19 | Comply | 0.000% | $0.00 | $0.00 | $97.49 | $5,833.26 | 0.00 | 0.00 | 13.02 |
| 19 | Non-comply | 0.000% | $0.00 | $0.00 | $97.49 | $5,833.26 | 0.00 | 0.00 | 13.02 |
| 19 | Inadequate Prep | 0.000% | $0.00 | $0.00 | $97.49 | $5,833.26 | 0.00 | 0.00 | 13.02 |
| 19 | PureVu usage | 0.000% | $0.00 | $0.00 | $97.49 | $5,833.26 | 0.00 | 0.00 | 13.02 |
| 19 | Colonoscopy FN | 0.000% | $0.00 | $0.00 | $97.49 | $5,833.26 | 0.00 | 0.00 | 13.02 |
| 19 | cancer | 0.000% | $0.00 | $0.00 | $97.49 | $5,833.26 | 0.00 | 0.00 | 13.02 |
| 19 | early | 0.000% | $0.00 | $0.00 | $97.49 | $5,833.26 | 0.00 | 0.00 | 13.02 |
| 19 | late | 0.000% | $0.00 | $0.00 | $97.49 | $5,833.26 | 0.00 | 0.00 | 13.02 |
| 19 | no cancer | 0.000% | $0.00 | $0.00 | $97.49 | $5,833.26 | 0.00 | 0.00 | 13.02 |
| 19 | Comply | 0.000% | $0.00 | $0.00 | $97.49 | $5,833.26 | 0.00 | 0.00 | 13.02 |
| 19 | Non-comply | 0.000% | $0.00 | $0.00 | $97.49 | $5,833.26 | 0.00 | 0.00 | 13.02 |
| 19 | No screening | 57.750% | $0.00 | $0.00 | $97.49 | $5,833.26 | 0.00 | 0.00 | 13.02 |
| 19 | Adenoma | 0.133% | $0.00 | $0.00 | $97.49 | $5,833.26 | 0.00 | 0.00 | 13.02 |
| 19 | Cancerous | 0.001% | $0.00 | $0.00 | $97.49 | $5,833.26 | 0.00 | 0.00 | 13.02 |
| 19 | Early | 0.000% | $0.00 | $0.00 | $97.49 | $5,833.26 | 0.00 | 0.00 | 13.02 |
| 19 | Advanced | 0.000% | $0.00 | $0.00 | $97.49 | $5,833.26 | 0.00 | 0.00 | 13.02 |
| 19 | Noncancerous | 0.132% | $0.00 | $0.00 | $97.49 | $5,833.26 | 0.00 | 0.00 | 13.02 |
| 19 | comply | 0.094% | $0.00 | $0.00 | $97.49 | $5,833.26 | 0.00 | 0.00 | 13.02 |
| 19 | Not comply | 0.038% | $0.00 | $0.00 | $97.49 | $5,833.26 | 0.00 | 0.00 | 13.02 |
| 19 | No adenoma | 0.173% | $0.00 | $0.00 | $97.49 | $5,833.26 | 0.00 | 0.00 | 13.02 |
| 19 | comply | 0.105% | $0.00 | $0.00 | $97.49 | $5,833.26 | 0.00 | 0.00 | 13.02 |
| 19 | Not comply | 0.069% | $0.00 | $0.00 | $97.49 | $5,833.26 | 0.00 | 0.00 | 13.02 |
| 19 | Continue | 0.011% | $0.00 | $0.00 | $97.49 | $5,833.26 | 0.00 | 0.00 | 13.02 |
| 19 | Die | 0.003% | $0.00 | $0.00 | $97.49 | $5,833.26 | 0.00 | 0.00 | 13.02 |
| 19 | PureVu usage | 1.521% | $0.00 | $0.00 | $97.49 | $5,833.26 | 0.00 | 0.00 | 13.02 |
| 19 | Later followup | 0.429% | $0.00 | $0.00 | $97.49 | $5,833.26 | 0.00 | 0.00 | 13.02 |
| 19 | Redo 2 years | 0.000% | $0.00 | $0.00 | $97.49 | $5,833.26 | 0.00 | 0.00 | 13.02 |
| 19 | comply | 0.000% | $0.00 | $0.00 | $97.49 | $5,833.26 | 0.00 | 0.00 | 13.02 |
| 19 | Not comply | 0.000% | $0.00 | $0.00 | $97.49 | $5,833.26 | 0.00 | 0.00 | 13.02 |
| 19 | No redo | 0.429% | $0.00 | $0.00 | $97.49 | $5,833.26 | 0.00 | 0.00 | 13.02 |
| 19 | Redo in at least 3 years | 0.000% | $0.00 | $0.00 | $97.49 | $5,833.26 | 0.00 | 0.00 | 13.02 |
| 19 | comply | 0.000% | $0.00 | $0.00 | $97.49 | $5,833.26 | 0.00 | 0.00 | 13.02 |
| 19 | Not comply | 0.000% | $0.00 | $0.00 | $97.49 | $5,833.26 | 0.00 | 0.00 | 13.02 |
| 19 | No redo | 0.000% | $0.00 | $0.00 | $97.49 | $5,833.26 | 0.00 | 0.00 | 13.02 |
| 19 | Adenoma | 0.000% | $0.00 | $0.00 | $97.49 | $5,833.26 | 0.00 | 0.00 | 13.02 |
| 19 | Cancerous | 0.000% | $0.00 | $0.00 | $97.49 | $5,833.26 | 0.00 | 0.00 | 13.02 |
| 19 | Early | 0.000% | $0.00 | $0.00 | $97.49 | $5,833.26 | 0.00 | 0.00 | 13.02 |
| 19 | Advanced | 0.000% | $0.00 | $0.00 | $97.49 | $5,833.26 | 0.00 | 0.00 | 13.02 |
| 19 | Noncancerous | 0.000% | $0.00 | $0.00 | $97.49 | $5,833.26 | 0.00 | 0.00 | 13.02 |
| 19 | No adenoma | 0.000% | $0.00 | $0.00 | $97.49 | $5,833.26 | 0.00 | 0.00 | 13.02 |
| 20 | comply | 58.377% | $1,219.38 | $711.84 | $804.72 | $6,637.99 | 0.50 | 0.29 | 13.51 |
| 20 | Non-comply post colonoscopy | 0.107% | $0.00 | $0.00 | $804.72 | $6,637.99 | 0.00 | 0.00 | 13.51 |
| 20 | Early CRC | 2.855% | $2,450.02 | $69.94 | $804.72 | $6,637.99 | 0.41 | 0.01 | 13.51 |
| 20 | Advanced CRC | 0.011% | $3,299.91 | $0.35 | $804.72 | $6,637.99 | 0.29 | 0.00 | 13.51 |
| 20 | Die other causes | 36.636% | $0.00 | $0.00 | $804.72 | $6,637.99 | 0.50 | 0.18 | 13.51 |
| 20 | Colonoscopy with PureVu due added to inadequate prep or not comply | 1.521% | $415.26 | $6.31 | $804.72 | $6,637.99 | 0.50 | 0.01 | 13.51 |
| 20 | Adenoma surveillance | 0.000% | $1,689.38 | $0.00 | $804.72 | $6,637.99 | 0.50 | 0.00 | 13.51 |
| 20 | Non-compliance with system | 0.000% | $0.00 | $0.00 | $804.72 | $6,637.99 | 0.00 | 0.00 | 13.51 |
| 20 | Dead | 0.493% | $3,299.91 | $16.28 | $804.72 | $6,637.99 | 0.00 | 0.00 | 13.51 |
| 20 | Screening every 10 years - average risk | 58.377% | $0.00 | $0.00 | $804.72 | $6,637.99 | 0.00 | 0.00 | 13.51 |
| 20 | Adequate Prep | 41.643% | $0.00 | $0.00 | $804.72 | $6,637.99 | 0.00 | 0.00 | 13.51 |
| 20 | no adenoma screening | 29.150% | $0.00 | $0.00 | $804.72 | $6,637.99 | 0.00 | 0.00 | 13.51 |
| 20 | Colonoscopy TN | 27.138% | $0.00 | $0.00 | $804.72 | $6,637.99 | 0.00 | 0.00 | 13.51 |
| 20 | Comply | 16.365% | $0.00 | $0.00 | $804.72 | $6,637.99 | 0.00 | 0.00 | 13.51 |
| 20 | Non-comply | 10.774% | $0.00 | $0.00 | $804.72 | $6,637.99 | 0.00 | 0.00 | 13.51 |
| 20 | Colonoscopy FP | 2.011% | $0.00 | $0.00 | $804.72 | $6,637.99 | 0.00 | 0.00 | 13.51 |
| 20 | adenoma | 12.493% | $0.00 | $0.00 | $804.72 | $6,637.99 | 0.00 | 0.00 | 13.51 |
| 20 | Distal Colon (includes descending, sigmoid colon, splenic flexure, rectum) | 6.421% | $0.00 | $0.00 | $804.72 | $6,637.99 | 0.00 | 0.00 | 13.51 |
| 20 | Colonoscopy TP | 5.779% | $0.00 | $0.00 | $804.72 | $6,637.99 | 0.00 | 0.00 | 13.51 |
| 20 | Colonoscopy FN | 0.642% | $0.00 | $0.00 | $804.72 | $6,637.99 | 0.00 | 0.00 | 13.51 |
| 20 | cancer | 0.003% | $0.00 | $0.00 | $804.72 | $6,637.99 | 0.00 | 0.00 | 13.51 |
| 20 | early | 0.002% | $0.00 | $0.00 | $804.72 | $6,637.99 | 0.00 | 0.00 | 13.51 |
| 20 | late | 0.000% | $0.00 | $0.00 | $804.72 | $6,637.99 | 0.00 | 0.00 | 13.51 |
| 20 | no cancer | 0.639% | $0.00 | $0.00 | $804.72 | $6,637.99 | 0.00 | 0.00 | 13.51 |
| 20 | Comply | 0.386% | $0.00 | $0.00 | $804.72 | $6,637.99 | 0.00 | 0.00 | 13.51 |
| 20 | Non-comply | 0.254% | $0.00 | $0.00 | $804.72 | $6,637.99 | 0.00 | 0.00 | 13.51 |
| 20 | Proximal colon (includes ascending, transverse) | 6.071% | $0.00 | $0.00 | $804.72 | $6,637.99 | 0.00 | 0.00 | 13.51 |
| 20 | Colonoscopy TP | 5.464% | $0.00 | $0.00 | $804.72 | $6,637.99 | 0.00 | 0.00 | 13.51 |
| 20 | Colonoscopy FN | 0.607% | $0.00 | $0.00 | $804.72 | $6,637.99 | 0.00 | 0.00 | 13.51 |
| 20 | cancer | 0.003% | $0.00 | $0.00 | $804.72 | $6,637.99 | 0.00 | 0.00 | 13.51 |
| 20 | early | 0.002% | $0.00 | $0.00 | $804.72 | $6,637.99 | 0.00 | 0.00 | 13.51 |
| 20 | late | 0.000% | $0.00 | $0.00 | $804.72 | $6,637.99 | 0.00 | 0.00 | 13.51 |
| 20 | no cancer | 0.605% | $0.00 | $0.00 | $804.72 | $6,637.99 | 0.00 | 0.00 | 13.51 |
| 20 | Comply | 0.365% | $0.00 | $0.00 | $804.72 | $6,637.99 | 0.00 | 0.00 | 13.51 |
| 20 | Non-comply | 0.240% | $0.00 | $0.00 | $804.72 | $6,637.99 | 0.00 | 0.00 | 13.51 |
| 20 | Inadequate Prep | 16.735% | $0.00 | $0.00 | $804.72 | $6,637.99 | 0.00 | 0.00 | 13.51 |
| 20 | PureVu usage | 13.053% | $0.00 | $0.00 | $804.72 | $6,637.99 | 0.00 | 0.00 | 13.51 |
| 20 | Colonoscopy FN | 3.682% | $0.00 | $0.00 | $804.72 | $6,637.99 | 0.00 | 0.00 | 13.51 |
| 20 | cancer | 0.097% | $0.00 | $0.00 | $804.72 | $6,637.99 | 0.00 | 0.00 | 13.51 |
| 20 | early | 0.082% | $0.00 | $0.00 | $804.72 | $6,637.99 | 0.00 | 0.00 | 13.51 |
| 20 | late | 0.015% | $0.00 | $0.00 | $804.72 | $6,637.99 | 0.00 | 0.00 | 13.51 |
| 20 | no cancer | 3.585% | $0.00 | $0.00 | $804.72 | $6,637.99 | 0.00 | 0.00 | 13.51 |
| 20 | Comply | 2.162% | $0.00 | $0.00 | $804.72 | $6,637.99 | 0.00 | 0.00 | 13.51 |
| 20 | Non-comply | 1.423% | $0.00 | $0.00 | $804.72 | $6,637.99 | 0.00 | 0.00 | 13.51 |
| 20 | No screening | 0.000% | $0.00 | $0.00 | $804.72 | $6,637.99 | 0.00 | 0.00 | 13.51 |
| 20 | Adenoma | 0.046% | $0.00 | $0.00 | $804.72 | $6,637.99 | 0.00 | 0.00 | 13.51 |
| 20 | Cancerous | 0.000% | $0.00 | $0.00 | $804.72 | $6,637.99 | 0.00 | 0.00 | 13.51 |
| 20 | Early | 0.000% | $0.00 | $0.00 | $804.72 | $6,637.99 | 0.00 | 0.00 | 13.51 |
| 20 | Advanced | 0.000% | $0.00 | $0.00 | $804.72 | $6,637.99 | 0.00 | 0.00 | 13.51 |
| 20 | Noncancerous | 0.046% | $0.00 | $0.00 | $804.72 | $6,637.99 | 0.00 | 0.00 | 13.51 |
| 20 | comply | 0.033% | $0.00 | $0.00 | $804.72 | $6,637.99 | 0.00 | 0.00 | 13.51 |
| 20 | Not comply | 0.013% | $0.00 | $0.00 | $804.72 | $6,637.99 | 0.00 | 0.00 | 13.51 |
| 20 | No adenoma | 0.061% | $0.00 | $0.00 | $804.72 | $6,637.99 | 0.00 | 0.00 | 13.51 |
| 20 | comply | 0.037% | $0.00 | $0.00 | $804.72 | $6,637.99 | 0.00 | 0.00 | 13.51 |
| 20 | Not comply | 0.024% | $0.00 | $0.00 | $804.72 | $6,637.99 | 0.00 | 0.00 | 13.51 |
| 20 | Continue | 0.008% | $0.00 | $0.00 | $804.72 | $6,637.99 | 0.00 | 0.00 | 13.51 |
| 20 | Die | 0.002% | $0.00 | $0.00 | $804.72 | $6,637.99 | 0.00 | 0.00 | 13.51 |
| 20 | PureVu usage | 1.186% | $0.00 | $0.00 | $804.72 | $6,637.99 | 0.00 | 0.00 | 13.51 |
| 20 | Later followup | 0.335% | $0.00 | $0.00 | $804.72 | $6,637.99 | 0.00 | 0.00 | 13.51 |
| 20 | Redo 2 years | 0.335% | $0.00 | $0.00 | $804.72 | $6,637.99 | 0.00 | 0.00 | 13.51 |
| 20 | comply | 0.187% | $0.00 | $0.00 | $804.72 | $6,637.99 | 0.00 | 0.00 | 13.51 |
| 20 | Not comply | 0.148% | $0.00 | $0.00 | $804.72 | $6,637.99 | 0.00 | 0.00 | 13.51 |
| 20 | No redo | 0.000% | $0.00 | $0.00 | $804.72 | $6,637.99 | 0.00 | 0.00 | 13.51 |
| 20 | Redo in at least 3 years | 0.000% | $0.00 | $0.00 | $804.72 | $6,637.99 | 0.00 | 0.00 | 13.51 |
| 20 | comply | 0.000% | $0.00 | $0.00 | $804.72 | $6,637.99 | 0.00 | 0.00 | 13.51 |
| 20 | Not comply | 0.000% | $0.00 | $0.00 | $804.72 | $6,637.99 | 0.00 | 0.00 | 13.51 |
| 20 | No redo | 0.000% | $0.00 | $0.00 | $804.72 | $6,637.99 | 0.00 | 0.00 | 13.51 |
| 20 | Adenoma | 0.000% | $0.00 | $0.00 | $804.72 | $6,637.99 | 0.00 | 0.00 | 13.51 |
| 20 | Cancerous | 0.000% | $0.00 | $0.00 | $804.72 | $6,637.99 | 0.00 | 0.00 | 13.51 |
| 20 | Early | 0.000% | $0.00 | $0.00 | $804.72 | $6,637.99 | 0.00 | 0.00 | 13.51 |
| 20 | Advanced | 0.000% | $0.00 | $0.00 | $804.72 | $6,637.99 | 0.00 | 0.00 | 13.51 |
| 20 | Noncancerous | 0.000% | $0.00 | $0.00 | $804.72 | $6,637.99 | 0.00 | 0.00 | 13.51 |
| 20 | No adenoma | 0.000% | $0.00 | $0.00 | $804.72 | $6,637.99 | 0.00 | 0.00 | 13.51 |
| 21 | comply | 19.533% | $0.00 | $0.00 | $361.42 | $6,999.41 | 0.48 | 0.09 | 13.93 |
| 21 | Non-comply post colonoscopy | 12.876% | $0.00 | $0.00 | $361.42 | $6,999.41 | 0.00 | 0.00 | 13.93 |
| 21 | Early CRC | 2.942% | $2,378.66 | $69.98 | $361.42 | $6,999.41 | 0.40 | 0.01 | 13.93 |
| 21 | Advanced CRC | 0.024% | $3,203.79 | $0.76 | $361.42 | $6,999.41 | 0.28 | 0.00 | 13.93 |
| 21 | Die other causes | 36.636% | $0.00 | $0.00 | $361.42 | $6,999.41 | 0.49 | 0.18 | 13.93 |
| 21 | Colonoscopy with PureVu due added to inadequate prep or not comply | 14.239% | $403.16 | $57.41 | $361.42 | $6,999.41 | 0.49 | 0.07 | 13.93 |
| 21 | Adenoma surveillance | 13.255% | $1,640.17 | $217.40 | $361.42 | $6,999.41 | 0.49 | 0.06 | 13.93 |
| 21 | Non-compliance with system | 0.000% | $0.00 | $0.00 | $361.42 | $6,999.41 | 0.00 | 0.00 | 13.93 |
| 21 | Dead | 0.495% | $3,203.79 | $15.87 | $361.42 | $6,999.41 | 0.00 | 0.00 | 13.93 |
| 21 | Screening every 10 years - average risk | 0.000% | $0.00 | $0.00 | $361.42 | $6,999.41 | 0.00 | 0.00 | 13.93 |
| 21 | Adequate Prep | 0.000% | $0.00 | $0.00 | $361.42 | $6,999.41 | 0.00 | 0.00 | 13.93 |
| 21 | no adenoma screening | 0.000% | $0.00 | $0.00 | $361.42 | $6,999.41 | 0.00 | 0.00 | 13.93 |
| 21 | Colonoscopy TN | 0.000% | $0.00 | $0.00 | $361.42 | $6,999.41 | 0.00 | 0.00 | 13.93 |
| 21 | Comply | 0.000% | $0.00 | $0.00 | $361.42 | $6,999.41 | 0.00 | 0.00 | 13.93 |
| 21 | Non-comply | 0.000% | $0.00 | $0.00 | $361.42 | $6,999.41 | 0.00 | 0.00 | 13.93 |
| 21 | Colonoscopy FP | 0.000% | $0.00 | $0.00 | $361.42 | $6,999.41 | 0.00 | 0.00 | 13.93 |
| 21 | adenoma | 0.000% | $0.00 | $0.00 | $361.42 | $6,999.41 | 0.00 | 0.00 | 13.93 |
| 21 | Distal Colon (includes descending, sigmoid colon, splenic flexure, rectum) | 0.000% | $0.00 | $0.00 | $361.42 | $6,999.41 | 0.00 | 0.00 | 13.93 |
| 21 | Colonoscopy TP | 0.000% | $0.00 | $0.00 | $361.42 | $6,999.41 | 0.00 | 0.00 | 13.93 |
| 21 | Colonoscopy FN | 0.000% | $0.00 | $0.00 | $361.42 | $6,999.41 | 0.00 | 0.00 | 13.93 |
| 21 | cancer | 0.000% | $0.00 | $0.00 | $361.42 | $6,999.41 | 0.00 | 0.00 | 13.93 |
| 21 | early | 0.000% | $0.00 | $0.00 | $361.42 | $6,999.41 | 0.00 | 0.00 | 13.93 |
| 21 | late | 0.000% | $0.00 | $0.00 | $361.42 | $6,999.41 | 0.00 | 0.00 | 13.93 |
| 21 | no cancer | 0.000% | $0.00 | $0.00 | $361.42 | $6,999.41 | 0.00 | 0.00 | 13.93 |
| 21 | Comply | 0.000% | $0.00 | $0.00 | $361.42 | $6,999.41 | 0.00 | 0.00 | 13.93 |
| 21 | Non-comply | 0.000% | $0.00 | $0.00 | $361.42 | $6,999.41 | 0.00 | 0.00 | 13.93 |
| 21 | Proximal colon (includes ascending, transverse) | 0.000% | $0.00 | $0.00 | $361.42 | $6,999.41 | 0.00 | 0.00 | 13.93 |
| 21 | Colonoscopy TP | 0.000% | $0.00 | $0.00 | $361.42 | $6,999.41 | 0.00 | 0.00 | 13.93 |
| 21 | Colonoscopy FN | 0.000% | $0.00 | $0.00 | $361.42 | $6,999.41 | 0.00 | 0.00 | 13.93 |
| 21 | cancer | 0.000% | $0.00 | $0.00 | $361.42 | $6,999.41 | 0.00 | 0.00 | 13.93 |
| 21 | early | 0.000% | $0.00 | $0.00 | $361.42 | $6,999.41 | 0.00 | 0.00 | 13.93 |
| 21 | late | 0.000% | $0.00 | $0.00 | $361.42 | $6,999.41 | 0.00 | 0.00 | 13.93 |
| 21 | no cancer | 0.000% | $0.00 | $0.00 | $361.42 | $6,999.41 | 0.00 | 0.00 | 13.93 |
| 21 | Comply | 0.000% | $0.00 | $0.00 | $361.42 | $6,999.41 | 0.00 | 0.00 | 13.93 |
| 21 | Non-comply | 0.000% | $0.00 | $0.00 | $361.42 | $6,999.41 | 0.00 | 0.00 | 13.93 |
| 21 | Inadequate Prep | 0.000% | $0.00 | $0.00 | $361.42 | $6,999.41 | 0.00 | 0.00 | 13.93 |
| 21 | PureVu usage | 0.000% | $0.00 | $0.00 | $361.42 | $6,999.41 | 0.00 | 0.00 | 13.93 |
| 21 | Colonoscopy FN | 0.000% | $0.00 | $0.00 | $361.42 | $6,999.41 | 0.00 | 0.00 | 13.93 |
| 21 | cancer | 0.000% | $0.00 | $0.00 | $361.42 | $6,999.41 | 0.00 | 0.00 | 13.93 |
| 21 | early | 0.000% | $0.00 | $0.00 | $361.42 | $6,999.41 | 0.00 | 0.00 | 13.93 |
| 21 | late | 0.000% | $0.00 | $0.00 | $361.42 | $6,999.41 | 0.00 | 0.00 | 13.93 |
| 21 | no cancer | 0.000% | $0.00 | $0.00 | $361.42 | $6,999.41 | 0.00 | 0.00 | 13.93 |
| 21 | Comply | 0.000% | $0.00 | $0.00 | $361.42 | $6,999.41 | 0.00 | 0.00 | 13.93 |
| 21 | Non-comply | 0.000% | $0.00 | $0.00 | $361.42 | $6,999.41 | 0.00 | 0.00 | 13.93 |
| 21 | No screening | 19.533% | $0.00 | $0.00 | $361.42 | $6,999.41 | 0.00 | 0.00 | 13.93 |
| 21 | Adenoma | 5.580% | $0.00 | $0.00 | $361.42 | $6,999.41 | 0.00 | 0.00 | 13.93 |
| 21 | Cancerous | 0.024% | $0.00 | $0.00 | $361.42 | $6,999.41 | 0.00 | 0.00 | 13.93 |
| 21 | Early | 0.020% | $0.00 | $0.00 | $361.42 | $6,999.41 | 0.00 | 0.00 | 13.93 |
| 21 | Advanced | 0.004% | $0.00 | $0.00 | $361.42 | $6,999.41 | 0.00 | 0.00 | 13.93 |
| 21 | Noncancerous | 5.556% | $0.00 | $0.00 | $361.42 | $6,999.41 | 0.00 | 0.00 | 13.93 |
| 21 | comply | 3.945% | $0.00 | $0.00 | $361.42 | $6,999.41 | 0.00 | 0.00 | 13.93 |
| 21 | Not comply | 1.611% | $0.00 | $0.00 | $361.42 | $6,999.41 | 0.00 | 0.00 | 13.93 |
| 21 | No adenoma | 7.296% | $0.00 | $0.00 | $361.42 | $6,999.41 | 0.00 | 0.00 | 13.93 |
| 21 | comply | 4.400% | $0.00 | $0.00 | $361.42 | $6,999.41 | 0.00 | 0.00 | 13.93 |
| 21 | Not comply | 2.897% | $0.00 | $0.00 | $361.42 | $6,999.41 | 0.00 | 0.00 | 13.93 |
| 21 | Continue | 0.019% | $0.00 | $0.00 | $361.42 | $6,999.41 | 0.00 | 0.00 | 13.93 |
| 21 | Die | 0.005% | $0.00 | $0.00 | $361.42 | $6,999.41 | 0.00 | 0.00 | 13.93 |
| 21 | PureVu usage | 11.107% | $0.00 | $0.00 | $361.42 | $6,999.41 | 0.00 | 0.00 | 13.93 |
| 21 | Later followup | 3.133% | $0.00 | $0.00 | $361.42 | $6,999.41 | 0.00 | 0.00 | 13.93 |
| 21 | Redo 2 years | 0.000% | $0.00 | $0.00 | $361.42 | $6,999.41 | 0.00 | 0.00 | 13.93 |
| 21 | comply | 0.000% | $0.00 | $0.00 | $361.42 | $6,999.41 | 0.00 | 0.00 | 13.93 |
| 21 | Not comply | 0.000% | $0.00 | $0.00 | $361.42 | $6,999.41 | 0.00 | 0.00 | 13.93 |
| 21 | No redo | 3.133% | $0.00 | $0.00 | $361.42 | $6,999.41 | 0.00 | 0.00 | 13.93 |
| 21 | Redo in at least 3 years | 13.255% | $0.00 | $0.00 | $361.42 | $6,999.41 | 0.00 | 0.00 | 13.93 |
| 21 | comply | 11.929% | $0.00 | $0.00 | $361.42 | $6,999.41 | 0.00 | 0.00 | 13.93 |
| 21 | Not comply | 1.325% | $0.00 | $0.00 | $361.42 | $6,999.41 | 0.00 | 0.00 | 13.93 |
| 21 | No redo | 0.000% | $0.00 | $0.00 | $361.42 | $6,999.41 | 0.00 | 0.00 | 13.93 |
| 21 | Adenoma | 0.000% | $0.00 | $0.00 | $361.42 | $6,999.41 | 0.00 | 0.00 | 13.93 |
| 21 | Cancerous | 0.000% | $0.00 | $0.00 | $361.42 | $6,999.41 | 0.00 | 0.00 | 13.93 |
| 21 | Early | 0.000% | $0.00 | $0.00 | $361.42 | $6,999.41 | 0.00 | 0.00 | 13.93 |
| 21 | Advanced | 0.000% | $0.00 | $0.00 | $361.42 | $6,999.41 | 0.00 | 0.00 | 13.93 |
| 21 | Noncancerous | 0.000% | $0.00 | $0.00 | $361.42 | $6,999.41 | 0.00 | 0.00 | 13.93 |
| 21 | No adenoma | 0.000% | $0.00 | $0.00 | $361.42 | $6,999.41 | 0.00 | 0.00 | 13.93 |
| 22 | comply | 42.939% | $0.00 | $0.00 | $128.14 | $7,127.55 | 0.47 | 0.20 | 14.37 |
| 22 | Non-comply post colonoscopy | 5.833% | $0.00 | $0.00 | $128.14 | $7,127.55 | 0.00 | 0.00 | 14.37 |
| 22 | Early CRC | 2.962% | $2,309.37 | $68.41 | $128.14 | $7,127.55 | 0.39 | 0.01 | 14.37 |
| 22 | Advanced CRC | 0.023% | $3,110.48 | $0.70 | $128.14 | $7,127.55 | 0.28 | 0.00 | 14.37 |
| 22 | Die other causes | 36.636% | $0.00 | $0.00 | $128.14 | $7,127.55 | 0.47 | 0.17 | 14.37 |
| 22 | Colonoscopy with PureVu due added to inadequate prep or not comply | 11.107% | $391.42 | $43.47 | $128.14 | $7,127.55 | 0.47 | 0.05 | 14.37 |
| 22 | Adenoma surveillance | 0.000% | $1,592.40 | $0.00 | $128.14 | $7,127.55 | 0.47 | 0.00 | 14.37 |
| 22 | Non-compliance with system | 0.000% | $0.00 | $0.00 | $128.14 | $7,127.55 | 0.00 | 0.00 | 14.37 |
| 22 | Dead | 0.500% | $3,110.48 | $15.56 | $128.14 | $7,127.55 | 0.00 | 0.00 | 14.37 |
| 22 | Screening every 10 years - average risk | 0.000% | $0.00 | $0.00 | $128.14 | $7,127.55 | 0.00 | 0.00 | 14.37 |
| 22 | Adequate Prep | 0.000% | $0.00 | $0.00 | $128.14 | $7,127.55 | 0.00 | 0.00 | 14.37 |
| 22 | no adenoma screening | 0.000% | $0.00 | $0.00 | $128.14 | $7,127.55 | 0.00 | 0.00 | 14.37 |
| 22 | Colonoscopy TN | 0.000% | $0.00 | $0.00 | $128.14 | $7,127.55 | 0.00 | 0.00 | 14.37 |
| 22 | Comply | 0.000% | $0.00 | $0.00 | $128.14 | $7,127.55 | 0.00 | 0.00 | 14.37 |
| 22 | Non-comply | 0.000% | $0.00 | $0.00 | $128.14 | $7,127.55 | 0.00 | 0.00 | 14.37 |
| 22 | Colonoscopy FP | 0.000% | $0.00 | $0.00 | $128.14 | $7,127.55 | 0.00 | 0.00 | 14.37 |
| 22 | adenoma | 0.000% | $0.00 | $0.00 | $128.14 | $7,127.55 | 0.00 | 0.00 | 14.37 |
| 22 | Distal Colon (includes descending, sigmoid colon, splenic flexure, rectum) | 0.000% | $0.00 | $0.00 | $128.14 | $7,127.55 | 0.00 | 0.00 | 14.37 |
| 22 | Colonoscopy TP | 0.000% | $0.00 | $0.00 | $128.14 | $7,127.55 | 0.00 | 0.00 | 14.37 |
| 22 | Colonoscopy FN | 0.000% | $0.00 | $0.00 | $128.14 | $7,127.55 | 0.00 | 0.00 | 14.37 |
| 22 | cancer | 0.000% | $0.00 | $0.00 | $128.14 | $7,127.55 | 0.00 | 0.00 | 14.37 |
| 22 | early | 0.000% | $0.00 | $0.00 | $128.14 | $7,127.55 | 0.00 | 0.00 | 14.37 |
| 22 | late | 0.000% | $0.00 | $0.00 | $128.14 | $7,127.55 | 0.00 | 0.00 | 14.37 |
| 22 | no cancer | 0.000% | $0.00 | $0.00 | $128.14 | $7,127.55 | 0.00 | 0.00 | 14.37 |
| 22 | Comply | 0.000% | $0.00 | $0.00 | $128.14 | $7,127.55 | 0.00 | 0.00 | 14.37 |
| 22 | Non-comply | 0.000% | $0.00 | $0.00 | $128.14 | $7,127.55 | 0.00 | 0.00 | 14.37 |
| 22 | Proximal colon (includes ascending, transverse) | 0.000% | $0.00 | $0.00 | $128.14 | $7,127.55 | 0.00 | 0.00 | 14.37 |
| 22 | Colonoscopy TP | 0.000% | $0.00 | $0.00 | $128.14 | $7,127.55 | 0.00 | 0.00 | 14.37 |
| 22 | Colonoscopy FN | 0.000% | $0.00 | $0.00 | $128.14 | $7,127.55 | 0.00 | 0.00 | 14.37 |
| 22 | cancer | 0.000% | $0.00 | $0.00 | $128.14 | $7,127.55 | 0.00 | 0.00 | 14.37 |
| 22 | early | 0.000% | $0.00 | $0.00 | $128.14 | $7,127.55 | 0.00 | 0.00 | 14.37 |
| 22 | late | 0.000% | $0.00 | $0.00 | $128.14 | $7,127.55 | 0.00 | 0.00 | 14.37 |
| 22 | no cancer | 0.000% | $0.00 | $0.00 | $128.14 | $7,127.55 | 0.00 | 0.00 | 14.37 |
| 22 | Comply | 0.000% | $0.00 | $0.00 | $128.14 | $7,127.55 | 0.00 | 0.00 | 14.37 |
| 22 | Non-comply | 0.000% | $0.00 | $0.00 | $128.14 | $7,127.55 | 0.00 | 0.00 | 14.37 |
| 22 | Inadequate Prep | 0.000% | $0.00 | $0.00 | $128.14 | $7,127.55 | 0.00 | 0.00 | 14.37 |
| 22 | PureVu usage | 0.000% | $0.00 | $0.00 | $128.14 | $7,127.55 | 0.00 | 0.00 | 14.37 |
| 22 | Colonoscopy FN | 0.000% | $0.00 | $0.00 | $128.14 | $7,127.55 | 0.00 | 0.00 | 14.37 |
| 22 | cancer | 0.000% | $0.00 | $0.00 | $128.14 | $7,127.55 | 0.00 | 0.00 | 14.37 |
| 22 | early | 0.000% | $0.00 | $0.00 | $128.14 | $7,127.55 | 0.00 | 0.00 | 14.37 |
| 22 | late | 0.000% | $0.00 | $0.00 | $128.14 | $7,127.55 | 0.00 | 0.00 | 14.37 |
| 22 | no cancer | 0.000% | $0.00 | $0.00 | $128.14 | $7,127.55 | 0.00 | 0.00 | 14.37 |
| 22 | Comply | 0.000% | $0.00 | $0.00 | $128.14 | $7,127.55 | 0.00 | 0.00 | 14.37 |
| 22 | Non-comply | 0.000% | $0.00 | $0.00 | $128.14 | $7,127.55 | 0.00 | 0.00 | 14.37 |
| 22 | No screening | 42.939% | $0.00 | $0.00 | $128.14 | $7,127.55 | 0.00 | 0.00 | 14.37 |
| 22 | Adenoma | 2.528% | $0.00 | $0.00 | $128.14 | $7,127.55 | 0.00 | 0.00 | 14.37 |
| 22 | Cancerous | 0.011% | $0.00 | $0.00 | $128.14 | $7,127.55 | 0.00 | 0.00 | 14.37 |
| 22 | Early | 0.009% | $0.00 | $0.00 | $128.14 | $7,127.55 | 0.00 | 0.00 | 14.37 |
| 22 | Advanced | 0.002% | $0.00 | $0.00 | $128.14 | $7,127.55 | 0.00 | 0.00 | 14.37 |
| 22 | Noncancerous | 2.517% | $0.00 | $0.00 | $128.14 | $7,127.55 | 0.00 | 0.00 | 14.37 |
| 22 | comply | 1.787% | $0.00 | $0.00 | $128.14 | $7,127.55 | 0.00 | 0.00 | 14.37 |
| 22 | Not comply | 0.730% | $0.00 | $0.00 | $128.14 | $7,127.55 | 0.00 | 0.00 | 14.37 |
| 22 | No adenoma | 3.306% | $0.00 | $0.00 | $128.14 | $7,127.55 | 0.00 | 0.00 | 14.37 |
| 22 | comply | 1.993% | $0.00 | $0.00 | $128.14 | $7,127.55 | 0.00 | 0.00 | 14.37 |
| 22 | Not comply | 1.312% | $0.00 | $0.00 | $128.14 | $7,127.55 | 0.00 | 0.00 | 14.37 |
| 22 | Continue | 0.018% | $0.00 | $0.00 | $128.14 | $7,127.55 | 0.00 | 0.00 | 14.37 |
| 22 | Die | 0.005% | $0.00 | $0.00 | $128.14 | $7,127.55 | 0.00 | 0.00 | 14.37 |
| 22 | PureVu usage | 8.663% | $0.00 | $0.00 | $128.14 | $7,127.55 | 0.00 | 0.00 | 14.37 |
| 22 | Later followup | 2.443% | $0.00 | $0.00 | $128.14 | $7,127.55 | 0.00 | 0.00 | 14.37 |
| 22 | Redo 2 years | 2.443% | $0.00 | $0.00 | $128.14 | $7,127.55 | 0.00 | 0.00 | 14.37 |
| 22 | comply | 1.366% | $0.00 | $0.00 | $128.14 | $7,127.55 | 0.00 | 0.00 | 14.37 |
| 22 | Not comply | 1.078% | $0.00 | $0.00 | $128.14 | $7,127.55 | 0.00 | 0.00 | 14.37 |
| 22 | No redo | 0.000% | $0.00 | $0.00 | $128.14 | $7,127.55 | 0.00 | 0.00 | 14.37 |
| 22 | Redo in at least 3 years | 0.000% | $0.00 | $0.00 | $128.14 | $7,127.55 | 0.00 | 0.00 | 14.37 |
| 22 | comply | 0.000% | $0.00 | $0.00 | $128.14 | $7,127.55 | 0.00 | 0.00 | 14.37 |
| 22 | Not comply | 0.000% | $0.00 | $0.00 | $128.14 | $7,127.55 | 0.00 | 0.00 | 14.37 |
| 22 | No redo | 0.000% | $0.00 | $0.00 | $128.14 | $7,127.55 | 0.00 | 0.00 | 14.37 |
| 22 | Adenoma | 0.000% | $0.00 | $0.00 | $128.14 | $7,127.55 | 0.00 | 0.00 | 14.37 |
| 22 | Cancerous | 0.000% | $0.00 | $0.00 | $128.14 | $7,127.55 | 0.00 | 0.00 | 14.37 |
| 22 | Early | 0.000% | $0.00 | $0.00 | $128.14 | $7,127.55 | 0.00 | 0.00 | 14.37 |
| 22 | Advanced | 0.000% | $0.00 | $0.00 | $128.14 | $7,127.55 | 0.00 | 0.00 | 14.37 |
| 22 | Noncancerous | 0.000% | $0.00 | $0.00 | $128.14 | $7,127.55 | 0.00 | 0.00 | 14.37 |
| 22 | No adenoma | 0.000% | $0.00 | $0.00 | $128.14 | $7,127.55 | 0.00 | 0.00 | 14.37 |
| 23 | comply | 48.085% | $0.00 | $0.00 | $115.37 | $7,242.92 | 0.46 | 0.22 | 14.81 |
| 23 | Non-comply post colonoscopy | 3.120% | $0.00 | $0.00 | $115.37 | $7,242.92 | 0.00 | 0.00 | 14.81 |
| 23 | Early CRC | 2.971% | $2,242.11 | $66.62 | $115.37 | $7,242.92 | 0.37 | 0.01 | 14.81 |
| 23 | Advanced CRC | 0.020% | $3,019.88 | $0.60 | $115.37 | $7,242.92 | 0.27 | 0.00 | 14.81 |
| 23 | Die other causes | 36.636% | $0.00 | $0.00 | $115.37 | $7,242.92 | 0.46 | 0.17 | 14.81 |
| 23 | Colonoscopy with PureVu due added to inadequate prep or not comply | 8.663% | $380.02 | $32.92 | $115.37 | $7,242.92 | 0.46 | 0.04 | 14.81 |
| 23 | Adenoma surveillance | 0.000% | $1,546.02 | $0.00 | $115.37 | $7,242.92 | 0.46 | 0.00 | 14.81 |
| 23 | Non-compliance with system | 0.000% | $0.00 | $0.00 | $115.37 | $7,242.92 | 0.00 | 0.00 | 14.81 |
| 23 | Dead | 0.505% | $3,019.88 | $15.24 | $115.37 | $7,242.92 | 0.00 | 0.00 | 14.81 |
| 23 | Screening every 10 years - average risk | 0.000% | $0.00 | $0.00 | $115.37 | $7,242.92 | 0.00 | 0.00 | 14.81 |
| 23 | Adequate Prep | 0.000% | $0.00 | $0.00 | $115.37 | $7,242.92 | 0.00 | 0.00 | 14.81 |
| 23 | no adenoma screening | 0.000% | $0.00 | $0.00 | $115.37 | $7,242.92 | 0.00 | 0.00 | 14.81 |
| 23 | Colonoscopy TN | 0.000% | $0.00 | $0.00 | $115.37 | $7,242.92 | 0.00 | 0.00 | 14.81 |
| 23 | Comply | 0.000% | $0.00 | $0.00 | $115.37 | $7,242.92 | 0.00 | 0.00 | 14.81 |
| 23 | Non-comply | 0.000% | $0.00 | $0.00 | $115.37 | $7,242.92 | 0.00 | 0.00 | 14.81 |
| 23 | Colonoscopy FP | 0.000% | $0.00 | $0.00 | $115.37 | $7,242.92 | 0.00 | 0.00 | 14.81 |
| 23 | adenoma | 0.000% | $0.00 | $0.00 | $115.37 | $7,242.92 | 0.00 | 0.00 | 14.81 |
| 23 | Distal Colon (includes descending, sigmoid colon, splenic flexure, rectum) | 0.000% | $0.00 | $0.00 | $115.37 | $7,242.92 | 0.00 | 0.00 | 14.81 |
| 23 | Colonoscopy TP | 0.000% | $0.00 | $0.00 | $115.37 | $7,242.92 | 0.00 | 0.00 | 14.81 |
| 23 | Colonoscopy FN | 0.000% | $0.00 | $0.00 | $115.37 | $7,242.92 | 0.00 | 0.00 | 14.81 |
| 23 | cancer | 0.000% | $0.00 | $0.00 | $115.37 | $7,242.92 | 0.00 | 0.00 | 14.81 |
| 23 | early | 0.000% | $0.00 | $0.00 | $115.37 | $7,242.92 | 0.00 | 0.00 | 14.81 |
| 23 | late | 0.000% | $0.00 | $0.00 | $115.37 | $7,242.92 | 0.00 | 0.00 | 14.81 |
| 23 | no cancer | 0.000% | $0.00 | $0.00 | $115.37 | $7,242.92 | 0.00 | 0.00 | 14.81 |
| 23 | Comply | 0.000% | $0.00 | $0.00 | $115.37 | $7,242.92 | 0.00 | 0.00 | 14.81 |
| 23 | Non-comply | 0.000% | $0.00 | $0.00 | $115.37 | $7,242.92 | 0.00 | 0.00 | 14.81 |
| 23 | Proximal colon (includes ascending, transverse) | 0.000% | $0.00 | $0.00 | $115.37 | $7,242.92 | 0.00 | 0.00 | 14.81 |
| 23 | Colonoscopy TP | 0.000% | $0.00 | $0.00 | $115.37 | $7,242.92 | 0.00 | 0.00 | 14.81 |
| 23 | Colonoscopy FN | 0.000% | $0.00 | $0.00 | $115.37 | $7,242.92 | 0.00 | 0.00 | 14.81 |
| 23 | cancer | 0.000% | $0.00 | $0.00 | $115.37 | $7,242.92 | 0.00 | 0.00 | 14.81 |
| 23 | early | 0.000% | $0.00 | $0.00 | $115.37 | $7,242.92 | 0.00 | 0.00 | 14.81 |
| 23 | late | 0.000% | $0.00 | $0.00 | $115.37 | $7,242.92 | 0.00 | 0.00 | 14.81 |
| 23 | no cancer | 0.000% | $0.00 | $0.00 | $115.37 | $7,242.92 | 0.00 | 0.00 | 14.81 |
| 23 | Comply | 0.000% | $0.00 | $0.00 | $115.37 | $7,242.92 | 0.00 | 0.00 | 14.81 |
| 23 | Non-comply | 0.000% | $0.00 | $0.00 | $115.37 | $7,242.92 | 0.00 | 0.00 | 14.81 |
| 23 | Inadequate Prep | 0.000% | $0.00 | $0.00 | $115.37 | $7,242.92 | 0.00 | 0.00 | 14.81 |
| 23 | PureVu usage | 0.000% | $0.00 | $0.00 | $115.37 | $7,242.92 | 0.00 | 0.00 | 14.81 |
| 23 | Colonoscopy FN | 0.000% | $0.00 | $0.00 | $115.37 | $7,242.92 | 0.00 | 0.00 | 14.81 |
| 23 | cancer | 0.000% | $0.00 | $0.00 | $115.37 | $7,242.92 | 0.00 | 0.00 | 14.81 |
| 23 | early | 0.000% | $0.00 | $0.00 | $115.37 | $7,242.92 | 0.00 | 0.00 | 14.81 |
| 23 | late | 0.000% | $0.00 | $0.00 | $115.37 | $7,242.92 | 0.00 | 0.00 | 14.81 |
| 23 | no cancer | 0.000% | $0.00 | $0.00 | $115.37 | $7,242.92 | 0.00 | 0.00 | 14.81 |
| 23 | Comply | 0.000% | $0.00 | $0.00 | $115.37 | $7,242.92 | 0.00 | 0.00 | 14.81 |
| 23 | Non-comply | 0.000% | $0.00 | $0.00 | $115.37 | $7,242.92 | 0.00 | 0.00 | 14.81 |
| 23 | No screening | 48.085% | $0.00 | $0.00 | $115.37 | $7,242.92 | 0.00 | 0.00 | 14.81 |
| 23 | Adenoma | 1.352% | $0.00 | $0.00 | $115.37 | $7,242.92 | 0.00 | 0.00 | 14.81 |
| 23 | Cancerous | 0.006% | $0.00 | $0.00 | $115.37 | $7,242.92 | 0.00 | 0.00 | 14.81 |
| 23 | Early | 0.005% | $0.00 | $0.00 | $115.37 | $7,242.92 | 0.00 | 0.00 | 14.81 |
| 23 | Advanced | 0.001% | $0.00 | $0.00 | $115.37 | $7,242.92 | 0.00 | 0.00 | 14.81 |
| 23 | Noncancerous | 1.346% | $0.00 | $0.00 | $115.37 | $7,242.92 | 0.00 | 0.00 | 14.81 |
| 23 | comply | 0.956% | $0.00 | $0.00 | $115.37 | $7,242.92 | 0.00 | 0.00 | 14.81 |
| 23 | Not comply | 0.390% | $0.00 | $0.00 | $115.37 | $7,242.92 | 0.00 | 0.00 | 14.81 |
| 23 | No adenoma | 1.768% | $0.00 | $0.00 | $115.37 | $7,242.92 | 0.00 | 0.00 | 14.81 |
| 23 | comply | 1.066% | $0.00 | $0.00 | $115.37 | $7,242.92 | 0.00 | 0.00 | 14.81 |
| 23 | Not comply | 0.702% | $0.00 | $0.00 | $115.37 | $7,242.92 | 0.00 | 0.00 | 14.81 |
| 23 | Continue | 0.016% | $0.00 | $0.00 | $115.37 | $7,242.92 | 0.00 | 0.00 | 14.81 |
| 23 | Die | 0.004% | $0.00 | $0.00 | $115.37 | $7,242.92 | 0.00 | 0.00 | 14.81 |
| 23 | PureVu usage | 6.757% | $0.00 | $0.00 | $115.37 | $7,242.92 | 0.00 | 0.00 | 14.81 |
| 23 | Later followup | 1.906% | $0.00 | $0.00 | $115.37 | $7,242.92 | 0.00 | 0.00 | 14.81 |
| 23 | Redo 2 years | 0.000% | $0.00 | $0.00 | $115.37 | $7,242.92 | 0.00 | 0.00 | 14.81 |
| 23 | comply | 0.000% | $0.00 | $0.00 | $115.37 | $7,242.92 | 0.00 | 0.00 | 14.81 |
| 23 | Not comply | 0.000% | $0.00 | $0.00 | $115.37 | $7,242.92 | 0.00 | 0.00 | 14.81 |
| 23 | No redo | 1.906% | $0.00 | $0.00 | $115.37 | $7,242.92 | 0.00 | 0.00 | 14.81 |
| 23 | Redo in at least 3 years | 0.000% | $0.00 | $0.00 | $115.37 | $7,242.92 | 0.00 | 0.00 | 14.81 |
| 23 | comply | 0.000% | $0.00 | $0.00 | $115.37 | $7,242.92 | 0.00 | 0.00 | 14.81 |
| 23 | Not comply | 0.000% | $0.00 | $0.00 | $115.37 | $7,242.92 | 0.00 | 0.00 | 14.81 |
| 23 | No redo | 0.000% | $0.00 | $0.00 | $115.37 | $7,242.92 | 0.00 | 0.00 | 14.81 |
| 23 | Adenoma | 0.000% | $0.00 | $0.00 | $115.37 | $7,242.92 | 0.00 | 0.00 | 14.81 |
| 23 | Cancerous | 0.000% | $0.00 | $0.00 | $115.37 | $7,242.92 | 0.00 | 0.00 | 14.81 |
| 23 | Early | 0.000% | $0.00 | $0.00 | $115.37 | $7,242.92 | 0.00 | 0.00 | 14.81 |
| 23 | Advanced | 0.000% | $0.00 | $0.00 | $115.37 | $7,242.92 | 0.00 | 0.00 | 14.81 |
| 23 | Noncancerous | 0.000% | $0.00 | $0.00 | $115.37 | $7,242.92 | 0.00 | 0.00 | 14.81 |
| 23 | No adenoma | 0.000% | $0.00 | $0.00 | $115.37 | $7,242.92 | 0.00 | 0.00 | 14.81 |
| 24 | comply | 52.013% | $0.00 | $0.00 | $702.09 | $7,945.01 | 0.44 | 0.23 | 15.25 |
| 24 | Non-comply post colonoscopy | 1.092% | $0.00 | $0.00 | $702.09 | $7,945.01 | 0.00 | 0.00 | 15.25 |
| 24 | Early CRC | 2.976% | $8,284.16 | $246.55 | $702.09 | $7,945.01 | 0.39 | 0.01 | 15.25 |
| 24 | Advanced CRC | 0.017% | $81,990.60 | $13.64 | $702.09 | $7,945.01 | 0.41 | 0.00 | 15.25 |
| 24 | Die other causes | 36.636% | $0.00 | $0.00 | $702.09 | $7,945.01 | 0.45 | 0.16 | 15.25 |
| 24 | Colonoscopy with PureVu due to inadequate prep or not comply | 6.757% | $368.95 | $24.93 | $702.09 | $7,945.01 | 0.45 | 0.03 | 15.25 |
| 24 | Adenoma surveillance | 0.000% | $1,500.99 | $0.00 | $702.09 | $7,945.01 | 0.45 | 0.00 | 15.25 |
| 24 | Non-compliance with system | 0.000% | $0.00 | $0.00 | $702.09 | $7,945.01 | 0.00 | 0.00 | 15.25 |
| 24 | Dead | 0.509% | $81,990.60 | $416.97 | $702.09 | $7,945.01 | 0.00 | 0.00 | 15.25 |
